# A New Personalized Oral Cancer Survival Calculator to Estimate Risk of Death From Both Oral Cancer and Other Causes

**DOI:** 10.1001/jamaoto.2023.1975

**Published:** 2023-07-10

**Authors:** Louise Davies, Benjamin F. Hankey, Zhuoqiao Wang, Zhaohui Zou, Susan Scott, Minjung Lee, Hyunsoon Cho, Eric J. Feuer

**Affiliations:** 1 VA Outcomes Group, Department of Veterans Affairs Medical Center, White River Junction, Vermont; 2 Section of Otolaryngology in Geisel School of Medicine at Dartmouth, and The Dartmouth Institute for Health Policy and Clinical Practice, Lebanon, New Hampshire; 3 Statistical Research and Application Branch, Surveillance Research Program, Division of Cancer Control and Population Sciences, National Cancer Institute, Bethesda, Maryland; 4 Information Management Services, Calverton, Maryland; 5 Surveillance Research Program, Division of Cancer Control and Population Sciences, National Cancer Institute, Bethesda, Maryland; 6 Department of Statistics, Kangwon National University, Chuncheon, Gangwon, Korea; 7 Department of Cancer AI and Digital Health, National Cancer Center Graduate School of Cancer Science and Policy, and the Integrated Biostatistics Branch, Division of Cancer Data Science, National Cancer Center, Goyang, Gyeonggi-do, Korea

## Abstract

**Importance:**

Standard cancer prognosis models typically do not include much specificity in characterizing competing illnesses or general health status when providing prognosis estimates, limiting their utility for individuals, who must consider their cancer in the context of their overall health. This is especially true for patients with oral cancer, who frequently have competing illnesses.

**Objective:**

To describe a statistical framework and accompanying new publicly available calculator that provides personalized estimates of the probability of a patient surviving or dying from cancer or other causes, using oral cancer as the first data set.

**Design, Setting, and Participants:**

The models used data from the Surveillance, Epidemiology, and End Results (SEER) 18 registry (2000 to 2011), SEER-Medicare linked files, and the National Health Interview Survey (NHIS) (1986 to 2009). Statistical methods developed to calculate natural life expectancy in the absence of the cancer, cancer-specific survival, and other-cause survival were applied to oral cancer data and internally validated with 10-fold cross-validation. Eligible participants were aged between 20 and 94 years with oral squamous cell carcinoma.

**Exposures:**

Histologically confirmed oral cancer, general health status, smoking, and selected serious comorbid conditions.

**Main Outcomes and Measures:**

Probabilities of surviving or dying from the cancer or from other causes, and life expectancy in the absence of the cancer.

**Results:**

A total of 22 392 patients with oral squamous cell carcinoma (13 544 male [60.5%]; 1476 Asian and Pacific Islander [6.7%]; 1792 Black [8.0%], 1589 Hispanic [7.2%], 17 300 White [78.1%]) and 402 626 NHIS interviewees were included in this calculator designed for public use for patients ages 20 to 86 years with newly diagnosed oral cancer to obtain estimates of health status–adjusted age, life expectancy in the absence of the cancer, and the probability of surviving, dying from the cancer, or dying from other causes within 1 to 10 years after diagnosis. The models in the calculator estimated that patients with oral cancer have a higher risk of death from other causes than their matched US population, and that this risk increases by stage.

**Conclusions and relevance:**

The models developed for the calculator demonstrate that survival estimates that exclude the effects of coexisting conditions can lead to underestimates or overestimates of survival. This new calculator approach will be broadly applicable for developing future prognostic models of cancer and noncancer aspects of a person’s health in other cancers; as registries develop more linkages, available covariates will become broader, strengthening future tools.

## Key Points

**Question:**

How does the competing risk of death from noncancer causes affect risk of death from oral cancer?

**Findings:**

The models in the calculator using health data of 22 392 US adults with confirmed oral cancer estimates that patients with oral cancer have a higher risk of death from other causes than a matched US population, and that this risk increases by stage.

**Meaning:**

These results suggest that survival estimates that exclude the effects of coexisting conditions can lead to under- or overestimates of survival; this tool provides personalized data for discussions about the place of cancer treatment in the patient’s life as a whole.

## Teaser

This decision analytical model study of US adults with oral cancer uses health records linked with Medicare files to model an oral cancer survival calculator accounting for other health-related causes of death.

## Introduction

When a person receives a new cancer diagnosis, a natural tendency is to focus on the cancer as the main threat to survival. But the person may have other conditions that pose an equal or even greater threat than their cancer—a competing risk of death. In practice, if the person has quite severe comorbid or coexisting conditions, even a relatively serious cancer diagnosis may not end up being the cause of their death. Competing risks are particularly important for cancers in which risk factors for the cancer can also cause coexisting conditions. Personalized data on the competing risks to a person’s survival could provide more highly contextualized data for the patient and their clinician as they discuss cancer treatment options.

To develop this first calculator, we chose oral cancer because prolonged exposures to alcohol and tobacco are risk factors for cancer in this location, but also can result in medical conditions with the potential to shorten life expectancy,^1,2,3^ competing as a cause of death that may intervene in conjunction with or before the cancer.^4,5^ The resulting product of this work is the Surveillance, Epidemiology, and End Results Oral Cancer Survival Calculator (SEER OCSC).^6^ It provides personalized estimates of the likelihood of surviving or dying from oral cancer or other causes, for newly diagnosed patients ages 20 to 86 years. It is not intended for patients with cancers of the tonsil and tongue base, which are epidemiologically distinct and not anatomically part of the oral cavity. Users are guided through 1 of 3 pathways based on their age and the clinical data available about their general health status. The main aim of this article was to inform clinicians of the SEER OCSC’s purpose and provide a brief description of the statistical framework and the underlying data. Readers are encouraged to access the accompanying Special Communication for key points on clinical use of the tool.^7^

## Methods

The Veterans Institutional Review Board of Northern New England reviewed and approved this study as exempt from human participants review under category 2 for the portions involving human participants and category 4 for the data. The data used in this study are available through signed data use agreements with the relevant managing agencies. For more information, please visit the websites for SEER, the National Cancer Institute’s Healthcare Delivery Research Program, or the US Centers for Disease Control and Prevention.

### Data Sources

**Figure 1.**
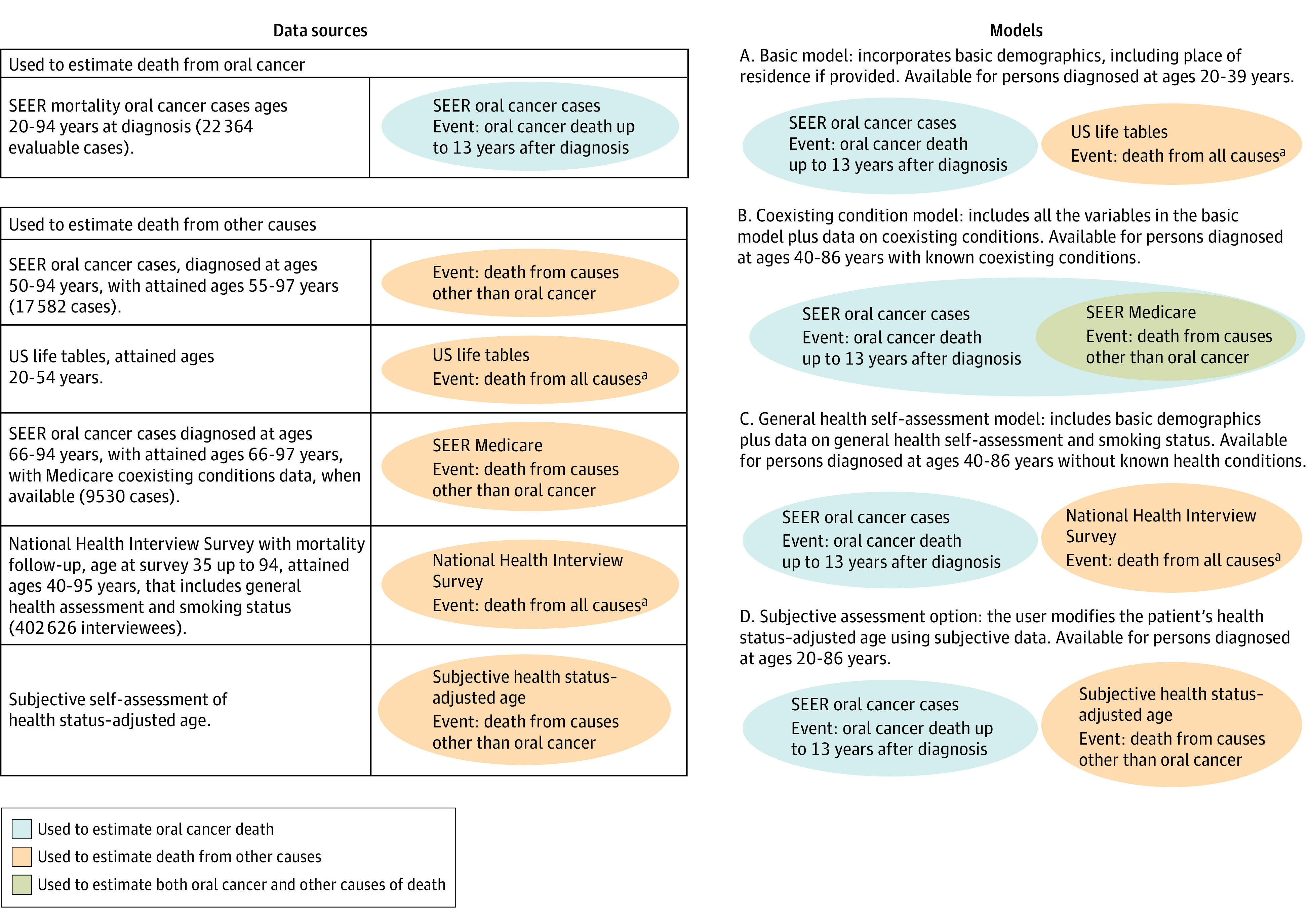
Data Sources and Models in the SEER Oral Cancer Survival Calculator ^a^So few people die of oral cancer that death from all causes is used as an approximation of death from other causes. SEER indicates Surveillance, Epidemiology and End Results.

Cancer data were from SEER 18 (2000-2011), and US life tables by race, ethnicity, and sex are from 2011 from the National Center for Health Statistics (Figure 1).^8,9^ SEER-Medicare linked data were used to obtain coexisting condition information for ages 66 years and above. That information was broadened by adding data from persons aged 35 to 94 years who were interviewed in the National Health Interview Survey (NHIS)^10^ 1986 to 2009 and provided data on their smoking status and general health self-assessment. While NHIS represents the general population, by conditioning on salient covariates it becomes applicable to patients with oral cancer. Details about the SEER, SEER-Medicare, and NHIS—Linked Mortality File (NHIS-LMF) data sources are included in eAppendix 1 in Supplement 1.

### Statistical Analysis

#### Two Time Scale Competing Risks Modeling

The SEER OCSC system creates personalized models to estimate prognosis utilizing competing risks models. In this case there are 2 possible events, death from oral cancer and death from other causes. The calculator uses 2 time scales at the same time in 1 analysis: for death due to other causes, the time scale is age; for cancer, the time scale is time since diagnosis. Our novel methodology is described in detail in eMethods and eFigure 1 in Supplement 1. Briefly, Lee et al^11^ developed prediction methods in continuous time for competing risks data using 2 different time scales (time since diagnosis for cancer death and age for other-cause deaths), extending the work of Cheng et al.^12^ Lee et al then developed discrete time methods (more commonly used for large cancer registry data sets) for competing risks predictions on both a single time scale and 2 time scales.^13^ The work was further extended by clarifying how to conduct the modeling if each cause of death was modeled using independent or using partially overlapping data sources, and using data sources with nonstandard sampling schemes.^14^ All of these modifications allowed flexibility to model competing risks in novel ways, taking advantage of available data as well as the complexity of the risk factors and their associations with the risk of the 2 event types.

Each competing risks of death model specified separate sets of covariates to estimate the risk of oral cancer death and the risk of death from a cause other than oral cancer. The SEER-developed, cause-specific death classification (Howlader et al^15^) was used to identify the oral cancer deaths and deaths from other causes to be included in the survival analyses.

#### Models

First, we describe the model for estimating the probability of death due to oral cancer, and then we describe the 3 models for estimating the probability of death due to other causes (Figure 1), which varied with respect to the data and covariates included. Model details are in eAppendix 3, eTable 5, and eFigure 1 in Supplement 1 and model estimation details are in eTables 6-8 in Supplement 1.

The probability of death due to oral cancer was estimated using SEER data and was stratified by stage (according to the *American Joint Committee on Cancer Cancer Staging Manual, Sixth Edition*^16^ [AJCC 6]). The other covariates were tumor grade, whether the tumor and lymph node status was diagnosed clinically or pathologically, sex, age at diagnosis (fit as a restricted cubic spline), race and ethnicity, socioeconomic status of the census tract of residence^17,18,19^ (in quintiles based only on persons with oral cancer), marital status, calendar year of diagnosis (grouped as 2000-2003, 2004-2007, and 2008-2011), and a variable for those age 66 years and above that indicated whether the person was in poor health just before diagnosis (eTables 1-4 in Supplement 1).

There are 3 models for estimating the probability of death due to other causes depending on the patient’s age and the availability of health information. Estimates for patients who are ages 20 to 39 years were from the basic model, which used only demographic and staging data as described above for SEER oral cancer cases. Estimates for patients ages 66 to 86 years who see a clinician regularly for their general health care prior to their cancer diagnosis were from the coexisting conditions model, which was fit using SEER-Medicare linked data that added to the basic model a composite measure of the type and severity of 14 specifically recognized coexisting conditions affecting life expectancy.^20,21^ Estimates for patients ages 40 to 65 years, or those aged 66 years and older who do not see a clinician regularly for their general health care prior to their cancer diagnosis were from the general health self-assessment model. This model was fit using data from the National Health Interview Survey, a general survey of the US population with mortality follow-up. This model was an adaptation of an earlier model by Cho et al.^22^ Internal validation of the models was assessed using calibration plots^23^ and time-dependent area under the receiver operating characteristic curve based on 10-fold cross-validation,^24^ as described in eAppendix 6, eFigures 9-24, and eTables 19-21 in Supplement 1.

#### Metrics Arising From the Models and Model Variations

For each of the 3 models used for estimating the probability of death due to other causes in the calculator, a set of 2 related metrics is calculated: health status–adjusted age and life expectancy without cancer. Health status–adjusted age adds or subtracts from a person’s chronologic age to account for good health or poor health prior to their cancer diagnosis relative to an average person of the same sex in the US population. Based on each of these 3 models, the predicted cumulative mortality is estimated for a person with a specific covariate profile. This cumulative mortality curve in the absence of cancer is then used to calculate life expectancy. This modeled life expectancy is compared with life expectancies in a sex-specific US life table, and the age in the life table with the closest life expectancy is considered the health status–adjusted age for that individual. To ensure internal consistency between the person’s health status–adjusted age and their cumulative cause-specific estimates of dying in the next 10 years, the interval probabilities of death from the life table were substituted for the modeled estimates (eAppendix 2 in Supplement 1).

If the user feels that the calculated health status–adjusted age does not fully capture the person’s health status, the user can modify the age up or down manually, which adjusts the survival estimates accordingly. SAS version 9.4 (SAS Institute) was used for the models in both the basic model and the coexisting condition model. PROC LOGISTIC was used for variable selection, and the final model was fit using with PROC GENMOD. The Stata version 13 (StataCorp) svy:glm command was used to incorporate the complex survey design and weights of NHIS used to estimate the general health self-assessment model. The R code for the calculation of the cumulative crude dying probabilities due to oral cancer and other causes is provided in the supplement to Lee et al.^14^ The statistical significance for coefficients in the models were computed using Wald tests. For more details, see eAppendix3 in Supplement 1.

#### User Interface Development and Testing

The web conceptualization for the calculator arose from an analysis of the strengths and weaknesses of existing tools^25^ followed by input to develop the use case scenario from an advisory panel of 4 physicians and 1 patient with oral cancer. A beta version was created and subjected to internal and external review by National Cancer Institute statistical and cancer survivorship reviewers and 2 rounds of focus group testing and revision with 10 patients and 5 physicians from 2 major US oral cancer patient advocacy groups, the Head & Neck Cancer Alliance, and SPOHNC (Support for People with Oral and Head & Neck Cancer). The feedback was used to create the final version of the calculator for public release, ensuring it reflects the specific needs of clinicians and newly diagnosed patients. Two major changes made were (1) warnings were added to the landing page about the provision of survival data, which not all patients want to access at the time of diagnosis, and (2) the overall design of the system was completely changed—users are guided to the best model for obtaining estimates, instead of choosing which model to use. Numerous small clarifications of language and links to external resources were also added.

## Results

Of a total 22 392 included patients, 13 544 (60.5%) were male; 1476 patients (6.7%) were Asian and Pacific Islander, 1792 (8.0%) Black, 1589 (7.2%) Hispanic, and 17 300 (78.1%) White (Table). The mean age at diagnosis for persons who had stage I cancer was 61.6 years, stage II was 64.7 years, stage III was 62.9 years, and stage IV was 62.3 years. Comorbidity frequencies were estimated from SEER data linked to the Medicare data at the Centers for Medicare & Medicaid Services and are presented in eTable 3 in Supplement 1. The most common coexisting conditions were chronic obstructive pulmonary disease and diabetes. Chronic obstructive pulmonary disease occurred for 1081 of 6181 patients (17.5%) in the oral cancer cohort age 66 years and older, while in the Medicare population more broadly, as shown by Cho et al,^27^ the rate starts at only 6% and increases to 11% by age 90 years or greater. In the oral cancer cohort, only 52.8% of patients had none of the major recorded coexisting conditions. In comparison, at age 65 years, 80% of the Medicare population has no recorded coexisting conditions.^27^ The percentage falls to 60% with no coexisting conditions by age 90 years and older, but this proportion is still lower than in the oral cancer cohort.^27^

**Table.** Characteristics of Patients Included in the SEER Oral Cancer Survival Calculator

Characteristic	Patients, No. (%) (N = 22 392)^a^
Sex	
Female	8848 (39.5)
Male	13 544 (60.5)
Age group, y	
20-44	1976 (8.8)
45-54	4348 (19.4)
55-65	6538 (29.2)
66-74	4339 (19.4)
75-84	3732 (16.7)
85-94	1459 (6.5)^a^
Race and ethnicity	
Hispanic	1589 (7.2)
Non-Hispanic Black	1792 (8.0)
Non-Hispanic Asian and Pacific Islander	1476 (6.7)
Non-Hispanic White	17 300 (78.1)
Cancer subsite	
Mobile tongue	11 025 (49.2)
Floor of mouth	4469 (19.9)
Retromolar trigone	1411 (6.3)
Other mucosa, gums, hard palate	5487 (24.5)
Stage at diagnosis (AJCC 6)	
I	6152 (34.1)
II	3182 (17.6)
III	2895 (16.1)
IV	5800 (32.2)

Abbreviations: AJCC 6, *American Joint Committee on Cancer Cancer Staging Manual, Sixth Edition*; SEER, Surveillance, Epidemiology, and End Results.

^a^ Cases were drawn from years 2000-2011, ages 20-94 years at diagnosis, (*International Classification of Diseases for Oncology, Third Edition* [ICD-O3] codes 20-23, 28-50, 58-69),^26^ squamous cell origin (ICD-O3 8050-8084). Numbers may not equal 100 due to rounding. Twenty-eight cases from patients aged 85-94 years were not used in the estimation of oral cancer death but were used in the estimation of other causes of death (22 364 evaluable cases for oral cancer death).

### Estimates of the Risk of Death From Other Causes for Patients With Oral Cancer

**Figure 2.**
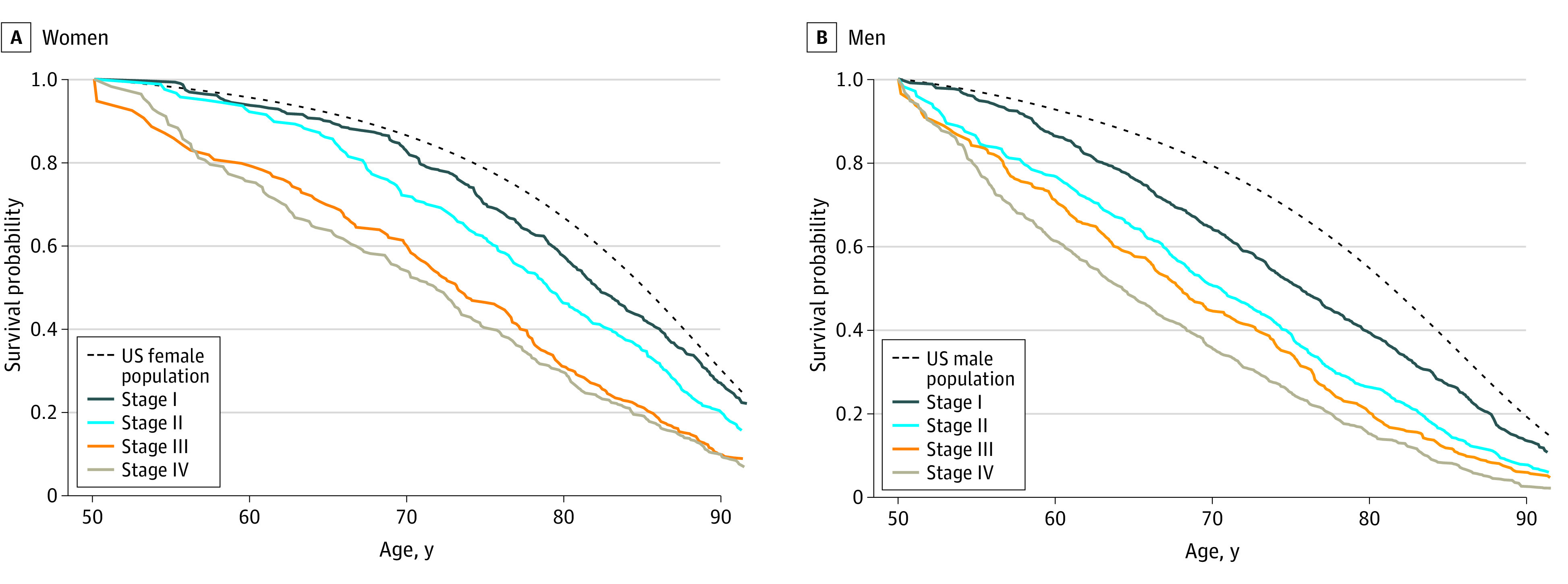
Estimated Noncancer Survival Probability Among Patients With Oral Cancer Survival of patients with oral cancer given their own characteristics but as though they did not have oral cancer, compared with US 2011 life table estimates for female (panel A) and male (panel B) patients with oral cancer, conditional on surviving to age 50 years. Oral cancer patient data are from the Surveillance, Epidemiology, and End Results (SEER) Program, 2000-2011.

We estimated the risk of death from other causes for patients with oral cancer by creating survival curves in which death from causes other than oral cancer was the event of interest, censoring those who died of oral cancer, and conditional on surviving to 50 years of age using left-truncated models computed on the age scale. The resulting curves represent noncancer cause-of-death life tables for persons diagnosed with oral cancer.^28^ We then compared them with the gender-specific US life tables for 2011. Figure 2 shows the estimated likelihood of noncancer survival of patients with oral cancer in the cohort by sex and stage, conditional on being alive at age 50 years. Noncancer survival is the survival estimate given the cohort’s characteristics, but as though they did not have cancer. People with oral cancer have greater competing risks of death (worse noncancer survival) at each stage than the general population, and their noncancer survival worsens by stage. For example, conditional on having survived to age 50 year, a female and male patient diagnosed with stage III cancer would have a 60% and 44% chance, respectively, of being alive at age 70 years, in the absence of their cancer. In the general US population, the corresponding estimates are 86% and 79%, respectively, an absolute difference of 26 and 35 percentage points, respectively.

### Model Results and Validation

At all disease stages, patients with only clinical staging information rather than pathologic staging had a higher risk of death due to oral cancer. Histologic grade was also found to be important in modeling cancer death, with the risk increasing in a stepwise fashion by grade, although the association of grade with death diminished with each increasing stage of disease.^29^ Among patients aged 20 to 65 years with AJCC 6 stage I or II cancer, the adjusted risk of death was 2.7 times greater (95% CI, 1.72-4.11) if the tumor is poorly differentiated or undifferentiated than if the tumor is well differentiated. Among patients aged 66 to 94 years, the risk of death was 3.0 (95% CI, 2.02-4.54) times greater. Depending on the stage of disease, non-Hispanic Blacks and/or Hispanics patients had an increased risk of cancer death compared with non-Hispanic Whites and non-Hispanic Asian and Pacific Islander patients, with the association of race and ethnicity with death due to oral cancer diminishing slightly for higher stages. For example, for a 70-year old male patient with diabetes, who is not married, has stage I disease, surgically assessed primary tumor and nodes, and a poorly differentiated histologic grade, the cumulative 3-year chance of dying from oral cancer is 11.1% (95% CI 8.5%-14.1%) if he is non-Hispanic White, 12.7% (95% CI, 9.0%-17.2%) for if he is non-Hispanic Asian and Pacific Islander, 14.5% (95% CI, 10.0%-19.7%) if he is non-Hispanic Black, and 15.1% (95% CI, 10.4%-20.6%) if he is Hispanic. For the models for causes of death other than oral cancer, the baseline parameters as a function of age provide a life table that forms the baseline for all other patient covariate profiles. The risk of death from causes other than oral cancer increased as a function of stage in both the basic and coexisting conditions models. For example, in the basic model male patients had a relative risk of death from other causes of 1.26 (95% CI, 1.13-1.41) for stage II, 1.35 (95% CI, 1.19-1.54) for stage III, and 1.52 (95% CI, 1.36-1.69) for stage IV, all as compared with stage I. After adjusting for coexisting conditions, the association of stage was somewhat diminished at higher stages, but socioeconomic status of the census tract of residence was significant in both of these models. For example, in the basic model for men, the risk of death due to other causes was 1.53 (95% CI, 1.33-1.75) in the lowest quintile, 1.37 (95% CI, 1.19-1.57) in the second quintile, 1.26 (95% CI, 1.09-1.45) in the third quintile, and 1.22 (95% CI, 1.06-1.40) in the fourth quintile, all as compared with the highest quintile. In the general health assessments models, both smoking status and self-assessed overall health status were statistically significant. Considering that risk factors, treatment patterns, and diagnostic precision or care for other conditions may affect survival probability, we analyzed the periods 2008 to 2011 and 2012 to 2017 separately as well as by each year based on time since diagnosis A comparison of SEER cause-specific survival for oral cancers diagnosed from 2012 to 2017 compared with 2008 to 2011 only showed an improvement of 1.6 percentage points at 5 years (63.4% vs 65.0%) and minimal changes in mortality over time, reflecting secular trends in tobacco use that affect the population at risk of getting and dying of oral cancer (eAppendix 7 and eFigures 25 and 26 in Supplement 1). More details on model estimates can be found in eAppendix 4, eTables 9-18, and eFigure 2 and on baseline hazards in eAppendix 5 and eFigures 3-8 in Supplement 1.

Predicted calibration curves were very well matched to the observed curves, except for some moderate underestimation of death from other causes for those in the most favorable quartiles for the basic and coexisting condition models. The area under the curve (AUC) for the basic and coexisting condition models ranged from 0.76 to 0.83 for oral cancer and from 0.63 to 0.71 for other causes of death, while the AUC for all causes of death from the general health self-assessment model ranged from 0.840 to 0.814 for men and women. When estimating the AUCs for stage- or age-specific groups, they were generally smaller because AUCs are typically larger in more heterogeneous populations due to it being easier to discriminate survival times among more heterogeneous populations. More details on model validation can be found in eFigures 9-24 and eTables 19-21 in Supplement 1.

## Discussion

The SEER OCSC is a new publicly available oral cancer survival calculator designed for people newly diagnosed with oral cancer, their clinicians, and others. This calculator is unique in treating the risk of death from other causes on equal footing with the consideration of the probability of death from the cancer. Users of the system are provided with an estimate of the person’s health status–adjusted age, which the system uses to provide estimates of the likelihood that a person will survive, die of their cancer, or die of other causes. These personalized estimates provide context for discussions about the place of cancer treatment in the affected person’s life as a whole. The system outputs are available in several different visual formats and can be printed out or saved. Particular values of the system are that (1) it provides numerical estimates that are representative of individuals in the US, (2) the estimates reflect that there are competing risks of death operating simultaneously, and (3) the estimated health status–adjusted age and the estimated life expectancy (in years) in the absence of cancer are modifiable.

Prognosis information is important to patients faced with a new cancer diagnosis.^30^ The SEER OCSC also provides a health status–adjusted age and life expectancy in the absence of cancer, which is useful for patients and gives numerical values for the internal gestalt that clinicians often incorporate into treatment discussions. The calculated life expectancy reflects the effect of competing conditions on the person’s life trajectory both within the context of the cancer treatment and outside of it. This system and the novel methodology^14^ that underpins it have potential for a range of applications in cancer registry–based data. Cancer registries often have limited variables describing the likelihood of death from causes other than cancer, and this methodology allows registry data to be combined with other independent or partially overlapping data sources, presenting new opportunities in the future for expansion and use in other cancers.

Our models estimated that people with oral cancer have worse other-cause survival than the general population—they have a higher risk of death from noncancer causes than their age- and sex-matched counterparts, and this is reflected in the calculator results. Even at localized stages, patients with oral cancer have a higher risk of death from other causes than the US population. As with other cancers, the risk of death from other causes rises at advanced cancer stages. One potential hypothesis to explain this is that people diagnosed with advanced-stage cancers tend to also have more comorbid ailments. However, only dementia and diabetes increased by stage at oral cancer diagnosis (eTable 3 in Supplement 1). Therefore, an alternative possibility is that persons diagnosed with advanced-stage oral cancer have other conditions that are undiagnosed, increasing their risk of death from other causes. This might be the case if, for example, those diagnosed with late-stage oral cancer did not act (for whatever reason) on the symptoms of both the growing cancer and other conditions. A third possibility is that having cancer of an advanced stage increases the risk of death not only from cancer but from other causes as well—because of the additional burden of the cancer on the person’s physiology. Whichever hypothesis may be correct, these are important findings with ramifications for individual decision-making, pointing to the potential utility of the new calculator.

Seven other prognostic calculators are available to use for oral cancer and may provide complementary information to this tool. Five predict survival at different time points after diagnosis,^31,32,33,34,35^ and 2 predict survival based on treatment (eTable 22 in Supplement 1).^5,36^ Systematic reviews of the tools suggest they would benefit from wider dissemination, and while the tools are well calibrated, they have modest discriminatory ability, suggesting that the ability to develop more robust models such as the SEER OCSC will be beneficial to the field in the future.^25,37^

### Strengths and Limitations

The SEER OCSC has several strengths. The cancer data in the model come from a population-based sample of 22 392 people in the US. Because the data are population based, estimates are representative of what a typical person is likely to experience with their cancer, rather than reflecting the outcomes of a particular institution, case series, or randomized trial cohort. The SEER OCSC includes alternative and complementary data sources such as the NHIS, which is a strength because it provides data on smoking and self-assessed general health status, as well as Medicare data, which include records of claims for health care, allowing estimates of illness type and severity. Socioeconomic status is incorporated into the system by including information about the person’s area of residence (if they choose to provide it). The inclusion of detailed information about coexisting conditions in the prognosis calculations is particularly useful in oral cancer because alcohol intake and tobacco use, which are commonly associated with the development of these cancers, result in other medical problems that can adversely affect survival even in the absence of cancer, such as heart disease, chronic obstructive pulmonary disease, and liver disease.^4^

The SEER OCSC, like all such tools, has limitations. The coexisting conditions data come from Medicare files, so the information for the models is directly available only for those 66 years of age or older. Some important prognostic variables, such as cancer margin status, data on extracapsular tumor spread, and depth of tumor invasion, and intangible but important factors such as levels of family and social support are not available in registry or other population-based data sources. The cancer staging data are from AJCC 6. Cancer-specific cumulative mortality estimates provided by the SEER OCSC may be slightly higher than they would be if the data were assessed using the eighth edition of the AJCC. There is not yet sufficient follow-up time available to use AJCC 8, which just came into use in 2018. Additionally, there are variables that we have chosen not to include, such as pack-years of smoking and alcohol intake. Lastly, death certificate data are inherently imperfect. Methods have been devised to overcome these problems,^15^ but misclassification can result in over- or underestimation of cancer deaths.

## Conclusions

The publicly available SEER OCSC uses novel approaches to modeling competing risks of death: the risk of death from other causes is treated on equal footing with the consideration of the probability of death from the cancer. It provides personalized estimates of health status–adjusted age, life expectancy in the absence of the cancer, and probability of surviving, dying from the cancer, or dying from other causes 1 to 10 years after diagnosis. A novel extension of existing methodology using registry data, the approach will be broadly applicable for developing prognostic models that can capture the cancer and noncancer aspects of a person’s health. As registries develop more linkages, available covariates will become broader, making future tools even more clinically relevant. The models developed for the SEER OCSC demonstrate that survival estimates that exclude the effects of coexisting conditions can lead to under- or overestimates of survival. In the case of oral cancer, the models and this calculator show that many patients with oral cancer have a greater risk of dying of other causes due to the number and type of coexisting conditions they have. Furthermore, their likelihood of dying of other causes increases as their cancer stage increases, even after adjusting for coexisting conditions.

## Notes

Supplement 1. **eAppendix 1.** Data Sources **eTable 1.** Number of Patients 20-65 Years Old Over Selected Variables Included in the Analysis **eTable 2.** Number of Patients 66-94 Years Old at Diagnosis That Were Included in the Analysis **eTable 3.** Major Comorbidities Among Oral Cancer Patients Age 66 and Older **eTable 4.** Characteristics of Study Population: US Adult Respondents, Age 35 Years and Older at the Time of the Interview, National Health Interview Survey, 1986-2009, With Mortality Follow-Up Through 2011
eMethods.
**eTable 5.** Applicable Ages at Diagnosis for Each of the Four Non-Oral Cancer Cause of Death Models **eFigure 1.** The Flow Through the Calculator Interface to Facilitate Patient Use **eAppendix 2.** Other Causes of Death Submodels **eAppendix 3.** Model Estimation **eTable 6.** Examples of Single and Double Coding of Patient Records for the Basic Model **eTable 7.** Examples of Single and Double Coding of Patient Records for the Coexisting Condition Model **eTable 8.** Example of Single Coding of Patient Records for General Health Self-Assessment Model **eAppendix 4.** Model Estimates **eTable 9.** Probabilities of Death and Hazard Ratios of Dying for Oral Cancer Cause of Death Submodel for Stage I **eTable 10.** Probabilities of Death and Hazard Ratios of Dying for Oral Cancer Cause of Death Submodel for Stage II **eTable 11.** Probabilities of Death and Hazard Ratios of Dying for Oral Cancer Cause of Death Submodel for Stage III **eTable 12.** Probabilities of Death and Hazard Ratios of Dying for Oral Cancer Cause of Death Submodel for Stage IV **eFigure 2.** Cancer Death Submodel Baseline Survival by Stage **eTable 13.** Probabilities of Death and Hazard Ratios of Dying for the Other Cause of Death Submodel in the Basic Model (Males) **eTable 14.** Probabilities of Death and Hazard Ratios of Dying for the Other Cause of Death Submodel in the Basic Model (Females) **eTable 15.** Probabilities of Death and Hazard Ratios of Dying for the Other Cause of Death Submodel in the Coexisting Condition Model (Males) **eTable 16.** Probabilities of Death and Hazard Ratios of Dying for the Other Cause of Death Submodel in the Coexisting Condition Model (Females) **eTable 17.** Probabilities of Death and Hazard Ratios of Dying for the Other Cause of Death Submodel in the General Health Self-Assessment Model (Males) **eTable 18.** Probabilities of Death and Hazard Ratios of Dying for the Other Cause of Death Submodel in the General Health Self-Assessment Model (Females) **eAppendix 5.** Baseline Hazards for the Other Cause of Death Submodels **eFigure 3.** Fit of Gompertz Model to Baseline Hazards for Other Cause of Death Submodel for Coexisting Condition Model (Males) **eFigure 4.** Fit of Gompertz model to Baseline Hazards for Other Cause of Death Submodel for Coexisting Condition Model (Females) **eFigure 5.** Fit of Gompertz Model to Baseline Hazards for Other Cause of Death Submodel (for General Health Self-Assessment Model (Males) **eFigure 6.** Fit of Gompertz Model to Baseline Hazards for Other Cause of Death Submodel for General Health Self-Assessment Model (Females) **eFigure 7.** Baseline Hazards of Death From Other Causes by Age for the Other Cause of Death Submodel for Basic Model (Males) **eFigure 8.** Baseline Hazards of Death From Other Causes by Age for the Other Cause of Death Submodel for Basic Model (Females) **eAppendix 6.** Validation **eFigure 9.** Validation of Basic Model for ages 20-86, All Stages, Based on Observed Versus Predicted Cumulative Mortality Curves for the Cancer Death Submodel and Other Cause of Death Submodel **eFigure 10.** Validation of Basic Model for Ages 20-86, Stage I, Based on Observed Versus Predicted Cumulative Mortality Curves for the Cancer Death Submodel and Other Cause of Death Submodel **eFigure 11.** Validation of Basic Model for Ages 20-86, Stage II, Based on Observed Versus Predicted Cumulative Mortality Curves for the Cancer Death Submodel and Other Cause of Death Submodel **eFigure 12.** Validation of Basic Model for Ages 20-86, Stage III, Based on Observed Versus Predicted Cumulative Mortality Curves for the Cancer Death Submodel and Other Cause of Death Submodel **eFigure 13.** Validation of Basic Model for Ages 20-86, Stage IV, Based on Observed Versus Predicted Cumulative Mortality Curves for the Cancer Death Submodel and Other Cause of Death Submodel **eFigure 14.** Validation of Basic Model for Ages 20-86, Males, Based on Observed Versus Predicted Cumulative Mortality Curves for the Cancer Death Submodel and Other Cause of Death Submodel **eFigure 15.** Validation of Basic Model for Ages 20-86, Females, Based on Observed Versus Predicted Cumulative Mortality Curves for the Cancer Death Submodel and Other Cause of Death Submodel **eTable 19.** Area Under the Curve (AUC) Statistics for the Cancer Death Submodel (AUC1) and the Other Cause of Death Submodel (AUC2) of the Basic Model (Ages 20-86) by Stage and Sex **eFigure 16.** Validation of Coexisting Condition Model for Ages 66-86, All Stages, Based on Observed Versus Predicted Cumulative Mortality Curves for the Cancer Death Submodel and Other Cause of Death Submodel **eFigure 17.** Validation of Coexisting Condition Model for Ages 66-86, Stage I, Based on Observed Versus Predicted Cumulative Mortality Curves for the Cancer Death Submodel and Other Cause of Death Submodel **eFigure 18.** Validation of Coexisting Condition Model for Ages 66-86, Stage II, Based on Observed Versus Predicted Cumulative Mortality Curves for the Cancer Death Submodel and Other Cause of Death Submodel **eFigure 19.** Validation of Coexisting Condition Model for Ages 66-86, Stage III, Based on Observed Versus Predicted Cumulative Mortality Curves for the Cancer Death Submodel and Other Cause of Death Submodel **eFigure 20.** Validation of Coexisting Condition Model for Ages 66-86, Stage IV, Based on Observed Versus Predicted Cumulative Mortality Curves for the Cancer Death Submodel and Other Cause of Death Submodel **eFigure 21.** Validation of Coexisting Condition Model for Ages 66-86, Males, Based on Observed Versus Predicted Cumulative Mortality Curves for the Cancer Death Submodel and Other Cause of Death Submodel **eFigure 22.** Validation of Coexisting Condition Model for Ages 66-86, Females, Based on Observed Versus Predicted Cumulative Mortality Curves for the Cancer Death Submodel and Other Cause of Death Submodel **eTable 20.** Area Under the Curve (AUC) Statistics for the Cancer Death Submodel (AUC1) and the Other Cause of Death Submodel (AUC2) of the Coexisting Condition Model (Ages 66-86) by Stage and Sex **eFigure 23.** Ten-Fold Cross-Validation Based on Observed Versus Predicted Cumulative Mortality Curves for Males for All Causes of Death (in This Case Taken to Represent Other Causes of Death) Based on the Other Cause of Death Submodel for Time From Interview **eFigure 24.** Ten-Fold Cross-Validation Based on Observed Versus Predicted Cumulative Mortality Curves for Females for All Causes of Death (in This Case Taken to Represent Other Causes of Death) Based on the Other Cause Mortality Submodel for Time From Interview **eTable 21.** Area Under the Curve (AUC) Statistics for the Other Cause of Death Submodel of the General Health Self-Assessment Model (NHIS Data Ages 40-86) by Age and Sex **eTable 22.** Position of the SEER OCSC in Relation to Other Available Oral Cancer Calculators
eReferences.

Supplement 2.
Data Sharing Statement
