# Supplementary material for: A New Personalized Oral Cancer Survival Calculator to Estimate Risk of Death From Both Oral Cancer and Other Causes
Source: JAMA Otolaryngol Head Neck Surg. 2023 Jul 10;149(11):993–1000. doi: 10.1001/jamaoto.2023.1975 (PMC10334297; doi:10.1001/jamaoto.2023.1975)
Supplement: Supplement 1. — eAppendix 1. Data Sources eTable 1. Number of Patients 20-65 Years Old Over Selected Variables Included in the Analysis eTable 2. Number of Patients 66-94 Years Old at Diagnosis That Were Included in the Analysis eTable 3. Major Comorbidities Among Oral Cancer Patients Age 66 and Older eTable 4. Characteristics of Study Population: US Adult Respondents, Age 35 Years and Older at the Time of the Interview, National Health Interview Survey, 1986-2009, With Mortality Follow-Up Through 2011 eMethods. eTable 5. Applicable Ages at Diagnosis for Each of the Four Non-Oral Cancer Cause of Death Models eFigure 1. The Flow Through the Calculator Interface to Facilitate Patient Use eAppendix 2. Other Causes of Death Submodels eAppendix 3. Model Estimation eTable 6. Examples of Single and Double Coding of Patient Records for the Basic Model eTable 7. Examples of Single and Double Coding of Patient Records for the Coexisting Condition Model eTable 8. Example of Single Coding of Patient Records for General Health Self-Assessment Model eAppendix 4. Model Estimates eTable 9. Probabilities of Death and Hazard Ratios of Dying for Oral Cancer Cause of Death Submodel for Stage I eTable 10. Probabilities of Death and Hazard Ratios of Dying for Oral Cancer Cause of Death Submodel for Stage II eTable 11. Probabilities of Death and Hazard Ratios of Dying for Oral Cancer Cause of Death Submodel for Stage III eTable 12. Probabilities of Death and Hazard Ratios of Dying for Oral Cancer Cause of Death Submodel for Stage IV eFigure 2. Cancer Death Submodel Baseline Survival by Stage eTable 13. Probabilities of Death and Hazard Ratios of Dying for the Other Cause of Death Submodel in the Basic Model (Males) eTable 14. Probabilities of Death and Hazard Ratios of Dying for the Other Cause of Death Submodel in the Basic Model (Females) eTable 15. Probabilities of Death and Hazard Ratios of Dying for the Other Cause of Death Submodel in the Coexisting Condition Model (Males) eTable 16. Probabilities [file jamaotolaryngolheadnecksurg-e231975-s001.pdf]

Supplemental Online Content

Davies L, Hankey BF, Wang Z, et al. A new personalized oral cancer survival calculator to estimate risk of death from both oral cancer and other causes. *JAMA Otolaryngol Head Neck Surg.* Published online July 10, 2023. doi:10.1001/jamaoto.2023.1975

eAppendix 1. Data Sources

eTable 1. Number of Patients 20-65 Years Old Over Selected Variables Included in the Analysis

eTable 2. Number of Patients 66-94 Years Old at Diagnosis That Were Included in the Analysis

eTable 3. Major Comorbidities Among Oral Cancer Patients Age 66 and Older

eTable 4. Characteristics of Study Population: US Adult Respondents, Age 35 Years and Older at the Time of the Interview, National Health Interview Survey, 1986-2009, With Mortality Follow-Up Through 2011

eMethods.

eTable 5. Applicable Ages at Diagnosis for Each of the Four Non-Oral Cancer Cause of Death Models

eFigure 1. The Flow Through the Calculator Interface to Facilitate Patient Use

eAppendix 2. Other Causes of Death Submodels

eAppendix 3. Model Estimation

eTable 6. Examples of Single and Double Coding of Patient Records for the Basic Model

eTable 7. Examples of Single and Double Coding of Patient Records for the Coexisting Condition Model

eTable 8. Example of Single Coding of Patient Records for General Health Self-Assessment Model

eAppendix 4. Model Estimates

eTable 9. Probabilities of Death and Hazard Ratios of Dying for Oral Cancer Cause of Death Submodel for Stage I

eTable 10. Probabilities of Death and Hazard Ratios of Dying for Oral Cancer Cause of Death Submodel for Stage II

eTable 11. Probabilities of Death and Hazard Ratios of Dying for Oral Cancer Cause of Death Submodel for Stage III

© 2023 Davies L et al. *JAMA Otolaryngology–Head & Neck Surgery.*

eTable 12. Probabilities of Death and Hazard Ratios of Dying for Oral Cancer Cause of Death Submodel for Stage IV

eFigure 2. Cancer Death Submodel Baseline Survival by Stage

eTable 13. Probabilities of Death and Hazard Ratios of Dying for the Other Cause of Death Submodel in the Basic Model (Males)

eTable 14. Probabilities of Death and Hazard Ratios of Dying for the Other Cause of Death Submodel in the Basic Model (Females)

eTable 15. Probabilities of Death and Hazard Ratios of Dying for the Other Cause of Death Submodel in the Coexisting Condition Model (Males)

eTable 16. Probabilities of Death and Hazard Ratios of Dying for the Other Cause of Death Submodel in the Coexisting Condition Model (Females)

eTable 17. Probabilities of Death and Hazard Ratios of Dying for the Other Cause of Death Submodel in the General Health Self-Assessment Model (Males)

eTable 18. Probabilities of Death and Hazard Ratios of Dying for the Other Cause of Death Submodel in the General Health Self-Assessment Model (Females)

eAppendix 5. Baseline Hazards for the Other Cause of Death Submodels

eFigure 3. Fit of Gompertz Model to Baseline Hazards for Other Cause of Death Submodel for Coexisting Condition Model (Males)

eFigure 4. Fit of Gompertz model to Baseline Hazards for Other Cause of Death Submodel for Coexisting Condition Model (Females)

eFigure 5. Fit of Gompertz Model to Baseline Hazards for Other Cause of Death Submodel (for General Health Self-Assessment Model (Males)

eFigure 6. Fit of Gompertz Model to Baseline Hazards for Other Cause of Death Submodel for General Health Self-Assessment Model (Females)

eFigure 7. Baseline Hazards of Death From Other Causes by Age for the Other Cause of Death Submodel for Basic Model (Males)

eFigure 8. Baseline Hazards of Death From Other Causes by Age for the Other Cause of Death Submodel for Basic Model (Females)

eAppendix 6. Validation

eFigure 9. Validation of Basic Model for ages 20-86, All Stages, Based on Observed Versus Predicted Cumulative Mortality Curves for the Cancer Death Submodel and Other Cause of Death Submodel

eFigure 10. Validation of Basic Model for Ages 20-86, Stage I, Based on Observed Versus Predicted Cumulative Mortality Curves for the Cancer Death Submodel and Other Cause of Death Submodel

eFigure 11. Validation of Basic Model for Ages 20-86, Stage II, Based on Observed Versus Predicted Cumulative Mortality Curves for the Cancer Death Submodel and Other Cause of Death Submodel

eFigure 12. Validation of Basic Model for Ages 20-86, Stage III, Based on Observed Versus Predicted Cumulative Mortality Curves for the Cancer Death Submodel and Other Cause of Death Submodel

eFigure 13. Validation of Basic Model for Ages 20-86, Stage IV, Based on Observed Versus Predicted Cumulative Mortality Curves for the Cancer Death Submodel and Other Cause of Death Submodel

eFigure 14. Validation of Basic Model for Ages 20-86, Males, Based on Observed Versus Predicted Cumulative Mortality Curves for the Cancer Death Submodel and Other Cause of Death Submodel

eFigure 15. Validation of Basic Model for Ages 20-86, Females, Based on Observed Versus Predicted Cumulative Mortality Curves for the Cancer Death Submodel and Other Cause of Death Submodel

eTable 19. Area Under the Curve (AUC) Statistics for the Cancer Death Submodel (AUC1) and the Other Cause of Death Submodel (AUC2) of the Basic Model (Ages 20-86) by Stage and Sex

eFigure 16. Validation of Coexisting Condition Model for Ages 66-86, All Stages, Based on Observed Versus Predicted Cumulative Mortality Curves for the Cancer Death Submodel and Other Cause of Death Submodel

eFigure 17. Validation of Coexisting Condition Model for Ages 66-86, Stage I, Based on Observed Versus Predicted Cumulative Mortality Curves for the Cancer Death Submodel and Other Cause of Death Submodel

eFigure 18. Validation of Coexisting Condition Model for Ages 66-86, Stage II, Based on Observed Versus Predicted Cumulative Mortality Curves for the Cancer Death Submodel and Other Cause of Death Submodel

eFigure 19. Validation of Coexisting Condition Model for Ages 66-86, Stage III, Based on Observed Versus Predicted Cumulative Mortality Curves for the Cancer Death Submodel and Other Cause of Death Submodel

eFigure 20. Validation of Coexisting Condition Model for Ages 66-86, Stage IV, Based on Observed Versus Predicted Cumulative Mortality Curves for the Cancer Death Submodel and Other Cause of Death Submodel

eFigure 21. Validation of Coexisting Condition Model for Ages 66-86, Males, Based on Observed Versus Predicted Cumulative Mortality Curves for the Cancer Death Submodel and Other Cause of Death Submodel

© 2023 Davies L et al. *JAMA Otolaryngology–Head & Neck Surgery.*

eFigure 22. Validation of Coexisting Condition Model for Ages 66-86, Females, Based on Observed Versus Predicted Cumulative Mortality Curves for the Cancer Death Submodel and Other Cause of Death Submodel

eTable 20. Area Under the Curve (AUC) Statistics for the Cancer Death Submodel (AUC1) and the Other Cause of Death Submodel (AUC2) of the Coexisting Condition Model (Ages 66-86) by Stage and Sex

eFigure 23. Ten-Fold Cross-Validation Based on Observed Versus Predicted Cumulative Mortality Curves for Males for All Causes of Death (in This Case Taken to Represent Other Causes of Death) Based on the Other Cause of Death Submodel for Time From Interview

eFigure 24. Ten-Fold Cross-Validation Based on Observed Versus Predicted Cumulative Mortality Curves for Females for All Causes of Death (in This Case Taken to Represent Other Causes of Death) Based on the Other Cause Mortality Submodel for Time From Interview

eTable 21. Area Under the Curve (AUC) Statistics for the Other Cause of Death Submodel of the General Health Self-Assessment Model (NHIS Data Ages 40-86) by Age and Sex

eTable 22. Position of the SEER OCSC in Relation to Other Available Oral Cancer Calculators

eAppendix 7. Applicability of Mortality and Survival Data Over Time

eFigure 25. Comparison of Survival Curves 2008-2011 vs 2012-2017

eFigure 26. Survival by Year of Diagnosis

eReferences.

This supplemental material has been provided by the authors to give readers additional information about their work.

eAppendix 1. Data Sources
eS1.1 SEER and SEER-Medicare Linked Data

The data sources utilized were the Surveillance, Epidemiology, and End Results (SEER) Program based at the National Cancer Institute (NCI) and Medicare data from the Center for Medicare & Medicaid Services (CMS). The SEER Program of the National Cancer Institute (NCI) is an authoritative source of information on cancer incidence and survival in the United States. The SEER Program currently collects and publishes cancer incidence and survival data from population-based cancer registries. For this analysis we utilized the designated SEER 18 registry areas covering approximately 34.6 percent of the U.S. population which includes substantial percentages of the various racial/ethnic groups included in the US population.

The SEER registries routinely collect data on patient demographics, primary tumor site, tumor morphology and stage at diagnosis, first course of treatment, and follow-up for vital status. The SEER data have been used by thousands of researchers, clinicians, public health officials, legislators, policymakers, community groups, and the public to address research and cancer control issues.

The CMS manages Medicare and a number of other health care related services for the country. Medicare claims are linked biannually to SEER data. Comorbidity score was calculated using the NCI comorbidity index algorithm (https://healthcaredelivery.cancer.gov/seermedicare/considerations/comorbidity.html) for cancer patients 66+ years old at diagnosis.

For this study, claims are available for those who have continuous Part A and Part B coverage and did not receive their care through a Health Maintenance Organization (HMO). The selection and exclusion criteria for obtaining the patient data from the SEER database used in the survival analysis for the Oral Cancer Survival Calculator (OCSC) are listed below. It should be noted that the Human Papilloma Virus is a risk factor for some head and neck cancers, but it is not known to be an important risk factor (or prognostic factor) for the cancers included in this project.

Select

- Data file: SEER 18 Regs (Excl AK) Custom Data, SEER CSC, Nov 2014 Sub (2000-2011)
- Malignant behavior
- Sequence Number = 0/1
- Diagnosis years 2000-2011
- ICD-O-3 = 8050-8084
- Restrict to Oral Cavity: ICD-03 topography codes: 20-23, 28-50, 58-59, 60-69
- Registries: All SEER minus American Indians and Alaska Natives
- Actively Followed
- Male or Female
- Ages 20-94
- Cases in Research Database
- Study cut-off: 12/2012
- Cause of death (Howlader definition)[1]

Exclude

- Death certificate or autopsy only
- Alive/censored with zero survival time
- Unknown cause of death

© 2023 Davies L et al. *JAMA Otolaryngology–Head & Neck Surgery.*

Study Variables	Definition	Comments
Race	White Non hispanic, Black Non hispanic, API Non-hispanic, Hispanic	SEER variable.
Age at Diagnosis	Restricted Cubic Spline of age in years	SEER variable. Used knots at 5, 35, 65, and 95 percentiles.
Gender	Male, Female	SEER variable.
Diagnosis Year	2000-2011	SEER variable; Grouped as 2000-03, 2004-07, 2008-11
Stage[2]	I, II, III, IVA, IVB, IVC, IV(NOS)	SEER variable; AJCC Stage (6th Edition)
Grade	Well Differentiated<br>Moderately Differentiated<br>Poorly/Undifferentiated	SEER variable.
Marital Status	Married (including common law), Not Married	SEER variable.
Socioeconomic Status	Quintiles based on Yost index for oral cancer patients.	See below for a description of how this variable was constructed
Clinical vs Pathological Staging – 6th Edition of AJCC Staging Manual	Path T Path N (SSS=20-90, NNP=0-94,97)<br>Clin T Path N (SSS=0-19, NNP=0-94,97)<br>Path T Clin N (SSS=20-90, NNP=95,98)<br>Clin T Clin N (SSS=0-19, NNP=95,98)	SEER Variables:<br>SSS = Site Specific Surgery<br>NNP = Number of Nodes Positive.<br>See below for a description of the motivation for including this variable
Comorbidity Score	Restricted Cubic Spline of comorbidity score	Although all cases were included, knots were calculated at the 5th, 35th, 65th, and 95th percentiles among those who have at least one comorbidity (i.e non-zero scores). Obtained from linkage to Medicare data. Only available for ages 66-94. See below for description of how this variable was created.
Cause Of Death	Howlader definition[1]	Death from cancer is event of interest; deaths from other causes, patients lost to follow-up, or patients alive at end of the study period are censored observations.

[1]Howlader N, Ries LA, Mariotto AB, Reichman ME, Ruhl J, Cronin KA. Improved estimates of cancer-specific survival rates from population-based data. J Natl Cancer Inst. 2010 Oct 20;102(20):1584-98.

The socioeconomic status variable used in the modeling was based on a study by Yu et al[1]. One of the two indices they developed was based on a previous study by Yost et al[2]. Yu et al[1] used the same variables identified by Yost et al[2] as being relevant measures of SES in constructing an area-based measure at the census tract level using SEER data, ecologic data from the Census, and factor analysis. This index was used to classify oral cancer patients into quintiles which was the SES measure used in the modeling for the oral cancer CSC. The Comorbidity Score was obtained from a study by Mariotto et al[3] where SEER data were linked to Medicare data and a regression model of other cause survival was developed that included patient comorbidities present at the diagnosis of their cancer. In that study, ICD-9-CM codes and Current Procedure Terminology (CPT) codes recorded in claims made during the 12 months prior to a cancer diagnosis were used to identify 15 comorbid conditions based on Charlson et al[4].

© 2023 Davies L et al. *JAMA Otolaryngology–Head & Neck Surgery.*

Excluded were diagnostic codes corresponding to solid tumors and lymphoma/leukemia because of the focus on studying non-cancer comorbidity and other cause mortality in cancer patients. The 15 comorbid conditions in Mariotto et al[3] were: acute myocardial infarction, AIDS, cerebrovascular disease, chronic renal failure, congestive heart failure, chronic obstructive pulmonary disease (COPD), dementia, diabetes, cirrhosis and/or chronic hepatitis, moderate/severe liver disease, history of myocardial infarction (MI), paralysis, rheumatologic disease, ulcer disease, and vascular disease. In subsequent unpublished work, Mariotto combined cirrhosis and/or chronic hepatitis, and moderate/severe liver disease into a single liver disease category, reducing the number of conditions to 14, which is what was used here. Conditions reported during the month of diagnosis were excluded to avoid misclassifying complications as comorbid conditions. A rule-out algorithm was also used so that only conditions appearing on more than one physician claim were included, thereby ensuring diagnoses recorded only in Part B claims were not transient episodes. A Cox model that included dummy variables for each of the various comorbid conditions, was used to estimate their effect on the dependent variable non-cancer death. Age, sex and race were included as additional variables in the model to control for their independent effects on non-cancer mortality. There were also interaction terms between selected comorbid conditions to account for correlated effects on survival. In this study, the comorbidity score for a patient was the sum of the regression coefficients from the study by Mariotto et al[3] multiplied by the condition indicator for a patient (1=has the condition; 0=does not have the condition) for a patient's profile of comorbidities, and this score was one of the prognostic variables used to model both cancer and other cause survival.

Comorbidity scores represent the risk of death from other causes, and having multiple comorbidities, even if moderate, can increase the score even more. With respect to its impact on death from causes other than cancer, the continuous version of comorbidity score is sometimes classified into 3 groups: none (zero comorbidity score), low/medium (comorbidity score of 1 to 560) and high (comorbidity score of 561+), based on survival and clinical judgements. Moderate comorbidities include cardiovascular disease; paralysis; diabetes; or combinations of diabetes with MI, ulcer, or rheumatologic disease. Severe comorbidities include AIDS, COPD, mild or severe liver disease, chronic renal failure, dementia, congestive heart failure, or combinations of aforementioned less severe diseases."

eTable 1 includes descriptive statistics for the SEER patients ages 20-65 included in the survival analysis for the Oral Cancer Survival Calculator (OCSC). Variables that were unknown were imputed using the R package Multivariate Imputation by Chained Equations (MICE) which provided for specifying the type of model used to do the imputation for a given variable using all available SEER variables and the estimated cumulative net mortality hazards for both oral cancer and other cause of death.

eTable 2 includes descriptive statistics for the SEER-Medicare patients 66-94 years of age at diagnosis that were included in the study. Since the comorbidity score was determined based on claims for the year prior to diagnosis, the minimum age at diagnosis for which a pre-diagnosis comorbidity score could be developed was age 66. The SEER data linked to Medicare data used here had a study cut-off date for patient follow-up of December 31, 2011. For patients who did not have a comorbidity score, it was imputed using the same MICE algorithm as mentioned above.

eTable 3 presents the percent of patients with specified comorbidities by stage. No association with stage was found by level of comorbidity (i.e., No Comorbidities, Low/Medium Comorbidities, or High Comorbidities). For individual comorbidities, there was evidence of an association with stage for the percentage of patients with Dementia with an increasing trend by stage. There is also some evidence of an increasing trend by stage in the percentages of patients with diabetes, and patients with liver disease.

© 2023 Davies L et al. *JAMA Otolaryngology–Head & Neck Surgery.*

eTable 1. Number of patients 20-65 years old over selected variables included in the analysis. Ages 20-65 (12,862 patients)

Race/Ethnicity	Known	Unknown (Imputed)	Total
Hispanic*	971 (7.64)	0*	971 (7.55)
NH White	9,584 (75.38)	112 (76.19)	9,696 (75.38)
NH Black	1,304 (10.26)	19 (12.93)	1,323 (10.29)
NH API	856 (6.73)	16 (10.88)	872 (6.78)
Totals	12,715 (100.00)	147 (100.00)	12,862 (100.00)
**Marital Status**	**Known**	**Unknown (Imputed)**	**Total**
Not Married	5,340 (45.03)	448 (44.67)	5,788 (45.00)
Married	6,519 (54.97)	555 (55.33)	7,074 (55.00)
Totals	11,859 (100.00)	1,003 (100.00)	12,862 (100.00)
**Path/Clin T/N**	**Known**	**Unknown (Imputed)**	**Total**
Path/Clin T Path N	6,417 (51.26)	183 (53.20)	6,600 (51.31)
Path T Clin N	3,826 (30.56)	93 (27.03)	3,919 (30.47)
Clin T Clin N	2,275 (18.17)	68 (19.77)	2,343 (18.22)
Totals	12,518 (100.00)	344 (100.00)	12,862 (100.00)
**Grade**	**Known**	**Unknown (Imputed)**	**Total**
Grade I	2,589 (23.40)	552 (30.70)	3,141 (24.42)
Grade II	6,398 (57.83)	959 (53.34)	7,357 (57.20)
Grade III or IV	2,077 (18.77)	287 (15.96)	2,364 (18.38)
Totals	11,064 (100.00)	1,798 (100.00)	12,862 (100.00)
**Stage**	**Known**	**Unknown (Imputed)**	**Total**
1	3,739 (35.25)	1,028 (45.59)	4,767 (37.06)
2	1,656 (15.61)	402 (17.83)	2,058 (16.00)
3	1,655 (15.60)	246 (10.91)	1901 (14.78)
4	3,557 (33.53)	579 (25.68)	4136 (32.16)
Totals	10,607 (100.00)	2,255 (100.00)	12,862 (100.00)
**Yost Quintile**	**Known**	**Unknown (Imputed)**	**Total**
1	3,030 (23.92)	54 (27.69)	3,084 (23.98)
2	2,413 (19.05)	37 (18.97)	2,450 (19.05)
3	2,317 (18.29)	35 (17.95)	2,352 (18.29)
4	2,582 (20.38)	34 (17.44)	2,616 (20.34)
5	2,325 (18.35)	35 (17.95)	2,360 (18.35)
Totals	12,667 (100.00)	195 (100.00)	12,862 (100.00)

*Hispanics are identified using the North American Association of Central Cancer Registries Hispanic Identification Algorithm (NHIA) so that there are no Unknowns.

© 2023 Davies L et al. *JAMA Otolaryngology–Head & Neck Surgery.*

eTable 2. Number of patients 66-94 years old at diagnosis that were included in the analysis. Ages 66-94 (9,530 patients) Cells are suppressed that have counts <10 and are indicated as (-).

Race/Ethnicity	Known	Unknown (Imputed)	Total	
Hispanic	618 (6.55)	0*	618 (6.48)	
NH White	7,716 (81.72)	(-)	7,791 (81.75)	
NH Black	488 (5.17)	(-)	495 (5.19)	
NH API	620 (6.57)	(-)	626 (6.57)	
Totals	9,442 (100.00)	88(100.00)		9,530 (100.00)
**Marital Status**	**Known**	**Unknown (Imputed)**	**Total**	
Not Married	4,279 (49.14)	422 (51.34)	4,701 (49.33)	
**Married**	4,429(50.86)	400 (48.66)	4,829 (50.67)	
Totals	8,708 (100.00)	822 (100.00)		9,530 (100.00)
**Path/Clin T/N**	**Known**	**Unknown (Imputed)**	**Total**	
Path/Clin T e Path N	3,436 (37.28)	112 (35.78)		3,548 (37.23)
Path T Clin N	3,439 (37.31)	94 (30.03)		3,533 (37.07)
Clin T Clin N	2,342 (25.41)	107 (34.19)		2,449 (25.70)
Totals	9,217 (100.00)	313 (100.00)		9,530 (100.00)
**Grade**	**Known**	**Unknown (Imputed)**	**Total**	
Grade I	2,290 (28.39)	448 (30.62)	2,738 (28.73)	
Grade II	4,372 (54.20)	772 (52.77)	5,144 (53.98)	
Grade III or IV	1,405 (17.42)	243 (16.61)	1,648 (17.29)	
Totals	8,067 (100.00)	1,463 (100.00)		9,530 (100.00)
**Stage**	**Known**	**Unknown (Imputed)**	**Total**	
1	2,413 (32.51)	757 (35.91)		3,170 (33.26)
2	1,526 (20.56)	465 (22.06)		1,991 (20.89)
3	1,240 (16.71)	279 (13.24)		1,519 (15.94)
4	2,243 (30.22)	607 (28.80)		2,850 (29.91)
Totals	7422 (100.00)	2,108 (100.00)		9,530 (100.00)
**Yost Quintile**	**Known**	**Unknown (Imputed)**	**Total**	
1	1,878 (20.00)	38 (26.76)	1,916 (20.10)	
2	1,878 (20.00)	18 (12.68)	1,896 (19.90)	
3	1,877 (19.99)	27 (19.01)	1,904 (19.98)	
4	1,878 (20.00)	32 (22.54)	1,910 (20.04)	
5	1,877 (19.99)	27 (19.01)	1,904 (19.98)	
Totals	9,388 (100.00)	142 (100.00)		9,530 (100.00)
**Comorbidity Score**	**Known**	**Unknown (Imputed)**	**Total**	
None	3,263 (52.79	1,815 (54.20)		5,078 (53.28)
Low/Medium	1,025 (16.58)	551 (16.45)		1,576 (16.54)
High	1,893 (30.63)	983 (29.35)		2,876 (30.18)
Totals	6,181 (100.00)	3,349 (100.00)		9,530 (100.00)

*Hispanics are identified using the North American Association of Central Cancer Registries Hispanic Identification Algorithm (NHIA) so that there are no Unknowns.

© 2023 Davies L et al. *JAMA Otolaryngology–Head & Neck Surgery.*

eTable 3. Major Comorbidities among oral cancer patients age 66 and older. This table includes only patients that were linked to Medicare. Patients can have more than one comorbidity. Cells are suppressed that have counts <10 and are indicated as (-). The small number of patients in the cohort with AIDS, moderate to severe liver damage and hemi or paraplegia prohibited inclusion in the table, although these comorbidities are included in the SEER OCSC system. Note that having multiple moderate comorbidities can result in classification as "high".

**Comorbidity –**	**Stage I** n=1,561	**II** n=963	**III** n=801	**IV** (n=1,437)	**Unknown** (n=1,419)	**Chi squared test for number of conditions for stages I-IV**[A]	
**No comorbidities**	833 (53.36%)	510 (52.96%)	443 (55.31%)	753 (52.40%)	724 (51.02%)	3.60 (p = 0.73)	
**low/medium comorbidities**	266 (17.04%)	152 (15.78%)	131 (16.35%)	233 (16.21%)	243 (17.12%)		
**high comorbidities**	462 (29.60%)	301 (31.26%)	227 (23.84%)	451 (31.38%)	452 (31.85%)		
						**Chi squared test for difference between stages I-IV**[B]	**Cochran - Armitage ordinal test for trend by stage I-IV**[C]
**Acute MI**	13 (0.83%)	11 (1.14%)	10 (1.25%)	12 (0.84%)	13 (0.92%)	1.53 (P = 0.68)	-0.09 (P = 0.92)
**Congestive Heart Failure**	113 (7.37%)	92 (9.55%)	79 (9.86%)	131 (9.12%)	157 (11.06%)	5.99 (P = 0.11)	-1.77 (P = 0.08)
**COPD**	277 (17.75%)	175 (18.17%)	135 (16.85%)	252 (17.54%)	242 (17.05%)	0.55 (P = 0.91)	0.30 (P = 0.77)
**Cerebrovascular disease**	110 (7.05%)	64 (6.65%)	62 (7.74%)	112 (7.79%)	126 (8.88%)	1.50 (P = 0.68)	-0.93 (P = 0.35)
**Dementia**	28 (1.79%)	31 (3.22%)	29 (3.62%)	77 (5.36%)	81 (5.71%)	28.67 (P < 0.001)	-5.31 (P < 0.001)
**Diabetes with or without complications**	279 (17.87%)	185 (19.21%)	161 (20.10%)	296 (20.60%)	287 (20.23%)	3.92 (P = 0.27)	-1.96 (P = 0.05)
**History of Myocardial infarction**	39 (2.50%)	20 (2.08%)	18 (2.25%)	33 (2.30%)	34 (2.40 %)	0.49 (P = 0.92)	0.33 (P = 0.74)
**Mild Moderate or Severe liver damage**	13 (0.83%)	11 (1.14%)	- (-%)	23 (1.60%)	17 (1.20%)	4.09 (P = 0.25)	-1.85 (P = 0.06)
**Peripheral vascular disease**	146 (9.35%)	96 (9.97%)	75 (9.36%)	155 (10.79%)	162 (1.42%)	2.05 (P = 0.56)	-1.18 (P = 0.24)
**Moderate or Severe Renal Disease**	64 (4.10%)	47 (4.88%)	36 (4.49%)	55 (3.83%)	67 (4.72%)	1.78 (P = 0.62)	0.40 (P = 0.69)
**Rheumatologic disease**	52 (3.33%)	31 (3.22%)	26 (3.25%)	29 (2.02%)	35 (2.47%)	5.69 (P = 0.13)	2.05 (P = 0.4)
**Peptic ulcer disease**	21 (1.35%)	- (-%)	14 (1.75%)	21 (1.75%)	18 (1.27%)	5.00 (P = 0.17)	-0.67 (P = 0.51)

[A]Test for differences in number of conditions by stage using Chi squared with six degrees of freedom (excluding unknown stage).
[B]Test for differences among the proportions of patients having specific comorbidities by stage (excluding unknown stage) using Chi squared with three degrees of freedom
[C]Cochran Armitage test with the rank score option to test for ordinal association between the proportion across stages (excluding unknown stage).

© 2023 Davies L et al. *JAMA Otolaryngology–Head & Neck Surgery.*

eTable 4. Characteristics of study population: US adult respondents, age 35 years and older at the time of the interview, National Health Interview Survey, 1986-2009, with mortality follow-up through 2011.

Variables	Categories	Sample size[a]	Weighted Percent
Gender	Male	172,399	47.3
	Female	230,227	52.7
Race	White	295,551	78.2
	Black	54,002	10.4
	Hispanic	43,397	8.3
	Non-Hispanic Asian[b]	9,676	3.1
Survival Status	Dead	94,049	19.5
	Alive	308,577	80.5
Age at Interview	35-39	61,214	15.1
	40-44	58,721	15.4
	45-49	51,460	13.9
	50-54	44,929	12.0
	55-59	39,561	10.1
	60-64	36,252	8.9
	65-69	34,242	7.8
	70-74	30,427	6.8
	75-79	24,805	5.4
	80-84	17,234	3.7
	85+	3,781	0.8
Health Status[c]	Excellent	102,534	26.4
	Very Good	116,916	30.0
	Good	113,139	27.6
	Fair	50,585	11.6
	Poor	19,452	4.4
Smoking Status	Current	92,753	22.4
	Former	112,317	28.6
	Never	197,556	49.0

a Sample size, total sample size is 402,626.

b Includes Pacific Islanders before 1999

c Includes health status reported by self, proxy and missing reporting status information.

© 2023 Davies L et al. *JAMA Otolaryngology–Head & Neck Surgery.*

eMethods.

eS2.1 Background

Development of personalized models to estimate prognosis involving multiple end-points utilizes competing risks which is a specialized topic in statistical methodology for the analysis of time to event data. In this application there are two possible events, death from oral cavity cancer and death from other causes. The chance of death from these two events "compete" against each other, and what is observed is the single cause of death that occurs first.

As described in the main paper, the SEER OCSC approach to the analysis of competing risks of death is novel. There are several methods for the analysis of competing risks. In a continuous time setting, Cheng et al proposed continuous time methods for predicting the cumulative incidence function under the cause-specific proportional hazards model. Their prediction methods use the same time scale for modeling of the cause-specific hazard functions for two competing events. However, this paper describes the results of estimation and prediction in the SEER Oral cancer survival calculator when discrete time competing risks data are observed and two time scales are used. Thus, the methods used in this paper do not utilize adaptations of the method developed by Cheng et al. While most competing risks survival analyses model the risk of death from cancer and other causes using the same time metric (i.e. the time since the diagnosis of cancer), we have developed a novel set of methods which jointly model the risk of cancer death on the time since diagnosis scale while modeling the risk of other causes of death on its more natural age scale.[11,12] We normally think of the risk of death in terms of age, and this more natural parameterization allows us to more easily identify and evaluate potential covariates. Lee et al[11] modified the methodology of Cheng et al[10] to allow the use of two different time scales to model the effects of the outcomes of interest. Lee's methodology allowed for simultaneously modeling both death from oral cancer and death from other causes as a function of risk factors associated with each cause of death. Lee et al[12] developed discrete time methods for competing risks predictions to utilize discrete rather than continuous time, the latter being generally available for cancer registry data. In large population-based cancer registry data, discrete event times may be observed with a large number of ties at each time point. In such cases, it may be more appropriate to use a discrete time model rather than a continuous time model because the latter may result in biased estimation. Finally, while most competing risks survival analyses model the risk of death from cancer and other causes using a single data source, Lee et al[13] extended the methods by clarifying how to conduct the modeling if each cause of death was modeled using independent or partially overlapping data sources, or using independent data sources for each cause of death even in the case where one of the data sources has a non-standard sampling schemes (i.e. complex survey design). This helps to overcome the lack of covariates in SEER data relevant to the risk of other causes of death, and allows us to have another quality that is novel: three different versions of the 'other causes of death' calculators, from which the user can select depending on the relevance to the specific patient. All of these modifications allowed us the flexibility to model competing risks in novel ways to take advantage of available data and the complexity of the risk factors and their association with the risks of the two event types.

eS2.2 Overview and Model Details

The SEER Oral Cancer Survival Calculator is a system that has four different models embedded within it to reflect competing risks of death from causes other than oral cancer (see Figure 1 in main paper). Which overall model the system can potentially employ depends both on the age of the patient at diagnosis (see eTable 5 below), and the set of variables selected to specify the risk of death from causes other than oral cancer. Each competing risk of death model specifies separate sets of covariates to estimate the risk of oral cancer death and the risk of non-oral cancer death. We refer to each set of covariates and its associated outcome within its overall model as a "sub-model". The sub-models are combined and jointly estimated using an application of the generalized estimating equation (GEE) methodology proposed in Lee et al[13]. All four models share the same oral cancer death sub-model applicable to patients diagnosed between ages 20 and 86 but differ in terms of how they estimate other cause of death (i.e. differ in terms of their other cause sub-model). The overall models (which are named after how they estimate non-oral cancer death since they all share the same cancer sub-model) are the Basic Model, The Co-existing Condition Model, the General Health Self- Assessment Model and the Subjective Assessment Option. The system provides different options for estimating the chance of non-oral cancer death depending on what type of information is available and/or most applicable for the patient under consideration. The Basic Model is available for the broadest range of ages (patients diagnosed ages 20-86) but has the least specificity in terms of factors describing

© 2023 Davies L et al. *JAMA Otolaryngology–Head & Neck Surgery.*

non-oral cancer death. The Co-existing Condition Model is available for patients aged 66-86 at diagnosis and best characterizes patients with established co-morbid conditions. The General Health Self-Assessment Model uses a self-rated measure of overall health and smoking status. While the Co-existing Condition Model characterizes serious health conditions which have been diagnosed and treated, the General Health Self-Assessment Model better characterizes the healthier end of the spectrum, and is applicable even for those who have not regularly seen a doctor prior to their diagnosis of cancer. The Subjective Assessment Option, applicable for the full spectrum of ages at diagnosis (20-86) allows one to override the assessment made by the other models if it seems like the factors in the model do not fully capture the general health of the patient at diagnosis separate from the cancer. Even though eTable5 shows the potentially applicable ages at diagnosis for each of the four models for causes of death other than oral cancer, after user testing with head and neck cancer patients, they found it difficult to negotiate having so many different models to choose from. After careful consideration and additional user testing, an algorithm for helping patients to navigate and select the most reasonable non-cancer cause of death calculator to utilize was derived (eFigure 1).

eTable 5. Applicable Ages at Diagnosis for Each of the Four Non-Oral Cancer Cause of Death Models

Age at Diagnosis	Basic Model	Co-Existing Condition Model	General Health Self-Assessment Model	Subjective Assessment Option
20-39	X			X
40-65	X		X	X
66-86	X	X	X	X

© 2023 Davies L et al. *JAMA Otolaryngology–Head & Neck Surgery*.

eFigure 1. The Flow Through the Calculator Interface to Facilitate Patient Use

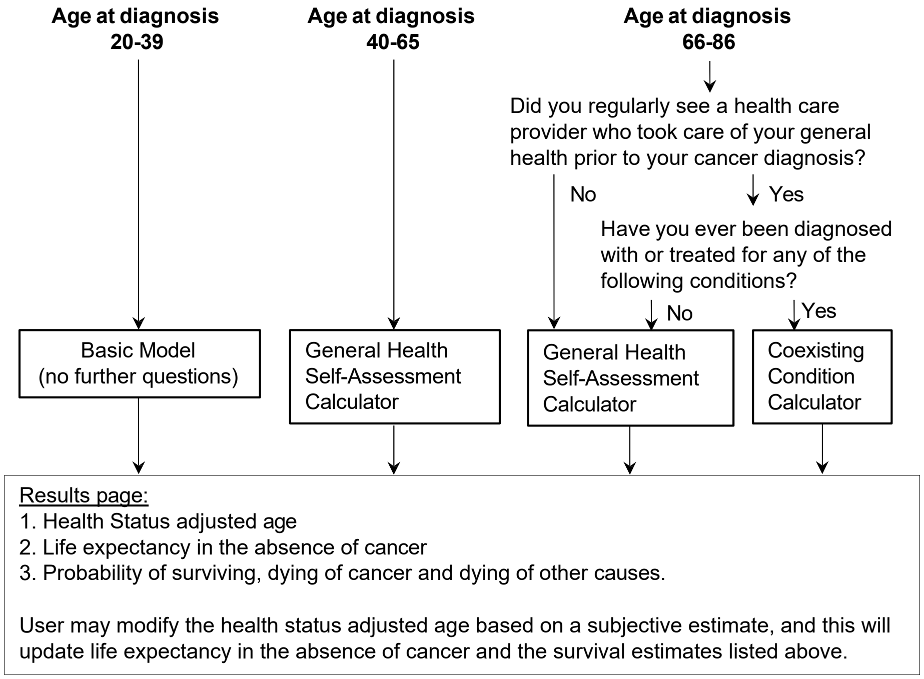

© 2023 Davies L et al. *JAMA Otolaryngology–Head & Neck Surgery*.

eAppendix 2. Other Causes of Death Submodels

eS2.2.1 Other cause of Death Sub-Model for The Basic Model

This sub-model was estimated using SEER data and utilizes the age scale as a time metric. While every individual enters this sub-model at a specified age at diagnosis x, we follow them for up to 13 years, as they reach "attained ages" of x+1, x+2, …., x+12. For individuals with attained ages 20-54 there were not enough non-oral cancer deaths to accurately model the chance of death from other causes as a function of covariates. Instead we use the use 2011 US life tables[1] which are stratified by sex and race/ethnicity (White non-Hispanic, Black non-Hispanic, Asian/Pacific Islander non-Hispanic, Hispanic). For those with attained ages of 55-97 the model is estimated using the oral cancer patients in SEER, who tend to have a higher chance of death from other causes than the general population. Because the lifetables and SEER model are conjoined, an individual diagnosed at age 53 and followed through age 58 has their annual chance of death for ages 53 and 54 computed based on the appropriate lifetable, and for ages 55, 56, 57, and 58 computed from the SEER data. Since sex is a basic important variable for the risk of death from other causes, the model for attained ages 55-97 is stratified by sex, with race/ethnicity, the socioeconomic status of the census tract of residence (based on measures developed by Yost et al[2], and adapted by Yu et al[1]) and the stage of disease as covariates.

eS2.2.2 Other Cause of Death Sub-Model for the Coexisting Condition Model

This sub-model was estimated using the SEER-Medicare linked data and utilizes the age scale as a time metric. In addition to the variables included in the Basic Model, age at diagnosis and a continuous comorbidity score computed from the year prior to diagnosis were added. As indicated in section eS1.1 we utilize a continuous composite measure of the type and severity of comorbid conditions. The continuous comorbidity score and age at diagnosis were both modeled as restricted cubic splines. Age at diagnosis was added to the model to distinguish between two individuals who have reached the same attained age, but who have different ages at diagnosis. Age at diagnosis therefore helps take into account how long ago the comorbidity score was measured.

eS2.2.3 Other Cause of Death Sub-Model for the General Health Self-Assessment Model

This sub-model is estimated using the NHIS survey data with linked mortality follow-up and utilizes age as the time metric. Although this model is used to represent death from other causes, death from any cause is substituted as a proxy, because only a very small portion of the general population dies of oral cancer (about 0.3%). Like the prior other cause of death models, this model is also stratified by sex. Other covariates include overall self-reported health status (excellent, very good, good, fair, poor) , which is a well-validated predictor of mortality including other-cause mortality in cancer patients.[5-9] Other variables include race/ethnicity, age at survey, calendar year of the survey (1986-1993, 1994-2001, 2002-2009), and smoking status (never, current, former). Age at survey was modeled as a restricted cubic spline. Age at survey, like age at diagnosis in the Co-Existing Condition Model, was added to the model to distinguish between two individuals who have reached the same attained age, but who have different ages at survey and thus the smoking status and general health assessment were measured a longer or shorter time ago.

eS2.2.4 Estimating Health Status Adjusted Age and Life Expectancy in the Absence of Cancer

For each of the three formulations of death due to non-oral cancer cause sub-models (i.e. the Basic, the Co-Existing Condition, and General Health Assessment sub-models) a set of two related metrics are calculated: health status adjusted age (HSAA) and life expectancy (LE) without cancer. Health status adjusted age is the amount added or subtracted from a person's chronologic age to account for good health or poor health. One advantage of obtaining a HSAA is that it is a common metric that can be used to compare results across all of the models. Since the computation is in the absence of oral cancer death, the cancer death sub-model is not needed to compute these metrics. To obtain a LE, the baseline hazard estimates obtained from the other cause sub-models for the Coexisting Condition Model and the General Health Self-Assessment Model were first smoothed and extrapolated to age 110 using a piecewise Gompertz model (the Gompertz is a parametric distribution usually fit to human lifetables). The baseline hazard function for each of these models represents the lifetable for the baseline covariate profile and is then multiplied by the hazard ratios corresponding to any specific patient covariate profile to obtain a life table for that profile from which a LE could be computed. For the Basic Model, LE was obtained by using 2011 US life tables (by race and sex) to obtain predicted age specific hazards of death for patients 20-54 years old and then conjoined with estimates modeled using SEER data from ages 55-96. Because the lifetables already represent smoothed estimates of the hazards of death, no further smoothing of the baseline hazards were done for this model.

For the Coexisting Condition sub-model some further adjustments were made to ensure that for any specific chronologic age as the co-morbidity score rises the HSAA monotonically rises as well. Another issue arose

© 2023 Davies L et al. *JAMA Otolaryngology–Head & Neck Surgery.*

for the calculation of LE using the General Health Self-Assessment sub-model because the health status and/or smoking status may change over the course of the life of a person, however, we only have their health and smoking status at baseline (i.e. the age of their interview). To account for this uncertainty in future smoking and health status, their health and smoking status at the time of their interview was only assumed to hold for 15 years, or age 96, whichever came first. After that time, current smokers were assumed to switch over to an estimate derived from a model in which current and former smokers were combined, and those with an excellent or very good health status were assumed to switch over to a model that combined all of the self-assessed health statuses. The LE was calculated using the primary model for the first 15 years after age at diagnosis and then one of the modified models to better reflect changes in health and/or smoking status in later years beginning in year 16 through age 110.

Using these results, a LE could be computed for a patient with a specific covariate profile for each of the three models. This LE was then matched against an all races gender-specific US life table to obtain the HSAA based on the age in the life table with a LE closest to that of the patient. The 10-year predicted annual other cause mortality rates for a patient were also obtained from the life table based on the HSAA. These rates were used in the SEER Oral Cancer Survival Calculator (rather than ones derived directly from the models) to ensure consistency between the HSAA and predicted annual mortality, although the standard errors of these rates were derived from the models.

eS2.2.5 Other Cause of Death Sub-Model for the Subjective Assessment Option

The common metrics of LE and HSAA across the three models allow one to translate between the non-oral cancer cause of death sub-models. For example, a 72 year old White non-Hispanic male with Stage III disease with congestive heart failure has approximately the same HSAA (age 80) as someone with same chronologic age, race/ethnicity, and cancer characteristics but a current smoker and fair self-assessed overall health (age 79). This feature allows the user to assess the LE and HSAA using each of the three models, and then make further modifications to the HSAA and associated LE to subjectively account for any other factors not included in these models, or to use an external calculator which computes a person's biological age as a combination of any types of factors. . This assessment might be based on the functional limitations of the patient (i.e. difficulties in the completion of everyday tasks), risk factors such as obesity, genetic factors, the patient's psychological well-being, or any other factors which are deemed relevant. This subjective assessment paired with the cancer death sub-model is called the Subjective Assessment Option.

eS2.2.6 Oral Cancer Death Sub-Model for All Four Models

This sub-model uses SEER data for patients aged 20-65 and SEER Medicare linked data for patients aged 66-94 at diagnosis. This is the common cancer sub-model paired with all 4 other cause of death sub-models. Unlike the other cause of death sub-models, this sub-model utilizes time since diagnosis as the time scale. Separate models were developed for each stage of disease using oral cancer death using the Howlader algorithm[14] as the endpoint of interest and treating other causes of death and individuals still alive at the end of the study period as censored observations. Variables that were available for inclusion in the models were age at diagnosis, sex, race, diagnosis year, marital status, socioeconomic status, substages of Stage IV disease (stages IVA, IVB, IVC, IV NOS), grade of the tumor, pathologic versus clinical staging of the tumor and lymph nodes, and comorbidity score for those aged 66 and above. Diagnosis year was included so that when estimating survival for a recently diagnosed patient the estimates can be adjusted for temporal improvements in survival. Pathologic versus clinical staging of the tumor and nodes is an important explanatory variable, because patients who are not well enough to undergo extirpative surgery of their oral cancer and/or staging of the neck via neck dissection for example are likely to be different (i.e., more ill or frail) than those who are able to undergo surgery. These factors would reasonably be expected to affect cancer survival. The socioeconomic status variable is described in eS 1.1. Indicator variables were used to model the quintiles of SES and the quintiles were combined into larger groups based on the statistical significance of the regression coefficients for the indicator variables. Comorbidity was included as prognostic variable for oral cancer death because severe comorbidities may interfere with the treatment of the cancer. It was included as two indicator variables (under age 66 without a comorbidity score, age 66+ and with a comorbidity score indicating very severe comorbidities or combination of comorbidities - a score of 1000+). While a score above 560 was considered severe with respect to its impact on death from causes other than oral cancer, some empirical investigations indicated that we needed a higher cutoff to evaluate the impact of comorbidities on oral cancer death. The reference group represented those aged 66 and above with a score under 1000. When a patient uses the system, a comorbidity score is only directly available in the OCSC for those who enter comorbidity conditions as part of the Co-existing Condition model. However, a HSAA is available for all patients regardless of which model they use. We took advantage of this, mapping the HSAA information and their chronologic age back to comorbidity to create a

© 2023 Davies L et al. *JAMA Otolaryngology–Head & Neck Surgery.*

synthetic comorbidity score. This synthetic score is then used to categorize the patient into a score of >1000 or <1000. This allows us to approximate which patients aged 66+ would have a score of 1000+ for all users of the system regardless of which model they use.

© 2023 Davies L et al. *JAMA Otolaryngology–Head & Neck Surgery*.

eAppendix 3. Model Estimation

Formulating the discrete time cause-specific hazard models as longitudinal binary regression models provides great flexibility in model specification and such models can be easily estimated under complex sampling schemes. For simultaneous estimation of models for different event types, single and double coding may be employed, facilitating the use of standard software for correlated outcomes. Estimation **may be accomplished using standard software, and prediction is accomplished using a very generic program, which is provided in the Supplementary Materials** of Lee et al.[13] The sub-models are combined and jointly estimated using an application of the generalized estimating equation (GEE) methodology proposed in Lee et al.[13] For the Basic Model and Coexisting Condition Model, GEE models were fit using Proc Genmod in SAS specifying log-binomial regression models with an independence working correlation structure. For the General Health Self-Assessment Model, because the two datasets (SEER and NHIS) are independent, the two sub-models are fit separately and then combined assuming a zero covariance between each sub-model. In this case, the oral cancer mortality sub-model was fit using Proc Genmod in SAS (without the GEE option) specifying a log-binomial regression model. The other cause of death sub-model was also fit using a log-binomial regression model, but the complex survey design and weights were accounted for using Stata 13 with survey commands (StataCorp, College Station, TX). All analyses were done using a year as the discrete time metric.

To facilitate estimation, one may employ single coding for individuals that contribute to the analysis of the discrete cause-specific hazard function of only a single event type and double coding for individuals that contribute to analyses of both discrete cause-specific hazard functions of two event types. The structure of the datasets are changed from having the standard one record for each individual to having one record at each discrete time on the time since diagnosis time scale and/or on the age time scale until an individual has the event of interest of each dataset or is censored. Single coding is used in situations where all individuals contribute person time at risk to only a single event type, e.g, for the General Health Self-Assessment Model individuals in SEER contribute to the hazard of death from oral cancer, while individuals surveyed in NHIS contribute to the hazard of death from other causes. Double coding is used in situations where every individual contributes to both causes of death. For the SEER OCSC, both the Basic and the Co-Existing Condition models represent situations which include both single and double coded individuals. For example in the Co-Existing Condition model, individuals in SEER (with Medicare linkage) diagnosed at ages 66-94 contribute to the risk of death from both causes of death, while those in SEER diagnosed at ages 50-65 with attained ages 55 and older contribute only to the risk of death from other causes of death.

eTable 6 demonstrates an example of coding of three exemplar patients for the Basic Model. Patient 1, who is diagnosed at age 73 and dies of oral cancer during year 3, contributes three records to the oral cancer sub-model ending in an event, and three records to the other cause of death sub-model ending in a censored observation because the patient dies of oral cancer. Patient 2 is diagnosed at age 54 and dies during year 4 of a cause other than oral cancer. This is an example of a patient who contributes unequal number of person years to each sub-model. This person contributes three records to the other cause of death sub-model (because patients diagnosed ages 50-94 are included, although they only contribute person years after they reach an attained age of 55), but contribute the full four years to the oral cancer sub-model. Patient 3, who is diagnosed at age 45 and is still alive at the end of year 3 is single coded contributing only to the oral cancer death sub-model.

eTable 7 demonstrates coding of two exemplar patients for the Co-existing Condition Model. Patient 1, who is diagnosed at age 68 and dies of oral cancer within 3 years, is double coded and contributes three records to each sub-model. Patient 2, who is diagnosed at age 46 and dies of oral cancer in year 5, contributes only to the oral cancer sub-model.

eTable 8 demonstrates coding for the General HealthSelf Assessment Model using an exemplar patient from the SEER database, and an exemplar individual from NHIS (not a patient since this is a general population survey). Patient 1, who is diagnosed at age 53 and dies of oral cancer during year 3, contributes three records to the oral cancer sub-model. Individual 2, who is interviewed at age 50 and is alive at the end of follow-up, contributes 5 records to the other cause of death sub-model, without contributing any events (but just person-time at risk).

© 2023 Davies L et al. *JAMA Otolaryngology–Head & Neck Surgery.*

eTable 6. Examples of Single and Double Coding of Patient Records for the Basic Model. Data used for each sub-model comes from SEER.

Sample Patient ID	Patient Description	Event Status during the interval	Oral Cancer Mortality Sub-model		Other Cause Mortality Sub-model	
			Time Since Diagnosis	Covariate for Oral Cancer Mortality	Attained Age at start of interval	Covariate for Other Cause Mortality
1 Double Coding	Dx age 73, died of oral cancer in year 3, covariates $Z_{1,CANCER}$ $Z_{1,OTHER}$	0	1	$Z_{1,\ CANCER}$	---	---
		0	2	$Z_{1,\ CANCER}$	---	---
		1	3	$Z_{1,\ CANCER}$	---	---
		0	---	---	73	$Z_{1,\ OTHER}$
		0	---	---	74	$Z_{1,\ OTHER}$
		0	---	---	75	$Z_{1,\ OTHER}$
2 Double Coding	Dx age 54, died of other cause in year 4, covariates $Z_{2,CANCER}$ $Z_{2,OTHER}$	0	1	$Z_{2,\ CANCER}$	---	---
		0	2	$Z_{2,\ CANCER}$	---	---
		0	3	$Z_{2,\ CANCER}$	---	---
		0	4	$Z_{2,\ CANCER}$	---	---
		0	---	---	55	$Z_{2,\ OTHER}$*
		0	---	---	56	$Z_{2,\ OTHER}$
		1	---	---	57	$Z_{2,\ OTHER}$
3 Single Coding	Dx age 45, alive in year 3, covariate $Z_{3,CANCER}$	0	1	$Z_{3,\ CANCER}$	---	---
		0	2	$Z_{3,\ CANCER}$	---	---
		0	3	$Z_{3,\ CANCER}$	---	---
		Coding of other cause mortality not applicable				

*This patient was diagnosed in the age range 50-54 and will contribute to estimation of the other cause mortality sub-model since he/she survived beyond age 55.

© 2023 Davies L et al. *JAMA Otolaryngology–Head & Neck Surgery*.

eTable 7. Examples of Single and Double Coding of Patient Records for the Coexisting Condition Model. Data used for each sub-model came from SEER for patients diagnosed ages 20-65, and SEER_Medicare linked data for patients diagnosed ages 66-94.

Sample Patient ID	Patient Description	Event Status during the interval	Oral Cancer Mortality Sub-model		Other Cause Mortality Sub-model	
			Time Since Diagnosis	Covariate for Oral Cancer Mortality	Attained Age at start of interval	Covariate for Other Cause Mortality
1 Double Coding (within age range of both sub-models)	Dx age 68, died of oral cancer during year 3 covariates $Z_{1,CANCER}$ $Z_{1,OTHER}$	0	1	$Z_{1, CANCER}$	---	---
		0	2	$Z_{1, CANCER}$	---	---
		1	3	$Z_{1, CANCER}$	---	---
		0	---	---	68	$Z_{1, OTHER}$
		0	---	---	69	$Z_{1, OTHER}$
		0	---	---	70	$Z_{1, OTHER}$
2 Single Coding for cancer (only within age range of oral cancer mortality model)	Dx age 46, died of cancer during year 5 covariate $Z_{2,CANCER}$	0	1	$Z_{2, CANCER}$	---	---
		0	2	$Z_{2, CANCER}$	---	---
		0	3	$Z_{2, CANCER}$	---	---
		0	4	$Z_{2, CANCER}$	---	---
		1	5	$Z_{2, CANCER}$	---	---
		Coding of other cause mortality not applicable				

© 2023 Davies L et al. *JAMA Otolaryngology–Head & Neck Surgery*.

eTable 8. Example of Single Coding of Patient Records for General Health Self-Assessment Model. Cancer survival experience comes from SEER data, and other cause survival experience comes from NHIS data; two independent datasets.

Sample Patient (if SEER) or Individual (if NHIS) ID	Patient/Indivdual Description	Event record for Time Since Diagnosis	Oral Cancer Mortality Sub-model		Other Cause Mortality Sub-model	
			Time Since Diagnosis	Covariate for Oral Cancer Mortality	Attained Age at start of interval	Covariate for Other Cause Mortality
1 (SEER dataset)	Dx age 53, died of oral cancer at 3 years, covariate $Z_{1,CANCER}$	0	1	$Z_{1, CANCER}$	---	---
		0	2	$Z_{1, CANCER}$	---	---
		1	3	$Z_{1, CANCER}$	---	---
		Coding of other cause mortality not applicable				
2 (NHIS Dataset)	Interviewed at age 50, alive at 5 years, Covariate $Z_{2,ALL}$	Coding of cancer mortality not applicable				
		0	---	---	50	$Z_{2,ALL}$
		0	---	---	51	$Z_{2,ALL}$
		0	---	---	52	$Z_{2,ALL}$
		0	---	---	53	$Z_{2,ALL}$
		0	---	---	54	$Z_{2,ALL}$

* Death from all causes was treated as death from other causes than oral cancer because a very small percentage of the general population dies of oral cancer (about 0.3%).

For each of the models, various interactions were added based on their conceptual plausibility, and backward variable selection was used to identify the variables to be used for survival estimation. After fitting the full model, the variable with the largest P value was removed and the model refit with the remaining variables. Main effects of any interaction terms were not deleted even if not significant if associated interaction terms were significant. This process was continued until all main effect variables in a model had P values of </= 0.20, and interaction terms had p values </= 0.10. Further examination of the models was conducted to reduce the levels of some covariates. The proportional hazards assumption was tested using graphical methods, and interactions with time since diagnosis or age were added when necessary to remedy issues where lack of proportional hazards was found.

Predicted cumulative mortality functions for cancer death and other cause of death (and their standard errors) for a specified set of cancer and other cause covariates were computed using equations (6) and (7) in Lee et al[13], and code to perform these calculations is available in R in the supplemental materials of Lee et al.[13] The code requires input of the point estimates and variance-covariance matrix output from the model fitting step.

© 2023 Davies L et al. *JAMA Otolaryngology–Head & Neck Surgery.*

eAppendix 4. Model Estimates

eS4.1 Parameter Estimates for the Oral Cancer Death Sub-Models

The parameter estimates for the oral cancer death sub-model is given in eTable 9 (Stage I), eTable 10 (Stage II), eTable 11 (Stage III) and eTable 12 (Stage IV). Restricted Cubic Splines were used for the variables age at diagnosis and comorbidity score which means that the coefficients and hazard ratios for these variables cannot be interpreted in the usual way. Also, interaction terms involving these variables were introduced in some models to account for expected underlying relationships. Restricted cubic splines, which are used to model some of the continuous variables and interactions between continuous variables and categorial variables, do not produce hazard ratios which are directly interpretable.

At all stages, as would be expected in oral cancer, which is primarily treated with surgery as a first course of treatment, patients with only clinical staging information available rather than pathologic staging (indicating they had not undergone surgery) showed results in the model consistent with having a higher risk of death due to the cancer. For example, the hazard ratio was 2.67 for stage I (S.E. 0.24, p<0.001), 1.87 for stage III (S.E. 0.09, p <0.001), and 1.63 for stage IV (S.E. 0.05, p<0.001) (for stage II, the results depend on the age of the patient, which was modeled as an interaction using a restricted cubic spline).

Histologic grade, although not included in the head and neck cancer TNM staging system[15], was found nonetheless to be important in modeling cancer as a cause of death. Risk of death due to the cancer increased in stepwise fashion by grade (reference group grade I), with the impact of grade diminishing with increasing stage of disease: in the stage I model, cancers that were grade II had a hazard ratio of 1.71 (standard error 0.12, p <0.001) and grade III or IV had a hazard ratio of 2.67 (standard error 0.23, p <0.001). In the stage III model, cancers that were grade II had a hazard ratio of 1.33 (standard error 0.09, p <0.001) and grade III or IV had a hazard ratio of 1.88 (standard error 0.16, p <0.001). In the stage IV model, cancers that were grade II had a hazard ratio of 1.12 (S.E. 0.04, p=0.001), and grade III/IV a hazard ratio of 1.21 (S.E. 0.05, p <0.001).

In every case where comorbidity was found to be important (Stages I, II and IV) patients under age 66 and those age 66 and above with a comorbidity score <1000 (the reference category) did not have a different risk of death from oral cancer (after adjusting for all other covariates) and thus these two categories were collapsed into a single category.

eS4.2 Baseline Survival Curves by Stage Derived from the Oral Cancer Death Sub-models

eFigure 2 provides survival curves for the baseline hazards by stage for the cancer sub-model to illustrate the effect of stage on the risk of death from oral cancer. A reason for the differences in the number of years of follow-up associated with the survival curves by stage included the number of events (death due to oral cancer) being small at the tail of the survival curve for Stages II, III and IV creating some instability. Therefore, the length of follow-up was limited in some cases in order to obtain model convergence.

eS4.3 Parameter Estimates for the Other Cause of Death Sub-Models

Probabilities of death and Hazard Ratios of dying for non-oral cancer death sub-models for each of the models are given in eTables 13 (males) and 14 (females) for the Basic Model, eTables 15 (males) and 16 (females) for the Coexisting Condition Model, and eTables 17 (males) and 18 (females) for the General Health Self-Assessment Model. The baseline parameters as a function of age in each model provides a life table for causes of death other than oral cancer, which forms the baseline for all other patient covariate profiles. In the other cause of death sub-models, the risk of death from causes other than cancer was higher at higher cancer stages. One potential interpretation for this is that patients diagnosed with later stage cancers tend to also have more health conditions. However, comorbidities did not vary widely across stage at diagnosis. Thus, an alternative interpretation is that the presence of more advanced staged cancers increases the risk of death not just from cancer but from other causes as well, because of the additional burden of the cancer on the person's physiology. In the SEER OCSC Basic Model, the risk of death from other causes increased as cancer stage increased (reference group stage I cancer), with the hazard ratio for men with stage IV disease, was 1.52 (S.E. 0.09, p<0.001) and for women the hazard ratio was 1.52 (S.E. 0.11, p<0.001). This persisted, though to a lesser degree, in the Coexisting Conditions Model, which controlled for comorbidity. Among men with stage IV disease, the hazard ratio was 1.36 (S.E. 0.10, p<0.001) and among women the hazard ratio was 1.37 (S.E. 0.09, p<0.001). This shows that measuring severe comorbid conditions alone does not fully capture all factors that might be associated with a greater risk of death at higher stages, such as the likelihood of seeking or ability to seek medical attention, or the role of milder health conditions not captured in the data.

eTable 9. Probabilities of Death and Hazard Ratios of Dying for Oral Cancer Cause of Death Submodel for Stage I

**Reference Group (Baseline Hazards):** Males, Age at Diagnosis 20, Middle to Highest SES Quintiles, Year of Diagnosis 2000-2003, Unmarried, Non-Hispanic Whites, Path T and/or Path N and Grade I.<br>Time Since Diagnosis	Probability of Death	Standard Error	p Value
Year 1	0.0047	0.0021	<.0001
Year 2	0.0092	0.0040	<.0001
Year 3	0.0068	0.0030	<.0001
Year 4	0.0043	0.0019	<.0001
Year 5	0.0032	0.0014	<.0001
Year 6	0.0026	0.0012	<.0001
Year 7	0.0032	0.0014	<.0001
Year 8	0.0033	0.0015	<.0001
Year 9	0.0036	0.0017	<.0001
Year 10	0.0037	0.0017	<.0001
Year 11	0.0026	0.0013	<.0001
Year 12	0.0031	0.0017	<.0001
Year 13	0.0015	0.0012	<.0001

Race/Ethnicity			
Non-Hispanic White	1	-	-
Non-Hispanic Black	1.3210	0.1708	0.0313
Non-Hispanic API	1.1559	0.1283	0.1916
Hispanic	1.6408	0.2430	0.0008
Hispanic over Year 3 and after	0.4987	0.1264	0.0061
Clinical or pathologic staging of the primary tumor and nodes			
Path T and/or Path N	1	-	-
Clin T Clin N	2.6792	0.2419	<.0001
Grade I	1	-	-
Grade II	1.7110	0.1157	<.0001
Grade III/IV	2.6706	0.2337	<.0001
Socio-economic level (quintiles)			
SES 1 (lowest quintile)	1.2119	0.0928	0.0122
SES 2 (2nd lowest quintile)	1.1268	0.0835	0.1069
SES 3/4/5 (middle to highest quintile)	1	-	-
Year of diagnosis			
2000-2003	1	-	-
2004-2007	0.8359	0.0542	0.0057
2008-2011	0.5869	0.0475	<.0001

© 2023 Davies L et al. *JAMA Otolaryngology–Head & Neck Surgery.*

**eTable 9 (Cont.). Probabilities of death and Hazard Ratios of dying for oral cancer cause of death sub-model for stage I.**			
Covariates	Hazard Ratio	Standard Error	p Value
Comorbid and Age at diagnosis			
Under 66 or Above 66 with Comorbidity Score <1000	1	-	-
Above 66 with Comorbidity Score = 1000+	1.3221	0.1881	0.0498
Age at diagnosis			
Spline term 0	1.0445	0.0166	0.0063
Spline term 1	0.9132	0.0405	0.0406
Spline term 2	1.4155	0.2497	0.0489
Sex			
Male	-	-	-
Female	0.9116	0.4581	0.8539
Marital Status			
Unmarried	-	-	-
Married	1.8829	0.9322	0.2012
Age at diagnosis * Sex			
Spline term 0 * Female	0.9962	0.0183	0.8343
Spline term 1 * Female	1.0441	0.0520	0.3857
Spline term 2 * Female	0.8566	0.1658	0.4241
Age at diagnosis * Marital Status			
Spline term 0 * Married	0.9676	0.0175	0.0685
Spline term 1 * Married	1.1037	0.0543	0.0448
Spline term 2 * Married	0.7166	0.1371	0.0815

© 2023 Davies L et al. *JAMA Otolaryngology–Head & Neck Surgery*.

eTable 10. Probabilities of Death and Hazard Ratios of Dying for Oral Cancer Cause of Death Submodel for Stage II

**Reference Group (Baseline Hazards)**: Males, Age at Diagnosis 20-40, Year of Diagnosis 2000-2007, Unmarried, (Non-Hispanic Whites, Non-Hispanic blacks and Non-Hispanic API), Path T and/or Path N and Grade I. Time Since Diagnosis	Probability of Death	Standard Error	p Value
Year 1	0.0513	0.0098	<.0001
Year 2	0.0532	0.0104	<.0001
Year 3	0.0351	0.0070	<.0001
Year 4	0.0161	0.0036	<.0001
Year 5	0.0189	0.0042	<.0001
Year 6	0.0179	0.0042	<.0001
Year 7	0.0144	0.0037	<.0001
Year 8	0.0125	0.0036	<.0001
Year 9	0.0116	0.0037	<.0001
Year 10	0.0114	0.0041	<.0001
Year 11	0.0142	0.0055	<.0001

Covariates	Hazard Ratio	Standard Error	p Value
Grade			
Grade I	1	-	-
Grade II	1.3291	0.0894	<.0001
Grade III/IV	1.8772	0.1633	<.0001
Year of Diagnosis			
2000-2007	1	-	-
2008-2011	0.8212	0.0544	0.0029
Comorbid Conditions and Age at diagnosis			
Under 66 or Above 66 with Comorbidity Score <1000	1	-	-
Above 66 with Comorbidity Score = 1000+	1.4708	0.1561	0.0003
Age at Diagnosis			
Spline term 0	1.0307	0.0171	0.0694
Spline term 1	0.9253	0.0417	0.0853
Spline term 2	1.3350	0.2215	0.0816
Race/Ethnicity			
NH White/NH Black/NH API	-	-	-
Hispanic	2.4677	0.6668	0.0008
Sex			
Male	-	-	-
Female	0.8580	0.2062	0.524
Marital Status			
Unmarried	-	-	-
Married	0.6788	0.1476	0.0747

© 2023 Davies L et al. *JAMA Otolaryngology–Head & Neck Surgery.*

eTable 10 (Cont.). Probabilities of death and Hazard Ratios of dying for oral cancer cause of death sub-model for stage II.			
Covariates	Hazard Ratio	Standard Error	p Value
Clinical or pathologic staging of the primary tumor and nodes			
Path T and/or Path N	-	-	-
Clin T Clin N	2.0790	0.5401	0.0048
Age at diagnosis * Race/Ethnicity			
Spline term 0 * Hispanic	0.9372	0.0275	0.0271
Spline term 1 * Hispanic	1.1147	0.0974	0.2143
Spline term 2 * Hispanic	0.7401	0.2372	0.3477
Age at diagnosis * Sex			
Spline term 0 * Female	0.9866	0.0210	0.5269
Spline term 1 * Female	1.0604	0.0568	0.2743
Spline term 2 * Female	0.8592	0.1599	0.4147
Age at diagnosis * Marital Status			
Spline term 0 * Married	0.9929	0.0194	0.7159
Spline term 1 * Married	1.0659	0.0532	0.2014
Spline term 2 * Married	0.7808	0.1365	0.1569
Age at diagnosis * Clinical or pathologic staging of the primary tumor and nodes			
Spline term 0 * Clin T Clin N	0.9809	0.0223	0.3945
Spline term 1 * Clin T Clin N	1.0709	0.0594	0.2177
Spline term 2 * Clin T Clin N	0.8072	0.1513	0.2529

© 2023 Davies L et al. *JAMA Otolaryngology–Head & Neck Surgery.*

eTable 11. Probabilities of Death and Hazard Ratios of Dying for Oral Cancer Cause of Death Submodel for Stage III

**Reference Group (Baseline Hazards)**: Males, Age at Diagnosis 20-40, Highest SES Quintile, Year of Diagnosis 2000-2003, Unmarried, (Hispanic, Non-Hispanic Whites and Non-Hispanic API), Path T and/or Path N and Grade I. Time Since Diagnosis	Probability of Death	Standard Error	p Value
Year 1	0.1747	0.0237	<.0001
Year 2	0.1710	0.0236	<.0001
Year 3	0.0953	0.0144	<.0001
Year 4	0.0531	0.0092	<.0001
Year 5	0.0553	0.0099	<.0001
Year 6	0.0420	0.0086	<.0001
Year 7	0.0180	0.0056	<.0001
Year 8	0.0347	0.0090	<.0001
Year 9	0.0385	0.0108	<.0001
Year 10	0.0428	0.0127	<.0001
Year 11	0.0291	0.0121	<.0001
Year 12	0.0246	0.0143	<.0001

Covariates	Hazard Ratio	Standard Error	p Value
Race/Ethnicity			
Hispanic/NH White/NH API	1	-	-
NH Black	1.1310	0.0848	0.101
Clinical or pathologic staging of the primary tumor and nodes			
Path T and/or Path N	1	-	-
Clin T Clin N	1.8714	0.0945	<.0001
Grade			
Grade I	1	-	-
Grade II	1.1157	0.0696	0.0793
Grade III/IV	1.3857	0.0994	<.0001
Marital Status			
Unmarried	1	-	-
Married	0.7952	0.0383	<.0001
Age at diagnosis			
Spline term 0	0.9880	0.0145	0.4117
Spline term 1	1.0104	0.0443	0.8145
Spline term 2	0.9896	0.1590	0.9478
Socio-economic level (quintiles)			
SES 1 (lowest quintile)	1.3767	0.2759	0.1107
SES 2/3/4 (middle quintiles)*	3.3619	6.2454	0.514
SES 5 (highest quintile)	-	-	-
Year of Diagnosis			
2000-2003	-	-	-
2004-2011	0.5149	0.0897	0.0001

© 2023 Davies L et al. *JAMA Otolaryngology–Head & Neck Surgery.*

eTable 11 (Cont.). Probabilities of death and Hazard Ratios of dying for oral cancer cause of death sub-model for stage III.			
Covariates	Hazard Ratio	Standard Error	p Value
Age at diagnosis * Socio-economic level (quintiles)			
Spline term 0 * SES 1 (lowest quintile)	0.9774	0.0213	0.2922
Spline term 1 * SES 1 (lowest quintile)	1.0851	0.0625	0.156
Spline term 2 * SES 1 (lowest quintile)	0.7884	0.1557	0.2288
Spline term 0 * SES 2/3/4 (middle quintiles)*	0.9028	0.1215	0.4477
Spline term 1 * SES 2/3/4 (middle quintiles)*	1.2528	0.2731	0.3012
Spline term 2 * SES 2/3/4 (middle quintiles)*	0.5253	0.2970	0.2548
Age at diagnosis * Year of Diagnosis			
Spline term 0 * 2004-2011	1.0328	0.0186	0.0732
Spline term 1 * 2004-2011	0.9454	0.0424	0.209
Spline term 2 * 2004-2011	1.2112	0.1800	0.1972

*The group of SES 2/3/4 (middle SES quintiles) is collapsed with SES 5 (highest SES quintile) for those age at diagnosis less than 56.

© 2023 Davies L et al. *JAMA Otolaryngology–Head & Neck Surgery*.

eTable 12. Probabilities of Death and Hazard Ratios of Dying for Oral Cancer Cause of Death Submodel for Stage IV

**Reference Group (Baseline Hazards)**: Males, Age at Diagnosis 20-40, Other Than Lowest SES Quintile, Year of Diagnosis 2000-2003, Unmarried, Non-Hispanic White and Non-Hispanic API, Path/Clin T Path N, Grade I and Stage IV A and B. Time Since Diagnosis	Probability of Death	Standard Error	p Value
Year 1	0.2496	0.0196	<.0001
Year 2	0.2101	0.0167	<.0001
Year 3	0.1129	0.0102	<.0001
Year 4	0.0665	0.0073	<.0001
Year 5	0.0470	0.0063	<.0001
Year 6	0.0487	0.0072	<.0001
Year 7	0.0492	0.0081	<.0001
Year 8	0.0274	0.0067	<.0001
Year 9	0.0258	0.0072	<.0001

Covariates	Hazard Ratio	Standard Error	p Value
Race/Ethnicity			
NH White/NH API	1	-	-
Hispanic	1.0880	0.0428	0.0318
NH Black	1.0372	0.0383	0.3223
Clinical or pathologic staging of the primary tumor and nodes			
Path/Clin T Path N	1	-	-
Path T Clin N	0.9189	0.0497	0.118
Clin T Clin N	1.6315	0.0473	<.0001
Grade			
Grade I	1	-	-
Grade II	1.1228	0.0408	0.0014
Grade III/IV	1.2130	0.0489	<.0001
Socio-economic level (quintiles)			
SES 1 (lowest quintile)*	1.1117	0.0396	0.0029
SES 2/3/4/5 (middle to highest quintile(s))	1	-	-
Year of Diagnosis			
2000-2003	1	-	-
2004-2007	0.9332	0.0274	0.0189
2008-2011	0.8496	0.0257	<.0001
Comorbid and Age at diagnosis			
Under 66 or Above 66 with Comorbidity Score <1000	1	-	-
Above 66 with Comorbidity Score = 1000+	1.1616	0.0560	0.0019
Age at diagnosis			
Spline term 0	0.9968	0.0068	0.6378
Spline term 1	1.0229	0.0205	0.26
Spline term 2	0.9505	0.0672	0.4729

© 2023 Davies L et al. *JAMA Otolaryngology–Head & Neck Surgery.*

eTable 12 (Cont). Probabilities of death and Hazard Ratios of dying for oral cancer cause of death sub-model for stage IV.			
Covariates	Hazard Ratio	Standard Error	p Value
Marital Status			
Unmarried	-	-	-
Married	0.7965	0.0798	0.0232
Stage IV subcategory			
Stage IV A / B	-	-	-
Stage IV NOS	0.8430	0.0998	0.1489
Stage IV C	1.4269	0.2518	0.044
Age at diagnosis * Marital Status			
Spline term 0 * Married	1.0038	0.0101	0.7104
Spline term 1 * Married	0.9900	0.0278	0.718
Spline term 2 * Married	1.0602	0.1029	0.5465
Age at diagnosis * Stage IV subcategory			
Spline term 0 * Stage IV NOS	0.9992	0.0120	0.9446
Spline term 1 * Stage IV NOS	1.0255	0.0340	0.4468
Spline term 2 * Stage IV NOS	0.9048	0.1022	0.3758
Spline term 0 * Stage IV C	1.0136	0.0172	0.4275
Spline term 1 * Stage IV C	0.9632	0.0437	0.4085
Spline term 2 * Stage IV C	1.0677	0.1686	0.6781

* The group of SES 1 (lowest SES quintile) is collapsed with SES 2/3/4/5 for those age at diagnosis greater than 65.

© 2023 Davies L et al. *JAMA Otolaryngology–Head & Neck Surgery*.

eFigure 2. Cancer Death Submodel Baseline Survival by Stage

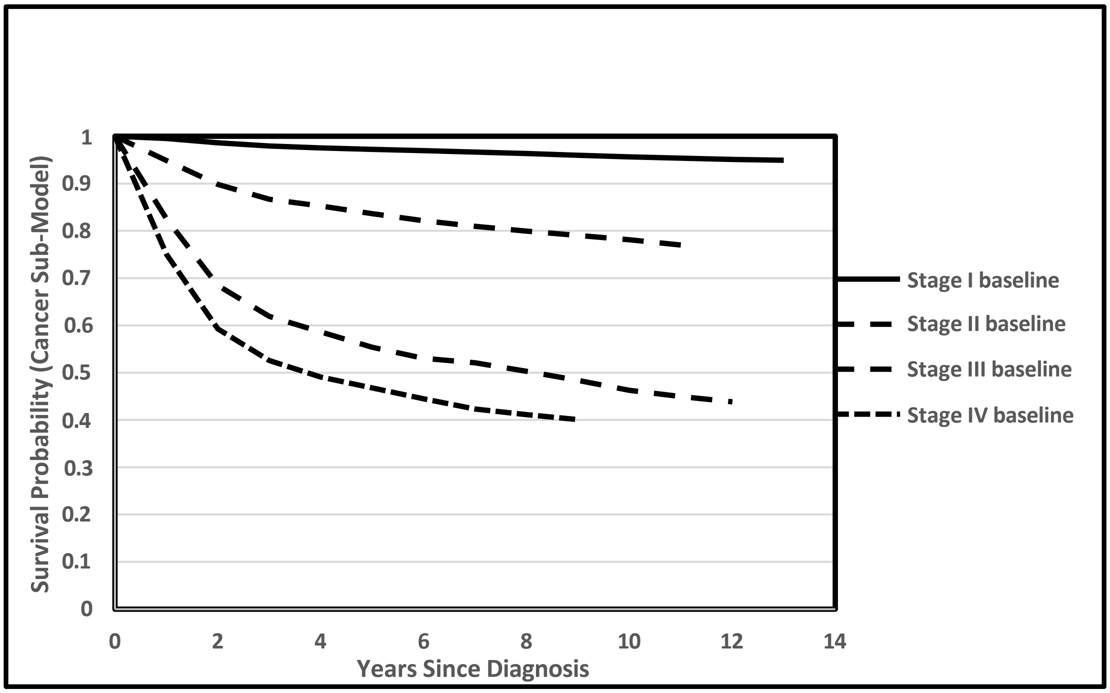

Definition of baseline hazards by stage:
Stage I: Male, age at diagnosis 20, middle to highest SES quintiles, year of diagnosis 2000-2003, unmarried, non-Hispanic white, path T and/or path N and grade I.
Stage II: Male, age at diagnosis 20-40, year of diagnosis 2000-2007, unmarried, non-Hispanic (non-Hispanic white, non-Hispanic black and non-Hispanic API), path T and/or path N and grade I.
Stage III: Male, age at diagnosis 20-40, highest SES quintile, year of diagnosis 2000-2003, unmarried, other than non-Hispanic Black (Hispanic, non-Hispanic white and non-Hispanic API), path T and/or path N and grade I.
Stage IV: Male, age at diagnosis 20-40, other than lowest SES quintiles, year of diagnosis 2000-2003, unmarried, non-Hispanic white and non-Hispanic API, path/clin T path N, grade I and stage IV A and B.

© 2023 Davies L et al. *JAMA Otolaryngology–Head & Neck Surgery*.

eTable 13. Probabilities of Death and Hazard Ratios of Dying for the Other Cause of Death Submodel in the Basic Model (Males)

**Reference Group (Baseline Hazards):** Non-Hispanic Whites and Non-Hispanic Blacks, Highest SES Quintile and Stage I. Attained Age	Probability of Death	Standard Error	p Value
Age 55	0.0161	0.0027	<.0001
Age 56	0.0199	0.0030	<.0001
Age 57	0.0140	0.0024	<.0001
Age 58	0.0192	0.0028	<.0001
Age 59	0.0184	0.0026	<.0001
Age 60	0.0177	0.0026	<.0001
Age 61	0.0221	0.0030	<.0001
Age 62	0.0213	0.0029	<.0001
Age 63	0.0200	0.0028	<.0001
Age 64	0.0203	0.0029	<.0001
Age 65	0.0228	0.0031	<.0001
Age 66	0.0277	0.0036	<.0001
Age 67	0.0235	0.0033	<.0001
Age 68	0.0294	0.0038	<.0001
Age 69	0.0284	0.0037	<.0001
Age 70	0.0251	0.0036	<.0001
Age 71	0.0311	0.0042	<.0001
Age 72	0.0220	0.0035	<.0001
Age 73	0.0370	0.0048	<.0001
Age 74	0.0371	0.0049	<.0001
Age 75	0.0436	0.0055	<.0001
Age 76	0.0463	0.0060	<.0001
Age 77	0.0482	0.0062	<.0001
Age 78	0.0383	0.0056	<.0001
Age 79	0.0414	0.0061	<.0001
Age 80	0.0395	0.0061	<.0001
Age 81	0.0527	0.0074	<.0001
Age 82	0.0466	0.0073	<.0001
Age 83	0.0700	0.0095	<.0001
Age 84	0.0708	0.0100	<.0001
Age 85	0.0689	0.0105	<.0001
Age 86	0.0825	0.0125	<.0001
Age 87	0.0746	0.0124	<.0001
Age 88	0.0962	0.0162	<.0001
Age 89	0.1003	0.0184	<.0001
Age 90	0.0846	0.0178	<.0001
Age 91	0.1284	0.0227	<.0001
Age 92	0.1616	0.0302	<.0001
Age 93	0.0849	0.0273	<.0001
Age 94	0.1643	0.0404	<.0001
Age 95	0.0863	0.0471	<.0001
Age 96	0.0628	0.0608	0.0043
Age 97	0.2682	0.1095	0.0013

© 2023 Davies L et al. *JAMA Otolaryngology–Head & Neck Surgery.*

eTable 13 (Cont.). Probabilities of death and Hazard Ratios of dying for the other cause of death sub-model in the Basic Model (Males).			
Covariates	Hazard Ratio	Standard Error	p Value
Race/Ethnicity			
NH White/NH Black	1	-	-
Hispanic	0.8372	0.0798	0.0621
NH API	0.6656	0.0753	0.0003
Stage			
Stage I	1	-	-
Stage II	1.2628	0.0727	<.0001
Stage III	1.3505	0.0898	<.0001
Stage IV	1.5160	0.0860	<.0001
Socio-economic level (quintiles)			
SES 1 (lowest quintile)	1.5271	0.1069	<.0001
SES 2 (2nd lowest quintile)	1.3670	0.0975	<.0001
SES 3 (middle quintile)	1.2610	0.0912	0.0013
SES 4 (2nd highest quintile)	1.2159	0.0874	0.0066
SES 5 (highest quintile)	1	-	-

.

© 2023 Davies L et al. *JAMA Otolaryngology–Head & Neck Surgery*.

eTable 14. Probabilities of Death and Hazard Ratios of Dying for the Other Cause of Death Submodel in the Basic Model (Females)

**Reference Group (Baseline Hazards)**: Hispanic and Non-Hispanic Whites, Highest SES Quintile and Stage I. Attained Age	Probability of Death	Standard Error	p Value
Age 55	0.0158	0.0042	<.0001
Age 56	0.0096	0.0031	<.0001
Age 57	0.0096	0.0029	<.0001
Age 58	0.0084	0.0027	<.0001
Age 59	0.0080	0.0026	<.0001
Age 60	0.0078	0.0025	<.0001
Age 61	0.0108	0.0029	<.0001
Age 62	0.0105	0.0028	<.0001
Age 63	0.0111	0.0029	<.0001
Age 64	0.0073	0.0023	<.0001
Age 65	0.0177	0.0037	<.0001
Age 66	0.0122	0.0031	<.0001
Age 67	0.0104	0.0028	<.0001
Age 68	0.0167	0.0037	<.0001
Age 69	0.0180	0.0039	<.0001
Age 70	0.0253	0.0047	<.0001
Age 71	0.0159	0.0037	<.0001
Age 72	0.0198	0.0041	<.0001
Age 73	0.0309	0.0051	<.0001
Age 74	0.0275	0.0049	<.0001
Age 75	0.0202	0.0041	<.0001
Age 76	0.0323	0.0052	<.0001
Age 77	0.0327	0.0052	<.0001
Age 78	0.0309	0.0050	<.0001
Age 79	0.0383	0.0057	<.0001
Age 80	0.0437	0.0063	<.0001
Age 81	0.0390	0.0060	<.0001
Age 82	0.0345	0.0057	<.0001
Age 83	0.0382	0.0062	<.0001
Age 84	0.0331	0.0057	<.0001
Age 85	0.0513	0.0076	<.0001
Age 86	0.0578	0.0084	<.0001
Age 87	0.0571	0.0086	<.0001
Age 88	0.0713	0.0104	<.0001
Age 89	0.0708	0.0113	<.0001
Age 90	0.0770	0.0123	<.0001
Age 91	0.0770	0.0133	<.0001
Age 92	0.0675	0.0135	<.0001
Age 93	0.1116	0.0186	<.0001
Age 94	0.0911	0.0202	<.0001
Age 95	0.0923	0.0271	<.0001
Age 96	0.1485	0.0427	<.0001
Age 97	0.2718	0.0694	<.0001

© 2023 Davies L et al. *JAMA Otolaryngology–Head & Neck Surgery*.

eTable 14 (Cont.). Probabilities of death and Hazard Ratios of dying for the other cause of death sub-model in the Basic Model (Females).			
Covariates	Hazard Ratio	Standard Error	p Value
Race/Ethnicity			
Hispanic/NH White	1	-	-
NH Black	1.1883	0.1395	0.1418
NH API	0.5325	0.0786	<.0001
Stage			
Stage I	1	-	-
Stage II	1.1767	0.0860	0.0261
Stage III	1.4920	0.1183	<.0001
Stage IV	1.5212	0.1094	<.0001
Socio-economic level (quintiles)			
SES 1 (lowest quintile)	1.5020	0.1371	<.0001
SES 2/3 (2nd lowest or middle quintile)	1.3390	0.1044	0.0002
SES 4 (2nd highest quintile)	1.2173	0.1087	0.0277
SES 5 (highest quintile)	1	-	-

© 2023 Davies L et al. *JAMA Otolaryngology–Head & Neck Surgery*.

**eTable 15. Probabilities of Death and Hazard Ratios of Dying for the Other Cause of Death Submodel in the Coexisting Condition Model (Males)**			
**Reference Group (Baseline Hazards)**: Non-Hispanic Whites, Highest SES Quintile, Stage I, Age at Diagnosis 66 and Comorbidity Score 0. Attained Age	Probability of Death	Standard Error	p Value
Age 66	0.0287	0.0071	<.0001
Age 67	0.0237	0.0050	<.0001
Age 68	0.0276	0.0051	<.0001
Age 69	0.0279	0.0050	<.0001
Age 70	0.0245	0.0050	<.0001
Age 71	0.0314	0.0061	<.0001
Age 72	0.0256	0.0053	<.0001
Age 73	0.0407	0.0081	<.0001
Age 74	0.0390	0.0076	<.0001
Age 75	0.0474	0.0092	<.0001
Age 76	0.0504	0.0102	<.0001
Age 77	0.0527	0.0107	<.0001
Age 78	0.0419	0.0089	<.0001
Age 79	0.0452	0.0099	<.0001
Age 80	0.0433	0.0099	<.0001
Age 81	0.0595	0.0131	<.0001
Age 82	0.0533	0.0126	<.0001
Age 83	0.0802	0.0180	<.0001
Age 84	0.0846	0.0194	<.0001
Age 85	0.0852	0.0203	<.0001
Age 86	0.1060	0.0261	<.0001
Age 87	0.1016	0.0265	<.0001
Age 88	0.1384	0.0375	<.0001
Age 89	0.1385	0.0399	<.0001
Age 90	0.1285	0.0406	<.0001
Age 91	0.1991	0.0618	<.0001
Age 92	0.2724	0.0876	<.0001
Age 93	0.1541	0.0640	<.0001
Age 94	0.2876	0.1141	0.0017
Age 95	0.1677	0.1049	0.0043
Age 96	0.1212	0.1239	0.039
Age 97	0.3768	0.2637	0.163

© 2023 Davies L et al. *JAMA Otolaryngology–Head & Neck Surgery.*

**eTable 15 (Cont.). Probabilities of death and Hazard Ratios of dying for the other cause of death sub-model in the Coexisting Condition Model (Males).**			
Covariates	Hazard Ratio	Standard Error	p Value
Race/Ethnicity			
NH White	1	-	-
Hispanic	0.7071	0.0882	0.0055
NH Black	0.8388	0.1254	0.2397
NH API	0.7857	0.1024	0.064
Stage			
Stage I	1	-	-
Stage II	1.2964	0.0919	0.0003
Stage III	1.3259	0.1185	0.0016
Stage IV	1.3634	0.1039	<.0001
Socio-economic level (quintiles)			
SES 1/2/3/4 (not highest quintile)	1.1643	0.0838	0.0345
SES 5 (highest quintile)	1	-	-
Age at diagnosis			
Spline term 0	0.9537	0.0368	0.2192
Spline term 1	1.1855	0.1981	0.3086
Spline term 2	0.6486	0.2363	0.2347
Comorbidity score			
Spline term 0	1.0133	0.0036	0.0002
Spline term 1	1.1618	0.0847	0.0397
Spline term 2	0.7256	0.1527	0.1276

© 2023 Davies L et al. *JAMA Otolaryngology–Head & Neck Surgery.*

eTable 16. Probabilities of Death and Hazard Ratios of Dying for the Other Cause of Death Submodel in the Coexisting Condition Model (Females)

**Reference Group (Baseline Hazards):** Hispanics and Non-Hispanic Whites, Highest and Second Highest SES Quintiles, Stage I, Age at Diagnosis 66 and Comorbidity Score 0. Attained Age	Probability of Death	Standard Error	p Value
Age 66	0.0105	0.0046	<.0001
Age 67	0.0071	0.0029	<.0001
Age 68	0.0097	0.0038	<.0001
Age 69	0.0130	0.0043	<.0001
Age 70	0.0226	0.0056	<.0001
Age 71	0.0136	0.0041	<.0001
Age 72	0.0131	0.0040	<.0001
Age 73	0.0281	0.0072	<.0001
Age 74	0.0242	0.0068	<.0001
Age 75	0.0201	0.0062	<.0001
Age 76	0.0350	0.0099	<.0001
Age 77	0.0356	0.0103	<.0001
Age 78	0.0340	0.0099	<.0001
Age 79	0.0439	0.0128	<.0001
Age 80	0.0525	0.0153	<.0001
Age 81	0.0478	0.0144	<.0001
Age 82	0.0447	0.0140	<.0001
Age 83	0.0499	0.0157	<.0001
Age 84	0.0431	0.0139	<.0001
Age 85	0.0695	0.0217	<.0001
Age 86	0.0798	0.0251	<.0001
Age 87	0.0810	0.0257	<.0001
Age 88	0.1030	0.0329	<.0001
Age 89	0.1059	0.0346	<.0001
Age 90	0.1196	0.0398	<.0001
Age 91	0.1259	0.0433	<.0001
Age 92	0.1094	0.0404	<.0001
Age 93	0.1862	0.0667	<.0001
Age 94	0.1620	0.0625	<.0001
Age 95	0.1631	0.0706	<.0001
Age 96	0.2946	0.1177	0.0022
Age 97	0.5688	0.2350	0.172

© 2023 Davies L et al. *JAMA Otolaryngology–Head & Neck Surgery.*

eTable 16 (Cont.). Probabilities of death and Hazard Ratios of dying for the other cause of death sub-model in the Coexisting Condition Model (Females).			
Covariates	Hazard Ratio	Standard Error	p Value
Race/Ethnicity			
Hispanic/NH White	1	-	-
NH API	0.5439	0.0869	0.0001
NH Black	1.6807	0.5099	0.087
NH Black and Attained Age greater than 73	0.5577	0.1823	0.0741
Stage			
Stage I	1	-	-
Stage II	1.1460	0.0896	0.0812
Stage III/IV	1.3679	0.0925	<.0001
Socio-economic level (quintiles)			
SES 1/3 (lowest quintile or middle quintile)	1.2265	0.0752	0.0009
SES 2 (2nd lowest quintile) and Attained Age less than 70*	2.0980	0.7188	0.0306
SES 4/5 (highest or 2nd highest quintiles)	1	-	-
Age at diagnosis			
Spline term 0	0.9772	0.0430	0.6
Spline term 1	0.9923	0.1318	0.954
Spline term 2	1.0482	0.3588	0.8905
Comorbidity score			
Spline term 0	1.0316	0.0167	0.0554
Spline term 1	1.4236	0.5045	0.3189
Spline term 2	0.4619	0.3830	0.3515
Age at diagnosis * Comorbidity score			
Age Spline term 0 * Comorbidity Spline term 0	0.9969	0.0033	0.3449
Age Spline term 0 * Comorbidity Spline term 1	1.0199	0.0742	0.7865
Age Spline term 0 * Comorbidity Spline term 2	0.9588	0.1647	0.8065
Age Spline term 1 * Comorbidity Spline term 0	1.0078	0.0110	0.4763
Age Spline term 1 * Comorbidity Spline term 1	0.8931	0.2126	0.6347
Age Spline term 1 * Comorbidity Spline term 2	1.2881	0.7257	0.6531
Age Spline term 2 * Comorbidity Spline term 0	0.9786	0.0282	0.4536
Age Spline term 2 * Comorbidity Spline term 1	1.4061	0.8777	0.585
Age Spline term 2 * Comorbidity Spline term 2	0.4645	0.6862	0.6037

*The group of SES 2 (the second lowest SES quintile) on and after attained age 70 is collapsed with the group of SES 4/5 (the highest and second highest SES quintiles).

© 2023 Davies L et al. *JAMA Otolaryngology–Head & Neck Surgery.*

eTable 17. Probabilities of Death and Hazard Ratios of Dying for the Other Cause of Death Submodel in the General Health Self-Assessment Model (Males)

**Reference Group (Baseline Hazards)**: Non-Hispanic Whites, Never Smokers, Year of Diagnosis 1986-1993, Age at Survey 40 and Excellent Health Status. Attained Age	Probability of Death	Standard Error	p Value
Age 40	0.0012	0.0002	<.0001
Age 41	0.0011	0.0001	<.0001
Age 42	0.0014	0.0002	<.0001
Age 43	0.0016	0.0002	<.0001
Age 44	0.0013	0.0001	<.0001
Age 45	0.0016	0.0001	<.0001
Age 46	0.0017	0.0001	<.0001
Age 47	0.0015	0.0001	<.0001
Age 48	0.0018	0.0002	<.0001
Age 49	0.0024	0.0002	<.0001
Age 50	0.0023	0.0002	<.0001
Age 51	0.0030	0.0002	<.0001
Age 52	0.0028	0.0002	<.0001
Age 53	0.0029	0.0002	<.0001
Age 54	0.0029	0.0002	<.0001
Age 55	0.0032	0.0002	<.0001
Age 56	0.0034	0.0002	<.0001
Age 57	0.0033	0.0002	<.0001
Age 58	0.0040	0.0003	<.0001
Age 59	0.0038	0.0002	<.0001
Age 60	0.0042	0.0003	<.0001
Age 61	0.0045	0.0003	<.0001
Age 62	0.0045	0.0003	<.0001
Age 63	0.0046	0.0003	<.0001
Age 64	0.0054	0.0003	<.0001
Age 65	0.0058	0.0004	<.0001
Age 66	0.0060	0.0004	<.0001
Age 67	0.0068	0.0004	<.0001
Age 68	0.0072	0.0005	<.0001
Age 69	0.0075	0.0005	<.0001
Age 70	0.0083	0.0005	<.0001
Age 71	0.0093	0.0006	<.0001
Age 72	0.0094	0.0006	<.0001
Age 73	0.0106	0.0006	<.0001
Age 74	0.0113	0.0007	<.0001
Age 75	0.0119	0.0007	<.0001
Age 76	0.0134	0.0008	<.0001
Age 77	0.0135	0.0009	<.0001
Age 78	0.0146	0.0009	<.0001
Age 79	0.0158	0.0010	<.0001
Age 80	0.0179	0.0011	<.0001
Age 81	0.0195	0.0012	<.0001

© 2023 Davies L et al. *JAMA Otolaryngology–Head & Neck Surgery.*

eTable 17 (Cont.). Probabilities of death and Hazard Ratios of dying for the other cause of death sub-model in the General Health Self-Assessment Model (Males).			
Attained Age (Cont.)	Probability of Death	Standard Error	p Value
Age 82	0.0206	0.0012	<.0001
Age 83	0.0235	0.0014	<.0001
Age 84	0.0250	0.0015	<.0001
Age 85	0.0266	0.0016	<.0001
Age 86	0.0281	0.0018	<.0001
Age 87	0.0324	0.0021	<.0001
Age 88	0.0335	0.0021	<.0001
Age 89	0.0387	0.0026	<.0001
Age 90	0.0428	0.0028	<.0001
Age 91	0.0432	0.0031	<.0001
Age 92	0.0446	0.0033	<.0001
Age 93	0.0499	0.0039	<.0001
Age 94	0.0468	0.0039	<.0001
Age 95	0.0491	0.0043	<.0001

Covariates	Hazard Ratio	Standard Error	p Value
Race/Ethnicity			
NH White	1	-	-
Hispanic	0.7824	0.0199	<.0001
NH Black	1.4545	0.0739	<.0001
NH API	0.6569	0.0375	<.0001
Survey Year			
1986-1993	1	-	-
1994-2001	0.9211	0.0121	<.0001
2002-2009	0.7400	0.0135	<.0001
Smoking Status			
Current	2.1242	0.0344	<.0001
Former	1.2647	0.0168	<.0001
Never	1	-	-
Age at Survey			
Spline term 0	0.9974	0.0103	0.8030
Spline term 1	1.1100	0.0504	0.0218
Spline term 2	0.8237	0.0742	0.0315
Health Status			
Excellent	1	-	-
Very Good	1.3370	0.0718	<.0001
Good	1.9626	0.1011	<.0001
Fair	3.7075	0.2428	<.0001
Poor	6.9603	0.5909	<.0001

© 2023 Davies L et al. *JAMA Otolaryngology–Head & Neck Surgery.*

eTable 17 (Cont.). Probabilities of death and Hazard Ratios of dying for the other cause of death sub-model in the General Health Self-Assessment Model (Males).			
Covariates	Hazard Ratio	Standard Error	p Value
Age at Survey * Health Status			
Age Spline 0 * Very Good	0.9995	0.0138	0.9714
Age Spline 1 * Very Good	0.9849	0.0598	0.8021
Age Spline 2 * Very Good	1.0291	0.1231	0.8107
Age Spline 0 * Good	1.0040	0.0133	0.7642
Age Spline 1 * Good	0.9601	0.0553	0.4803
Age Spline 2 * Good	1.0688	0.1208	0.5562
Age Spline 0 * Fair	1.0081	0.0170	0.6312
Age Spline 1 * Fair	0.9162	0.0656	0.2216
Age Spline 2 * Fair	1.1592	0.1622	0.2911
Age Spline 0 * Poor	1.0106	0.0220	0.6301
Age Spline 1 * Poor	0.8725	0.0779	0.1271
Age Spline 2 * Poor	1.2870	0.2222	0.1442
NH Black * Health Status			
NH Black * Very Good	0.8209	0.0561	0.0040
NH Black * Good	0.8130	0.0462	0.0003
NH Black * Fair	0.6559	0.0416	<.0001
NH Black * Poor	0.6291	0.0464	<.0001

© 2023 Davies L et al. *JAMA Otolaryngology–Head & Neck Surgery*.

eTable 18. Probabilities of Death and Hazard Ratios of Dying for the Other Cause of Death Submodel in the General Health Self-Assessment Model (Females)

**Reference Group (Baseline Hazards)**: Non-Hispanic Whites, Never Smokers, Year of Diagnosis 1986-1993, Age at Survey 40 and Excellent Health Status.<br>Attained Age	Probability of Death	Standard Error	p Value
Age 40	0.0008	0.0001	<.0001
Age 41	0.0007	0.0001	<.0001
Age 42	0.0008	0.0001	<.0001
Age 43	0.0008	0.0001	<.0001
Age 44	0.0008	0.0001	<.0001
Age 45	0.0011	0.0001	<.0001
Age 46	0.0012	0.0001	<.0001
Age 47	0.0010	0.0001	<.0001
Age 48	0.0010	0.0001	<.0001
Age 49	0.0013	0.0001	<.0001
Age 50	0.0013	0.0001	<.0001
Age 51	0.0017	0.0001	<.0001
Age 52	0.0018	0.0001	<.0001
Age 53	0.0019	0.0002	<.0001
Age 54	0.0018	0.0001	<.0001
Age 55	0.0020	0.0002	<.0001
Age 56	0.0021	0.0002	<.0001
Age 57	0.0022	0.0002	<.0001
Age 58	0.0024	0.0002	<.0001
Age 59	0.0026	0.0002	<.0001
Age 60	0.0027	0.0002	<.0001
Age 61	0.0026	0.0002	<.0001
Age 62	0.0030	0.0002	<.0001
Age 63	0.0032	0.0002	<.0001
Age 64	0.0035	0.0003	<.0001
Age 65	0.0036	0.0003	<.0001
Age 66	0.0040	0.0003	<.0001
Age 67	0.0041	0.0003	<.0001
Age 68	0.0046	0.0003	<.0001
Age 69	0.0048	0.0003	<.0001
Age 70	0.0056	0.0004	<.0001
Age 71	0.0056	0.0004	<.0001
Age 72	0.0064	0.0004	<.0001
Age 73	0.0065	0.0005	<.0001
Age 74	0.0073	0.0005	<.0001
Age 75	0.0086	0.0006	<.0001
Age 76	0.0094	0.0006	<.0001
Age 77	0.0101	0.0007	<.0001
Age 78	0.0111	0.0008	<.0001
Age 79	0.0126	0.0009	<.0001

© 2023 Davies L et al. *JAMA Otolaryngology–Head & Neck Surgery*.

eTable 18 (Cont.). Probabilities of death and Hazard Ratios of dying for the other cause of death sub-model in the General Health Self-Assessment Model (Females).			
Attained Age (Cont.)	Probability of Death	Standard Error	p Value
Age 80	0.0136	0.0009	<.0001
Age 81	0.0148	0.0010	<.0001
Age 82	0.0165	0.0011	<.0001
Age 83	0.0184	0.0012	<.0001
Age 84	0.0206	0.0014	<.0001
Age 85	0.0232	0.0016	<.0001
Age 86	0.0261	0.0018	<.0001
Age 87	0.0274	0.0019	<.0001
Age 88	0.0304	0.0021	<.0001
Age 89	0.0356	0.0024	<.0001
Age 90	0.0390	0.0027	<.0001
Age 91	0.0428	0.0030	<.0001
Age 92	0.0475	0.0034	<.0001
Age 93	0.0464	0.0036	<.0001
Age 94	0.0529	0.0040	<.0001
Age 95	0.0553	0.0042	<.0001

Covariates	Hazard Ratio	Standard Error	p Value
Race/Ethnicity			
NH White	1	-	-
Hispanic	0.7557	0.0176	<.0001
Race/Ethnicity (Cont.)	Hazard Ratio	Standard Error	p Value
NH Black	1.3722	0.0686	<.0001
NH API	0.6472	0.0421	<.0001
Survey Year			
1986-1993	1	-	-
1994-2001	0.9330	0.0116	<.0001
2002-2009	0.7533	0.0135	<.0001
Smoking Status			
Current	2.1112	0.0313	<.0001
Former	1.3310	0.0169	<.0001
Never	1	-	-
Age at Survey			
Spline term 0	0.9850	0.0103	0.1493
Spline term 1	1.1985	0.0505	<.0001
Spline term 2	0.6919	0.0566	<.0001

© 2023 Davies L et al. *JAMA Otolaryngology–Head & Neck Surgery.*

eTable 18 (Cont.). Probabilities of death and Hazard Ratios of dying for the other cause of death sub-model in the General Health Self-Assessment Model (Females).			
. Covariates	Hazard Ratio	Standard Error	p Value
Health Status			
Excellent	-	-	-
Very Good	1.2995	0.0771	<.0001
Good	2.1225	0.1240	<.0001
Fair	4.1625	0.2866	<.0001
Poor	7.5669	0.6471	<.0001
Age at Survey * Health Status			
Age Spline 0 * Very Good	0.9919	0.0137	0.5544
Age Spline 1 * Very Good	1.0129	0.0563	0.8175
Age Spline 2 * Very Good	0.9787	0.1052	0.8409
Age Spline 0 * Good	1.0112	0.0131	0.3896
Age Spline 1 * Good	0.8984	0.0462	0.0376
Age Spline 2 * Good	1.2336	0.1222	0.0343
Age Spline 0 * Fair	1.0192	0.0149	0.1932
Age Spline 1 * Fair	0.8378	0.0489	0.0025
Age Spline 2 * Fair	1.4028	0.1584	0.0028
Age Spline 0 * Poor	1.0490	0.0194	0.0098
Age Spline 1 * Poor	0.7475	0.0530	<.0001
Age Spline 2 * Poor	1.7022	0.2314	<.0001
NH Black * Health Status			
NH Black * Very Good	0.8480	0.0545	0.0105
NH Black * Good	0.7780	0.0457	<.0001
NH Black * Fair	0.7163	0.0416	<.0001
NH Black * Poor	0.6196	0.0413	<.0001

© 2023 Davies L et al. *JAMA Otolaryngology–Head & Neck Surgery.*

eAppendix 5. Baseline Hazards for the Other Cause of Death Submodels

A Gompertz model was fit to the smooth and extrapolate the base-line hazards by sex for the Coexisting Condition Model and the General Health Self-Assessment Model other cause of death sub-models. This was done to facilitate the calculation of LE in the absence of cancer using the covariate profile for a patient. eFigures 3 through 6 present the graphs of the extrapolations of the baseline hazards to age 110 by sex for these two sub-models.

For the Basic Model other cause of death sub-models, we did not use the Gompertz model to smooth and extrapolate the baseline hazards for the purpose of calculating LE, partly because US lifetable do not have a baseline. Connected to this is the problem of dealing with the connection between the use of US life tables (used because of the paucity of deaths between ages 20 and 54) and the modeling using SEER data for those aged 55-86. The life tables only accounted for age, sex, and race/ethnicity for probabilities of dying from other causes in the age range 20-54, whereas the probabilities of dying from other causes starting at age 55 are much more specific as they take into account that a person has oral cancer, their stage of disease, the SES quintile of the census tract in which they live, in addition to age, sex, and race/ethnicity. eFigures 7 and 8 provide the interval probabilities of death based on the baseline hazards for males and females respectively for ages 55-86 that were used in calculating LE using these two sub-models based on the covariate profile for a patient.

© 2023 Davies L et al. *JAMA Otolaryngology–Head & Neck Surgery.*

eFigure 3. Fit of Gompertz Model to Baseline Hazards for Other Cause of Death Submodel for Coexisting Condition Model (Males)

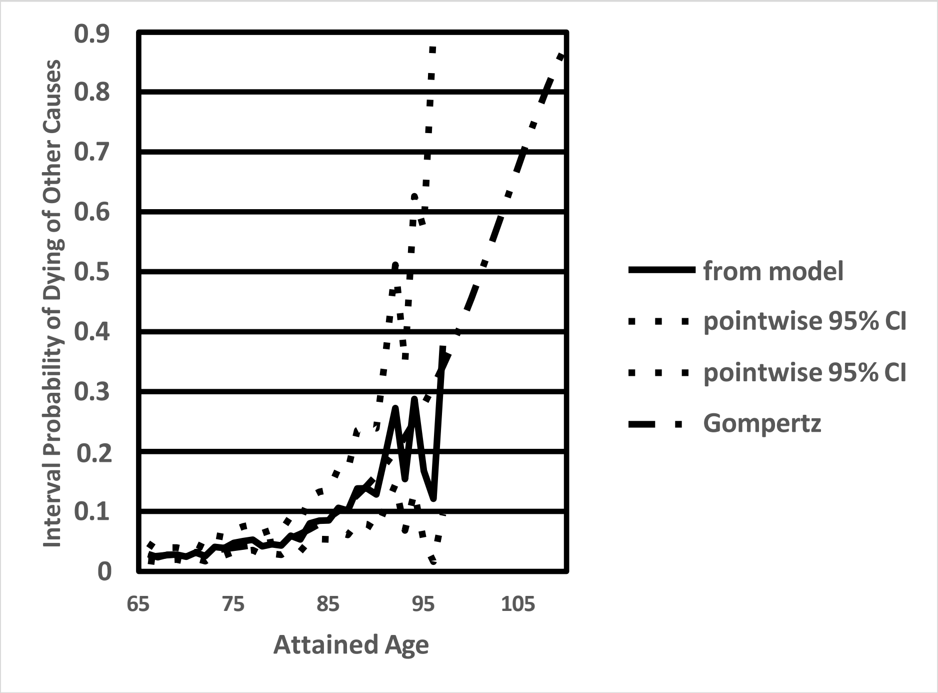

© 2023 Davies L et al. *JAMA Otolaryngology–Head & Neck Surgery*.

eFigure 4. Fit of Gompertz model to Baseline Hazards for Other Cause of Death Submodel for Coexisting Condition Model (Females)

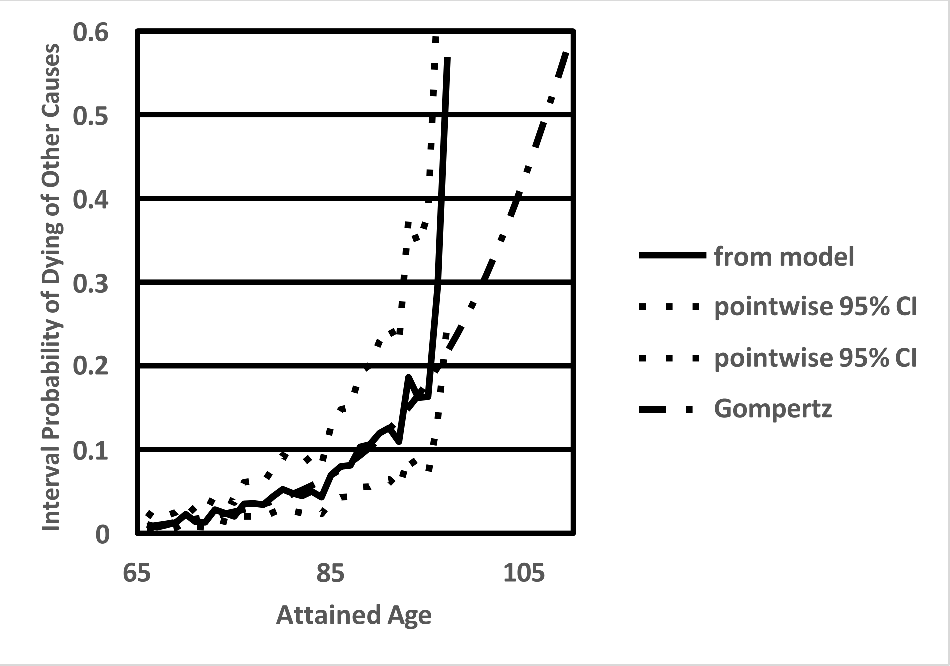

© 2023 Davies L et al. *JAMA Otolaryngology–Head & Neck Surgery*.

eFigure 5. Fit of Gompertz Model to Baseline Hazards for Other Cause of Death Submodel (for General Health Self-Assessment Model (Males)

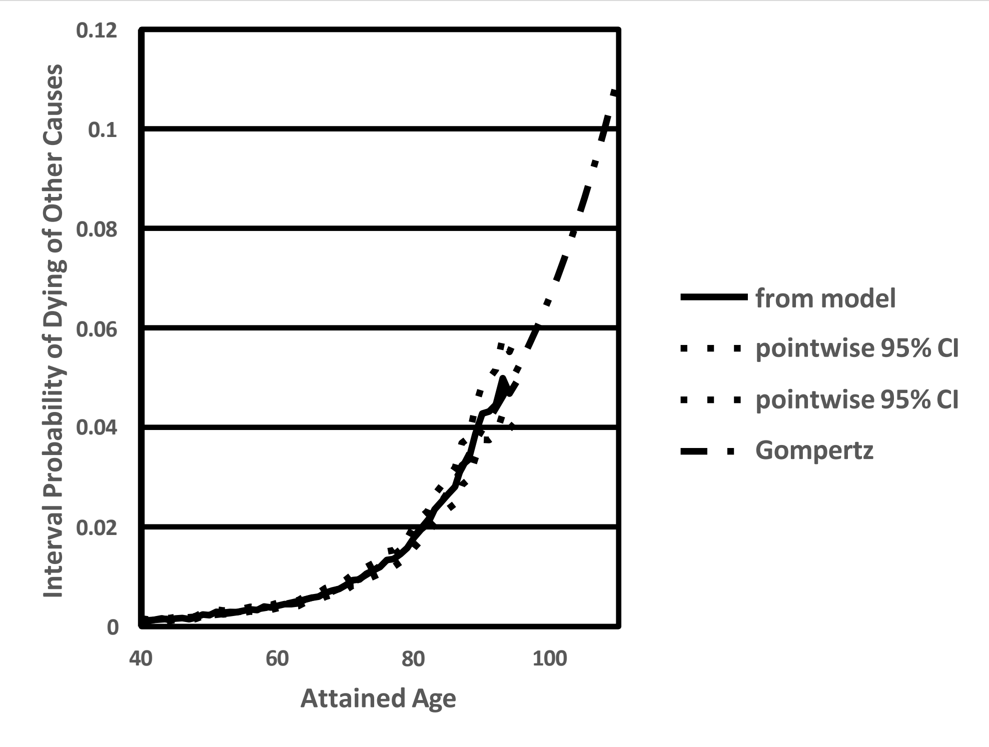

© 2023 Davies L et al. *JAMA Otolaryngology–Head & Neck Surgery*.

eFigure 6. Fit of Gompertz Model to Baseline Hazards for Other Cause of Death Submodel for General Health Self-Assessment Model (Females)

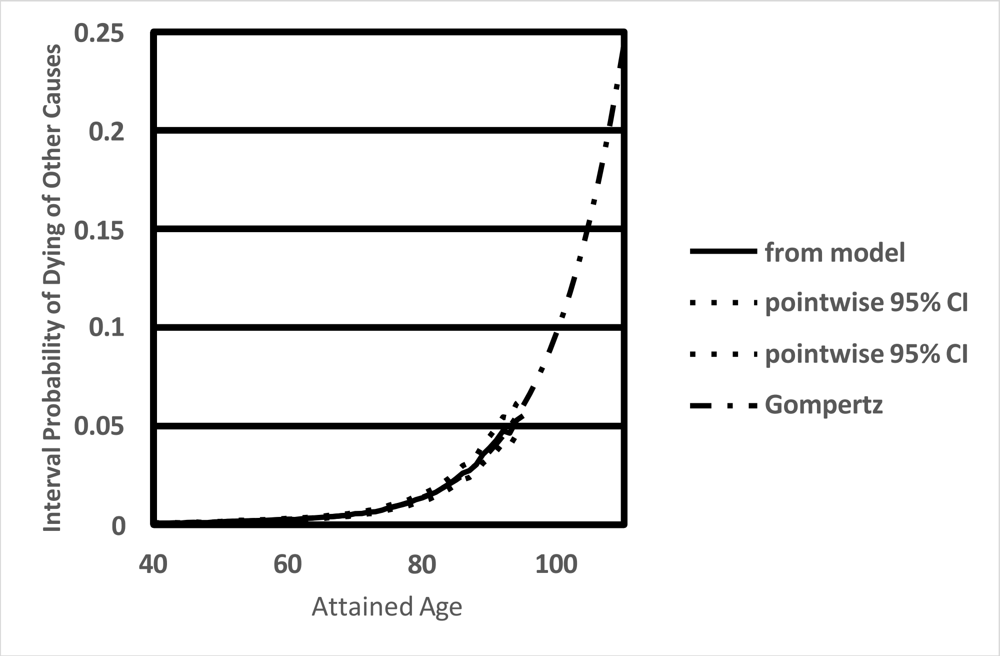

© 2023 Davies L et al. *JAMA Otolaryngology–Head & Neck Surgery*.

eFigure 7. Baseline Hazards of Death From Other Causes by Age for the Other Cause of Death Submodel for Basic Model (Males)

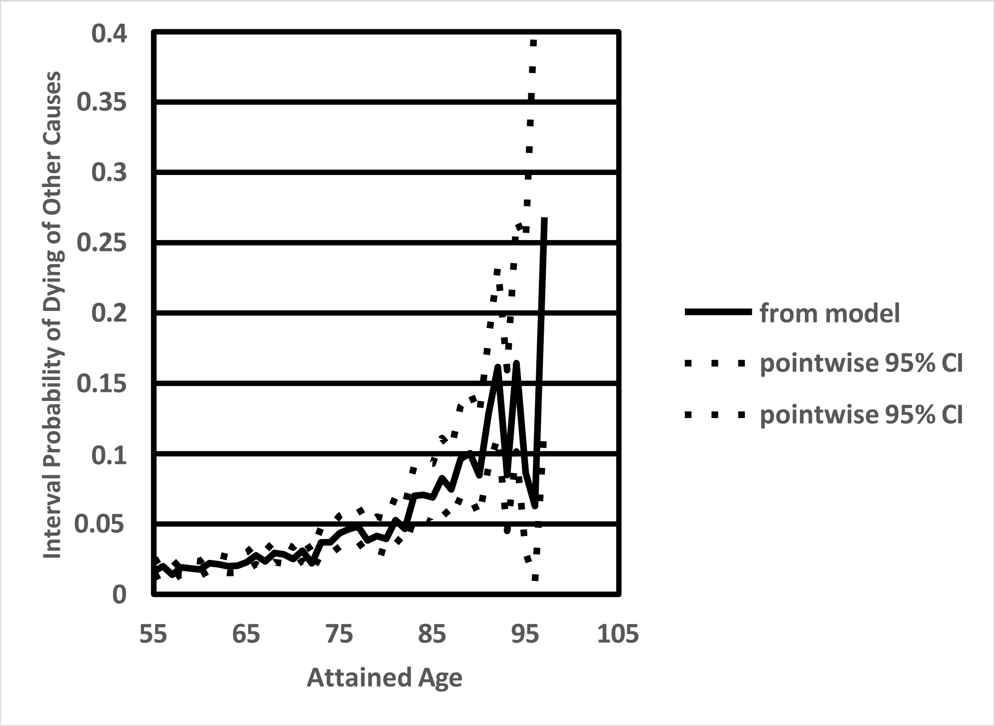

© 2023 Davies L et al. *JAMA Otolaryngology–Head & Neck Surgery*.

eFigure 8. Baseline Hazards of Death From Other Causes by Age for the Other Cause of Death Submodel for Basic Model (Females)

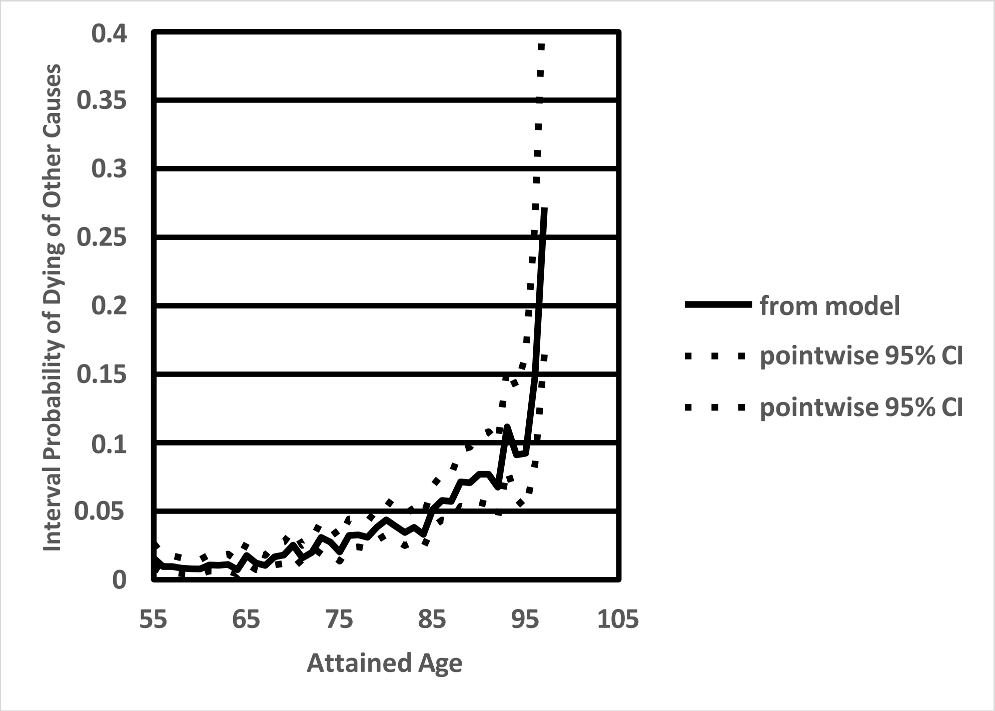

© 2023 Davies L et al. *JAMA Otolaryngology–Head & Neck Surgery*.

eAppendix 6. Validation

To investigate how well models for cancer death and for other-cause death predict cancer and other-cause mortality, we used calibration plots and time-dependent area under the ROC curve (AUC(t)) based on a 10-fold cross validation as described in Lee et al.[16] We computed the predicted cumulative incidence functions for both death from oral cancer and for death from other causes. To produce the calibration plots for death from oral cancer and death from other causes patients were divided into four groups using quartiles of the predicted cumulative incidence functions for death from cancer by a specific time t. For each quartile group, the predicted risk of death from cancer was compared to an estimate based on the nonparametric estimate[17] of the cumulative incidence function for death from oral cancer or to other causes by time t, as described in Gerds et al.[18]

A standard 10-fold cross validation method was applied to the Basic and Coexisting Conditions Models. For these two models, the validation was conducted on the final system implementation of the models, including estimates of other causes of death from the lifetable substituted for the estimates derived from fitting the statistical models. For the General Health Self-Assessment Model, a modified 10-fold cross validation method had to be used because of the complex survey design of the National Health Interview Survey.[13] Primary Sampling Units (PSU) instead of participants are randomly assigned into 10 groups and one group of PSUs is marked as dropped off for each iteration, with weights adjusted to reflect the status of PSUs within strata for the current iteration. For the Basic and Coexisting Conditions Models, since SEER contains information of both death due to oral cancer and death due to other causes, the predicted cumulative crude mortality from oral cancer death and from causes other than oral cancer were compared to the observed cumulative crude mortality from oral cancer death and from causes other than oral cancer death, respectively, overall and by stage and by sex (eFigures 9 to 15) for the Basic Model and eFigures 16 to 22 for the Co-existing Condition Model). For the General Health Self-Assessment Model, since NHIS represents the general population and death due to all causes is used as a substitute for death due non-oral cancer death the predicted cumulative net mortality from all causes was compared to the observed cumulative net mortality (eFigures 23 and 24 for males and females respectively).

For time-dependent AUC(t), we divide patients into cases and controls according to their vital status by time t and compare the predicted cumulative incidence functions for death from cancer (or death from other causes) by time t in patients who died of cancer (or died of other causes) by time t (cases) with those who did not by time t (controls), where censored individuals before time t were treated as controls. The time-dependent area under the curve (AUC) statistics[16] were calculated to assess the ability of the entire sub-models (including the US Life Tables combined with the SEER model utilized in the Basic Model for ages 20-54 and 55-86 respectively) to accurately predict outcomes. The AUC statistics, which are a measure of how well the models predict outcomes, are given for the Basic Model and Co-Existing Condition Model overall and by stage and sex, (eTables 19 and 20 respectively), and for the General Health Assessment Model by sex and age group in eTable 21.

The predicted calibration curves were very well matched to the observed curves, except for some moderate underestimation of death from other causes for those in the most favorable quartiles (eFigures 9 to 15) when using the Basic Model, probably due to having only a few available covariates. This same observation was noted to a lesser extent for the Coexisting Condition Model (eFigures 16 to 22). For the General Health Self-Assessment Model, the predicted cumulative net mortality from all causes was compared to the observed cumulative net mortality by sex and the fit was extremely good (eFigures 23 and 24).

The AUC for the Basic and Coexisting Conditions Models ranged from 0.834 to 0.635 for oral cancer death and from 0.748 down to 0.570 for death due to other causes. AUCs are typically larger in more heterogenous populations since it is easier to discriminate survival times among more heterogenous populations. The AUCs for death from cancer were therefore larger among all stages than among stage specific groups. The AUCs for other causes of death were generally smaller for later stages of disease and later time from diagnosis. In both situations there is significant filtering because the included cases have a high risk of death from cancer, which makes prediction of other causes of death difficult. The AUC for all causes of death from the General Health Self-Assessment Model ranged from 0.840 to 0.814 for men and women, and from 0.794 to 0.633 by sex and age group.

© 2023 Davies L et al. *JAMA Otolaryngology–Head & Neck Surgery.*

eFigure 9. Validation of Basic Model for ages 20-86, All Stages, Based on Observed Versus Predicted Cumulative Mortality Curves for the Cancer Death Submodel and Other Cause of Death Submodel. Patients divided into quartiles in each graph based on their predicted 9-year probabilities of dying of other causes or of cancer.

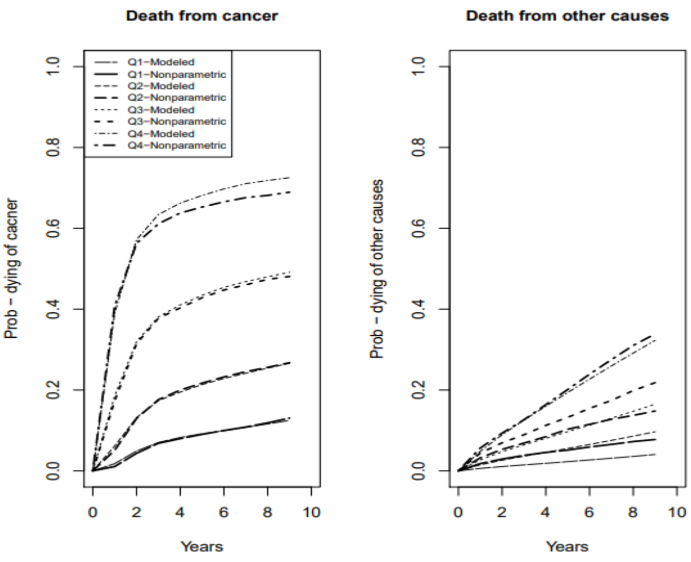

© 2023 Davies L et al. *JAMA Otolaryngology–Head & Neck Surgery*.

eFigure 10. Validation of Basic Model for Ages 20-86, Stage I, Based on Observed Versus Predicted Cumulative Mortality Curves for the Cancer Death Submodel and Other Cause of Death Submodel. Patients divided into quartiles in each graph based on their predicted 9-year probabilities of dying of other causes or of cancer.

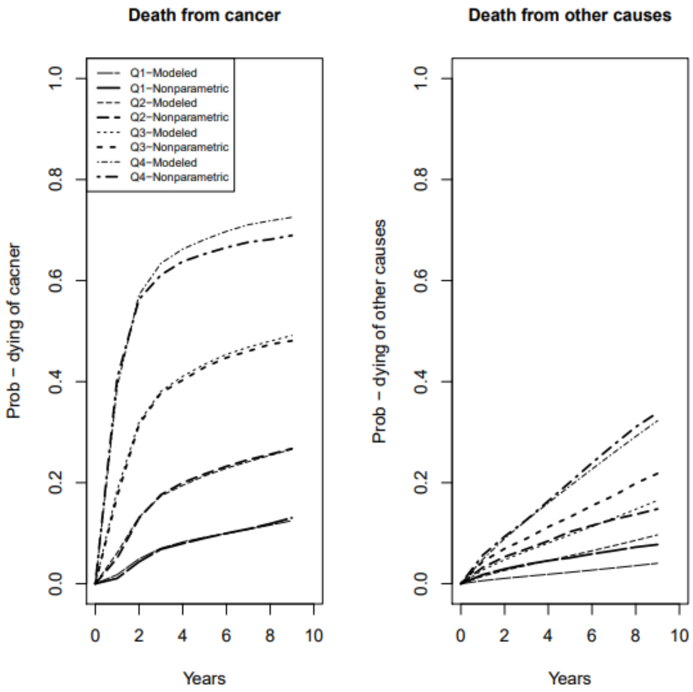

© 2023 Davies L et al. *JAMA Otolaryngology–Head & Neck Surgery*.

eFigure 11. Validation of Basic Model for Ages 20-86, Stage II, Based on Observed Versus Predicted Cumulative Mortality Curves for the Cancer Death Submodel and Other Cause of Death Submodel. Patients divided into quartiles in each graph based on their predicted 9-year probabilities of dying of other causes or of cancer.

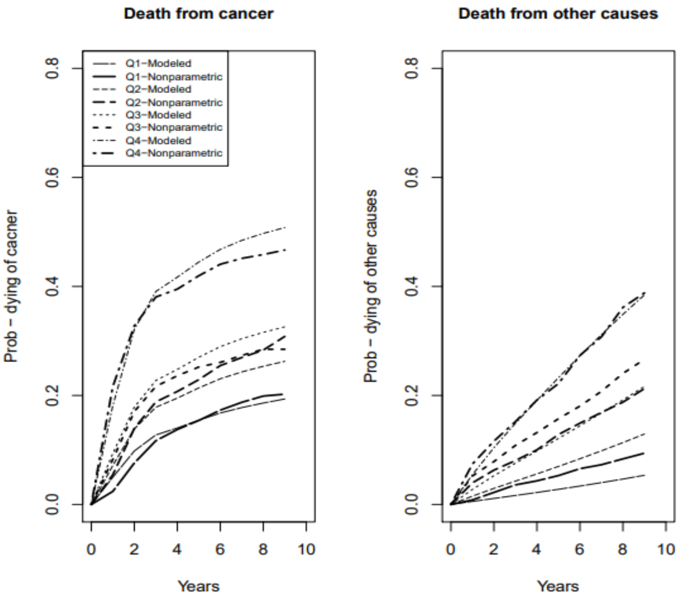

© 2023 Davies L et al. *JAMA Otolaryngology–Head & Neck Surgery*.

eFigure 12. Validation of Basic Model for Ages 20-86, Stage III, Based on Observed Versus Predicted Cumulative Mortality Curves for the Cancer Death Submodel and Other Cause of Death Submodel. Patients divided into quartiles in each graph based on their predicted 9-year probabilities of dying of other causes or of cancer.

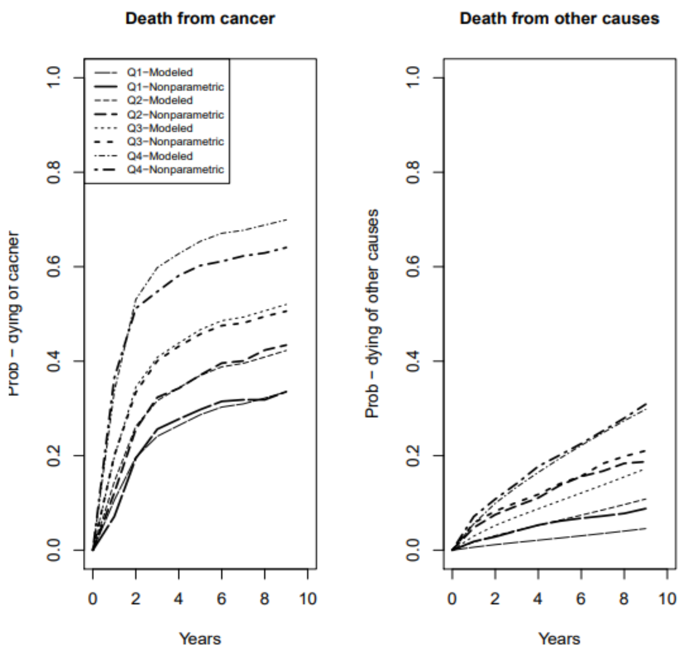

© 2023 Davies L et al. *JAMA Otolaryngology–Head & Neck Surgery.*

eFigure 13. Validation of Basic Model for Ages 20-86, Stage IV, Based on Observed Versus Predicted Cumulative Mortality Curves for the Cancer Death Submodel and Other Cause of Death Submodel. Patients divided into quartiles in each graph based on their predicted 9-year probabilities of dying of other causes or of cancer.

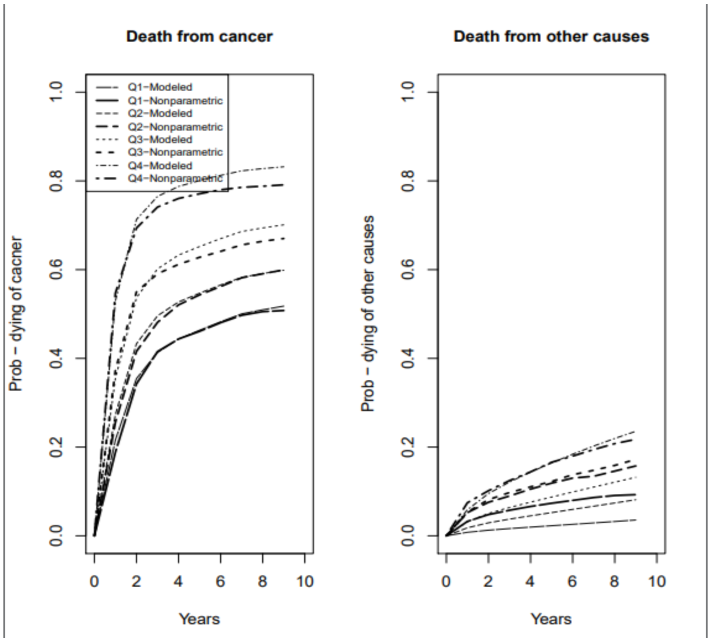

© 2023 Davies L et al. *JAMA Otolaryngology–Head & Neck Surgery*.

eFigure 14. Validation of Basic Model for Ages 20-86, Males, Based on Observed Versus Predicted Cumulative Mortality Curves for the Cancer Death Submodel and Other Cause of Death Submodel. Patients divided into quartiles in each graph based on their predicted 9-year probabilities of dying of other causes or of Cancer.

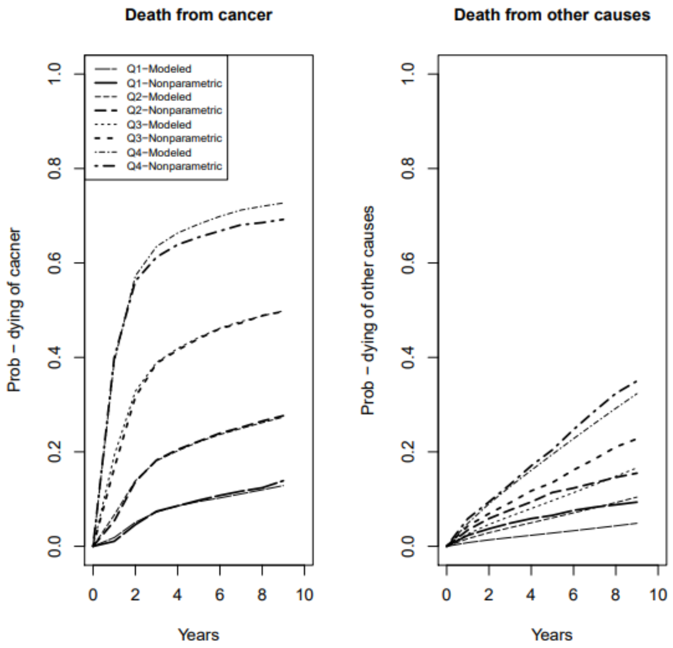

© 2023 Davies L et al. *JAMA Otolaryngology–Head & Neck Surgery*.

eFigure 15. Validation of Basic Model for Ages 20-86, Females, Based on Observed Versus Predicted Cumulative Mortality Curves for the Cancer Death Submodel and Other Cause of Death Submodel. Patients divided into quartiles in each graph based on their predicted 9-year probabilities of dying of other causes or of cancer.

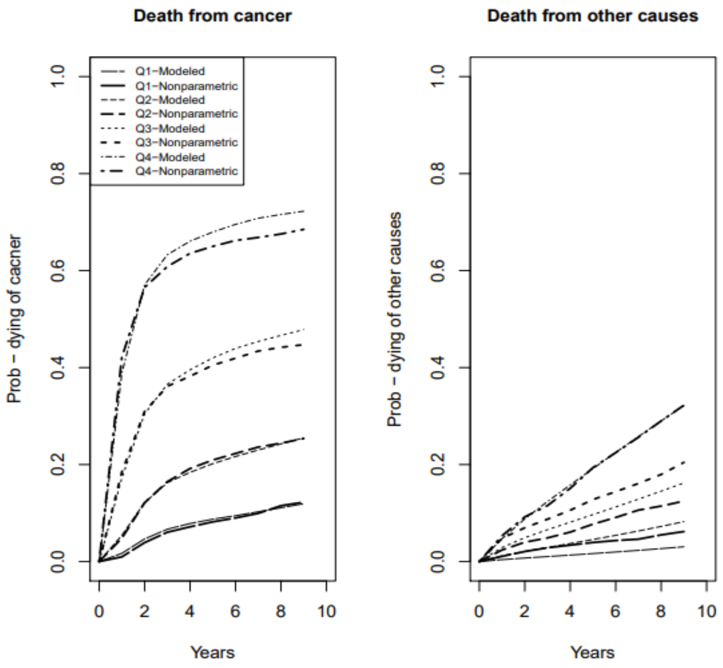

© 2023 Davies L et al. *JAMA Otolaryngology–Head & Neck Surgery*.

eTable 19. Area Under the Curve (AUC) Statistics for the Cancer Death Submodel (AUC1) and the Other Cause of Death Submodel (AUC2) of the Basic Model (Ages 20-86) by Stage and Sex.

Oral Cavity - All Stages, 21,437 patients	AUC1	AUC2
1-year	0.828	0.708
3-year	0.788	0.662
5-year	0.781	0.661
7-year	0.777	0.665
9-year	0.773	0.667

Oral Cavity - Males, 13,246 patients	AUC1	AUC2
1-year	0.825	0.693
3-year	0.783	0.645
5-year	0.778	0.642
7-year	0.774	0.650
9-year	0.769	0.654

Oral Cavity - Females, 8,191 patients	AUC1	AUC2
1-year	0.834	0.732
3-year	0.794	0.686
5-year	0.786	0.688
7-year	0.782	0.688
9-year	0.778	0.686

Oral Cavity - Stage 1, 7,689 patients	AUC1	AUC2
1-year	0.763	0.748
3-year	0.682	0.708
5-year	0.687	0.716
7-year	0.689	0.720
9-year	0.686	0.718

Oral Cavity - Stage 2, 3,829 patients	AUC1	AUC2
1-year	0.739	0.728
3-year	0.663	0.680
5-year	0.653	0.669
7-year	0.648	0.673
9-year	0.646	0.673

Oral Cavity - Stage 3, 3,259 patients	AUC1	AUC2
1-year	0.718	0.687
3-year	0.640	0.647
5-year	0.642	0.635
7-year	0.642	0.633
9-year	0.645	0.631

Oral Cavity - Stage 4, 6,660 patients	AUC1	AUC2
1-year	0.683	0.637
3-year	0.651	0.603
5-year	0.650	0.600
7-year	0.648	0.596
9-year	0.648	0.593

© 2023 Davies L et al. *JAMA Otolaryngology–Head & Neck Surgery*.

eFigure 16. Validation of Coexisting Condition Model for Ages 66-86, All Stages, Based on Observed Versus Predicted Cumulative Mortality Curves for the Cancer Death Submodel and Other Cause of Death Submodel. Patients divided into quartiles in each graph based on their predicted 9-year probabilities of dying of other causes or of cancer.

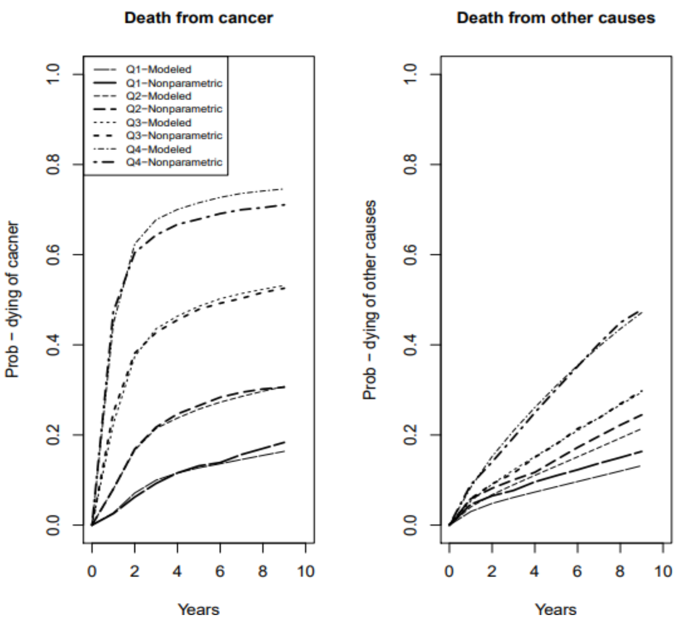

© 2023 Davies L et al. *JAMA Otolaryngology–Head & Neck Surgery*.

eFigure 17. Validation of Coexisting Condition Model for Ages 66-86, Stage I, Based on Observed Versus Predicted Cumulative Mortality Curves for the Cancer Death Submodel and Other Cause of Death Submodel. Patients divided into quartiles in each graph based on their predicted 9-year probabilities of dying of other causes or of cancer.

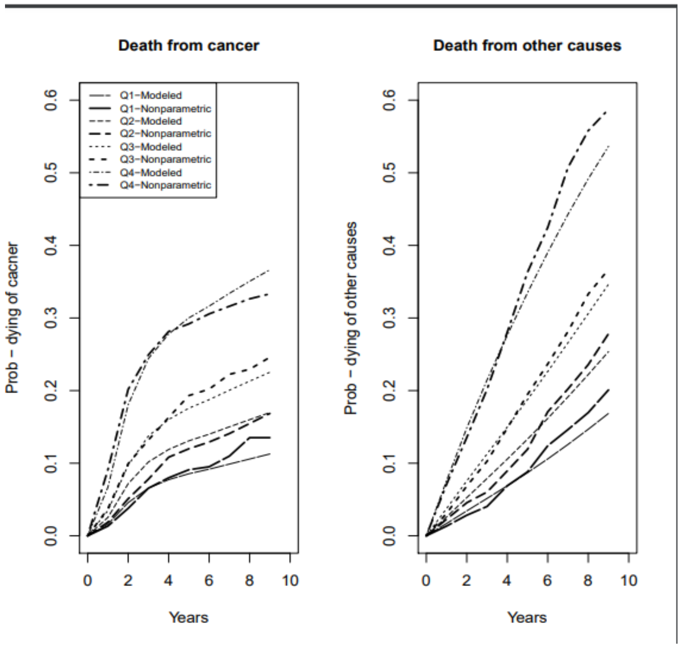

© 2023 Davies L et al. *JAMA Otolaryngology–Head & Neck Surgery*.

eFigure 18. Validation of Coexisting Condition Model for Ages 66-86, Stage II, Based on Observed Versus Predicted Cumulative Mortality Curves for the Cancer Death Submodel and Other Cause of Death Submodel. Patients divided into quartiles in each graph based on their predicted 9-year probabilities of dying of other causes or of cancer.

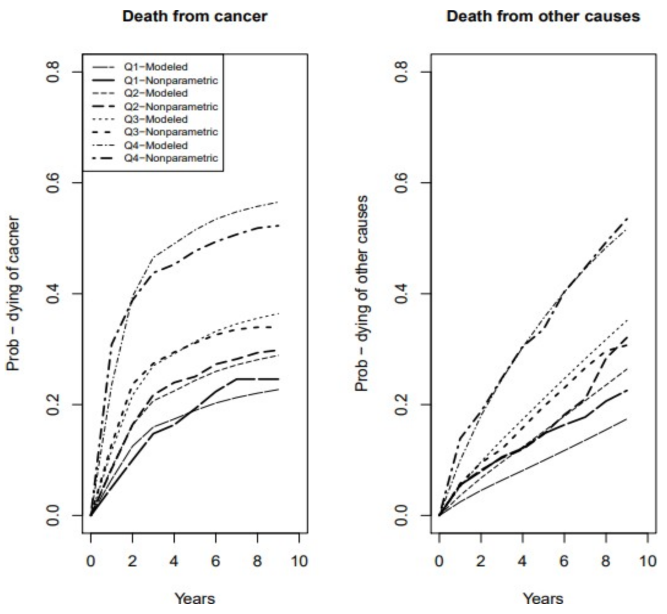

© 2023 Davies L et al. *JAMA Otolaryngology–Head & Neck Surgery.*

eFigure 19. Validation of Coexisting Condition Model for Ages 66-86, Stage III, Based on Observed Versus Predicted Cumulative Mortality Curves for the Cancer Death Submodel and Other Cause of Death Submodel. Patients divided into quartiles in each graph based on their predicted 9-year probabilities of dying of other causes or of cancer.

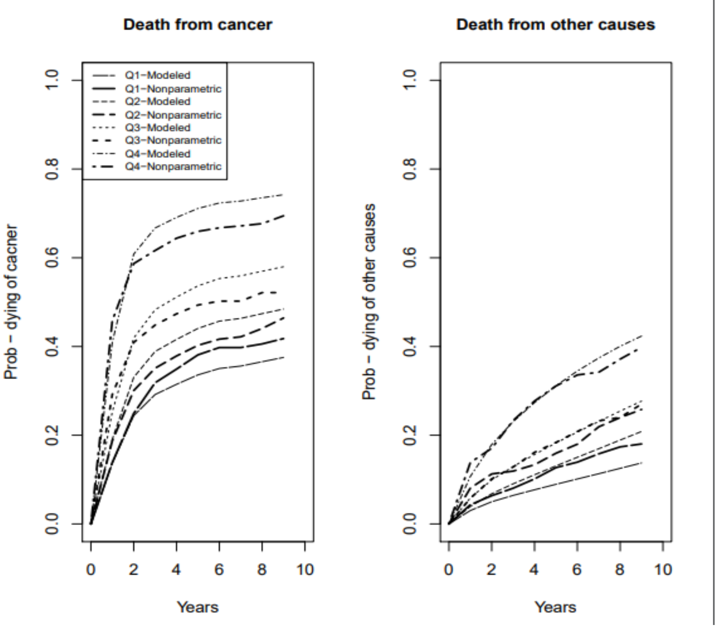

© 2023 Davies L et al. *JAMA Otolaryngology–Head & Neck Surgery*.

eFigure 20. Validation of Coexisting Condition Model for Ages 66-86, Stage IV, Based on Observed Versus Predicted Cumulative Mortality Curves for the Cancer Death Submodel and Other Cause of Death Submodel. Patients divided into quartiles in each graph based on their predicted 9-year probabilities of dying of other causes or of cancer.

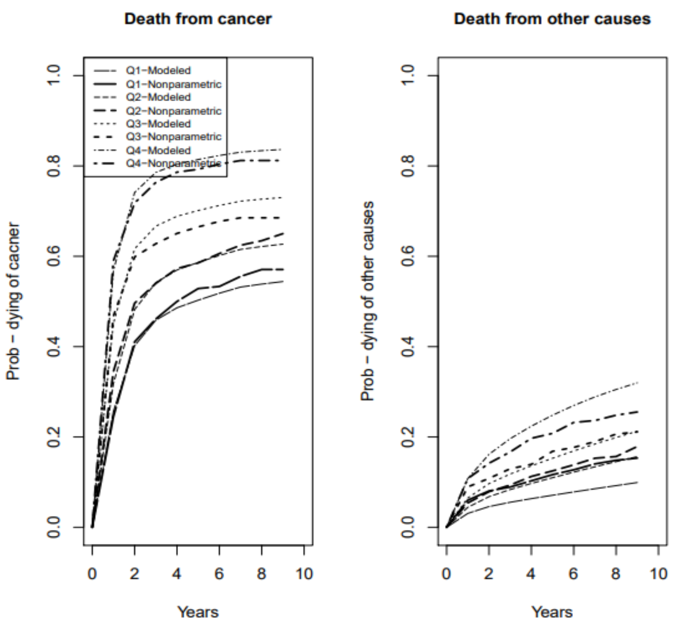

© 2023 Davies L et al. *JAMA Otolaryngology–Head & Neck Surgery*.

eFigure 21. Validation of Coexisting Condition Model for Ages 66-86, Males, Based on Observed Versus Predicted Cumulative Mortality Curves for the Cancer Death Submodel and Other Cause of Death Submodel. Patients divided into quartiles in each graph based on their predicted 9-year probabilities of dying of other causes or of cancer.

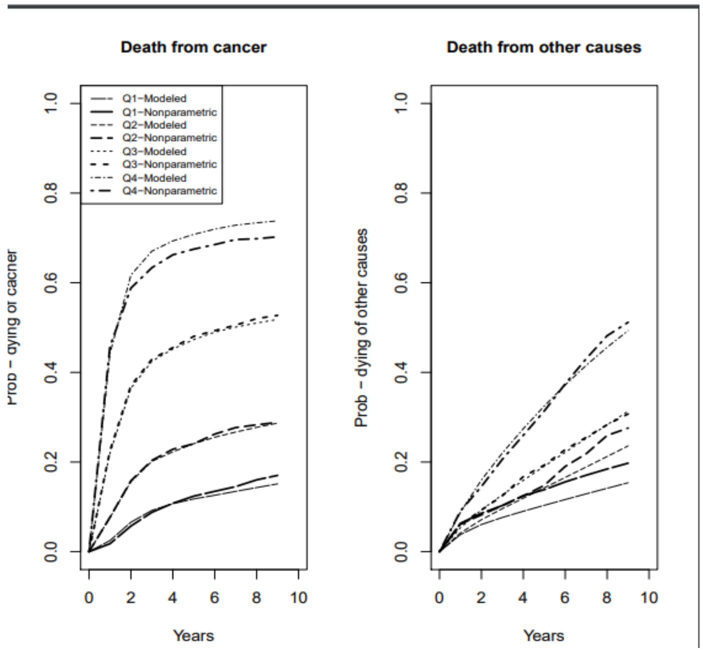

© 2023 Davies L et al. *JAMA Otolaryngology–Head & Neck Surgery*.

eFigure 22. Validation of Coexisting Condition Model for Ages 66-86, Females, Based on Observed Versus Predicted Cumulative Mortality Curves for the Cancer Death Submodel and Other Cause of Death Submodel. Patients divided into quartiles in each graph based on their predicted 9-year probabilities of dying of other causes or of cancer.

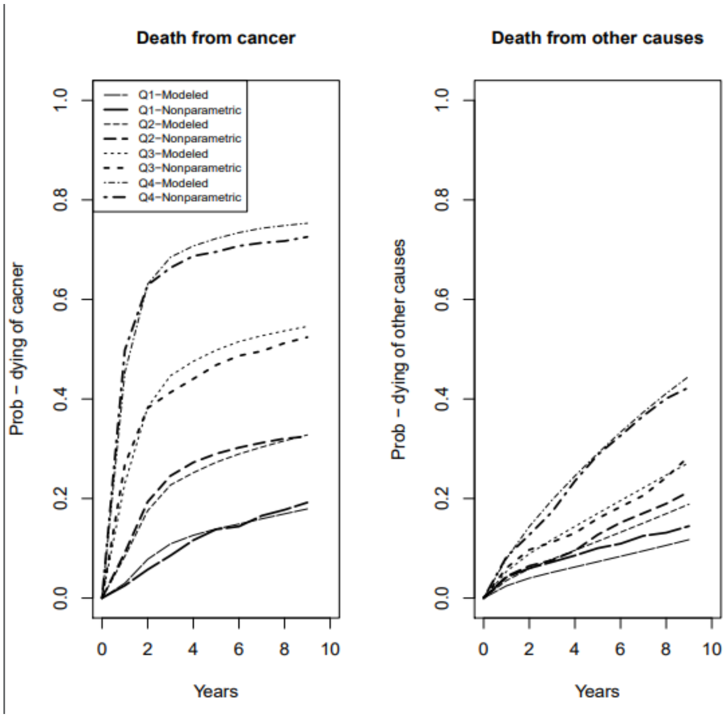

© 2023 Davies L et al. *JAMA Otolaryngology–Head & Neck Surgery.*

eTable 20. Area Under the Curve (AUC) Statistics for the Cancer Death Submodel (AUC1) and the Other Cause of Death Submodel (AUC2) of the Coexisting Condition Model (Ages 66-86) by Stage and Sex.

**Oral Cavity - All Stages 8,786 patients**			**Oral Cavity – Males 4,490 patients**			**Oral Cavity - Females 4,296 patients**		
	AUC1	AUC2		AUC1	AUC2		AUC1	AUC2
1-year	0.815	0.697	1-year	0.817	0.691	1-year	0.812	0.700
3-year	0.778	0.662	3-year	0.779	0.656	3-year	0.778	0.663
5-year	0.768	0.648	5-year	0.772	0.636	5-year	0.762	0.653
7-year	0.763	0.640	7-year	0.767	0.629	7-year	0.759	0.643
9-year	0.760	0.631	9-year	0.763	0.619	9-year	0.757	0.635
**Oral Cavity - Stage 1 2,990 patients**			**Oral Cavity - Stage 2 1,814 patients**			**Oral Cavity - Stage 3 1,387 patients**		
	AUC1	AUC2		AUC1	AUC2		AUC1	AUC2
1-year	0.733	0.717	1-year	0.732	0.691	1-year	0.697	0.682
3-year	0.686	0.703	3-year	0.666	0.653	3-year	0.643	0.665
5-year	0.677	0.696	5-year	0.657	0.629	5-year	0.636	0.637
7-year	0.676	0.676	7-year	0.650	0.627	7-year	0.634	0.612
9-year	0.673	0.656	9-year	0.651	0.609	9-year	0.635	0.600
			**Oral Cavity - Stage 4 2,595 patients**					
				AUC1	AUC2			
			1-year	0.679	0.645			
			3-year	0.645	0.618			
			5-year	0.639	0.598			
			7-year	0.639	0.583			
			9-year	0.635	0.570			

© 2023 Davies L et al. *JAMA Otolaryngology–Head & Neck Surgery.*

eFigure 23. Ten-Fold Cross-Validation Based on Observed Versus Predicted Cumulative Mortality Curves for Males for All Causes of Death (in This Case Taken to Represent Other Causes of Death) Based on the Other Cause of Death Submodel for Time From Interview. Interviewees were grouped into quartiles from best to worst prognosis based on their 9-year predicted other cause survival. NHIS data for ages 40-86 (General Health Self-Assessment Model).

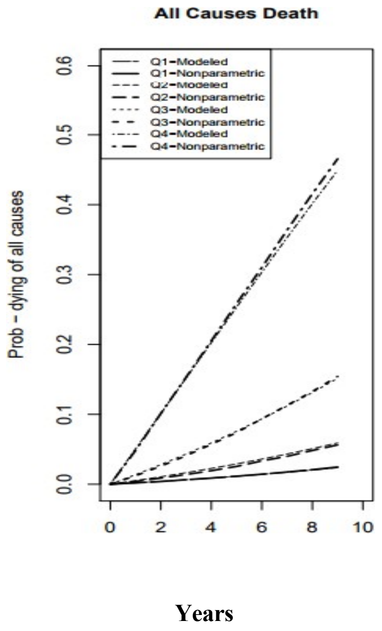

© 2023 Davies L et al. *JAMA Otolaryngology–Head & Neck Surgery*.

eFigure 24. Ten-Fold Cross-Validation Based on Observed Versus Predicted Cumulative Mortality Curves for Females for All Causes of Death (in This Case Taken to Represent Other Causes of Death) Based on the Other Cause Mortality Submodel for Time From Interview. Interviewees were grouped into quartiles from best to worst prognosis based on their 9-year predicted other cause survival. NHIS data for ages 40-86 (General Health Self-Assessment Model).

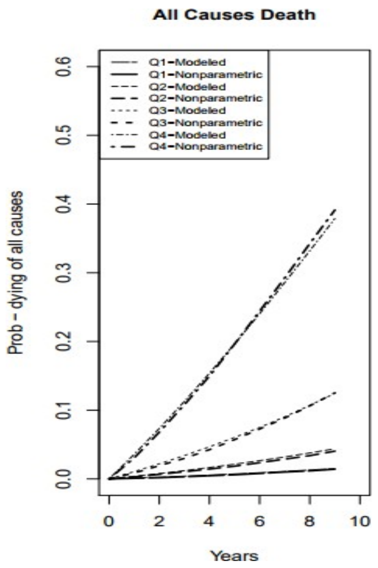

© 2023 Davies L et al. *JAMA Otolaryngology–Head & Neck Surgery*.

eTable 21. Area Under the Curve (AUC) Statistics for the Other Cause of Death Submodel of the General Health Self-Assessment Model (NHIS Data Ages 40-86) by Age and Sex.

**Males Total (144,666)**<br><br>1-year 0.816<br>3-year 0.821<br>5-year 0.826<br>7-year 0.830<br>9-year 0.835	**Male 40-54 (70,270)**<br><br>1-year 0.753<br>3-year 0.742<br>5-year 0.739<br>7-year 0.737<br>9-year 0.736	**Male 55-64 (33,312)**<br><br>1-year 0.754<br>3-year 0.735<br>5-year 0.733<br>7-year 0.735<br>9-year 0.740	**Male 65-69 (14,306)**<br><br>1-year 0.730<br>3-year 0.712<br>5-year 0.711<br>7-year 0.706<br>9-year 0.712	**Male 70-74 (11,868)**<br><br>1-year 0.717<br>3-year 0.701<br>5-year 0.693<br>7-year 0.693<br>9-year 0.701
**Male 75-79 (8,921)**<br><br>1-year 0.689<br>3-year 0.677<br>5-year 0.671<br>7-year 0.673<br>9-year 0.687	**Male 80-86 (5,989)**<br><br>1-year 0.668<br>3-year 0.642<br>5-year 0.644<br>7-year 0.661<br>9-year 0.679	**Male 50-59 (37,782)**<br><br>1-year 0.743<br>3-year 0.738<br>5-year 0.739<br>7-year 0.735<br>9-year 0.740	**Male 60-69 (30,125)**<br><br>1-year 0.751<br>3-year 0.726<br>5-year 0.723<br>7-year 0.725<br>9-year 0.728	**Male 70-79 (20,789)**<br><br>1-year 0.706<br>3-year 0.692<br>5-year 0.689<br>7-year 0.691<br>9-year 0.701
**Females Total (194,486)**<br><br>1-year 0.814<br>3-year 0.819<br>5-year 0.826<br>7-year 0.833<br>9-year 0.840	**Female 40-54 (84,840)**<br><br>1-year 0.794<br>3-year 0.781<br>5-year 0.771<br>7-year 0.759<br>9-year 0.759	**Female 55-64 (42,501)**<br><br>1-year 0.769<br>3-year 0.730<br>5-year 0.732<br>7-year 0.732<br>9-year 0.735	**Female 65-69 (19,936)**<br><br>1-year 0.746<br>3-year 0.731<br>5-year 0.719<br>7-year 0.717<br>9-year 0.716	**Female 70-74 (18,559)**<br><br>1-year 0.715<br>3-year 0.702<br>5-year 0.696<br>7-year 0.691<br>9-year 0.692
**Female 75-79 (15,884)**<br><br>1-year 0.680<br>3-year 0.663<br>5-year 0.667<br>7-year 0.664<br>9-year 0.667	**Female 80-86 (12,766)**<br><br>1-year 0.633<br>3-year 0.642<br>5-year 0.641<br>7-year 0.652<br>9-year 0.669	**Female 50-59 (46,708)**<br><br>1-year 0.782<br>3-year 0.747<br>5-year 0.748<br>7-year 0.745<br>9-year 0.743	**Female 60-69 (40,369)**<br><br>1-year 0.758<br>3-year 0.727<br>5-year 0.723<br>7-year 0.723<br>9-year 0.725	**Female 70-79 (34,443)**<br><br>1-year 0.697<br>3-year 0.689<br>5-year 0.691<br>7-year 0.690<br>9-year 0.692

© 2023 Davies L et al. *JAMA Otolaryngology–Head & Neck Surgery.*

eTable 22. Position of the SEER OCSC in Relation to Other Available Oral Cancer Calculators

Author, publication and url where applicable	Description
**For use at time of diagnosis – survival prediction**	
SEER Oral Cancer Survival Calculator<br>(this tool)	Interactive model. Health status adjusted age, probability of surviving, dying of cancer, dying of other causes.<br>Age, tumor stage, lymph node stage, metastatic stage, histologic differentiation, smoking status, self-assessed general health, Comorbidity (Charlson categories), Area of residence.
Bobdey et al, 2016[19]<br>Patients diagnosed with cancer 2006-2008<br>https://www.ncbi.nlm.nih.gov/pubmed/27227458	Nomogram. Overall survival prediction.<br>Age, comorbidity (present or absent), lymph node metastases (present or absent), stage, perineural invasion (present or absent), tumor thickness, histologic differentiation.
Montero et al, 2014[20]<br>Patients diagnosed with cancer 1985-2009<br>https://www.ncbi.nlm.nih.gov/pubmed/24399417	Nomograms. Overall survival probability, cancer specific mortality probability, recurrence free survival probability.<br>Age, comorbidity (Washington University Head and Neck Comorbidity Index), primary site size, lymph node metastases (present or absent), tobacco (current vs. none/former), race, bone invasion (present/absent)
Emerick et al, 2011[21]<br>Patients diagnosed with cancer 1980 - 2009<br>http://www.ncbi.nlm.nih.gov/pubmed/23797612<br>Lifemath.net/cancer/headneck/outcome	Kaplan-Meier. Ten year cancer mortality, mean overall survival conditional to surviving to a specific age past diagnosis, mean survival in the absence of cancer.<br>Current age, sex, race, years since diagnosis, tumor size, localized vs. T4a or T4b, lymph node stage, number of lymph nodes positive, histologic type
Datema, 2013[22]<br>Patient data continuously updated<br>https://www.ncbi.nlm.nih.gov/pubmed/22847987<br>http://www.oncologiq.nl	Kaplan-Meier. Ten year overall survival probability.<br>Age, smoking status (current vs. none vs. former), gender, smoking pack years, BMI, clinical T (tumor) and N (nodal) stage, comorbidity (ACE), WHO performance status, employment status, weight loss in past 6 months
**For use post-surgery, considering adjuvant radiation**	
Gross et al, 2008[23]<br>Wang, et al, 2013[24]<br>Patients diagnosed with cancer 1988-1998<br>http://www.ncbi.nlm.nih.gov/pubmed/18720518<br>https://dmice.ohsu.edu/nomograms/	Lognormal survival model. Predicted 5 year locoregional recurrence free survival with and without adjuvant radiotherapy.<br>Age, Sex, Smoking status, site (floor of mouth vs other), tumor stage, lymph node stage, histologic grade, margin status
**For use post treatment - conditional survival post treatment**	
Wang et al, 2011[25]<br>Patients diagnosed with cancer 1988-1998<br>http://www.ncbi.nlm.nih.gov/pubmed/21493289<br>https://dmice.ohsu.edu/nomograms/headneck/headneck.php	Conditional survival prediction.<br>Age, race, sex, overall stage (SEER Historic), histologic grade, time already survived

© 2023 Davies L et al. *JAMA Otolaryngology–Head & Neck Surgery.*

eAppendix 7. Applicability of Mortality and Survival Data Over Time

The included cancer data in the SEER OCSC are from 2000-2011. Changes in risk factors, treatment patterns, diagnostic precision or care for other conditions may affect observed mortality and survival, affecting utility of the SEER OCSC. To test this, survival and mortality analyses were completed to assess whether major changes had been observed. Analyses were performed using data from "SEER Research Plus Data, 18 Registries, Nov 2020 Sub (2000-2018)". Overall, There have been only small degrees of change, suggesting that estimates obtained from the calculator are not likely to be substantially different than what current trends would indicate.

Mortality: Reflecting secular trends of decreasing tobacco use, which affects the population at risk of both getting and dying from oral cancer, the incidence per 100,000 of oral cancer has fallen slightly in 2012-2018 compared to 2000-2011: 4.11 (95% CL 4.06, 4.16) to 4.08 (95% CL 4.02, 4.14). In parallel, mortality for these two time periods has also fallen slightly for the two time periods, from 3.57 for 2000-2011 (95% CL 3.55, 3.59) to 3.52 (95% CL 3.49, 3.55) for the period 2012-2017.

Survival: The event of interest is oral cavity cancer cause of death. In the model, the years of diagnosis are grouped into 3 categories: 2000-2003; 2004-2007; and 2008-2011, which may be collapsed or eliminated during model selection. The period 2008-2011 is considered as most recent data and the prediction for these most recent diagnosis years are used to produce survival estimates for the calculator. There is marginally significant, but very small improvement in the survival for 2012-2017 compared to 2008-2011 (eFigure 25, 83.8% vs 83.9%, 68.1% vs 70.0%, and 63.4% vs 65.0% at 1, 3, and 5 years respectively).

We further examined the cumulative survival for each single year of diagnosis (eFigure 26). There is no clear pattern of improvement in the cumulative survival over the year of diagnosis.

© 2023 Davies L et al. *JAMA Otolaryngology–Head & Neck Surgery.*

eFigure 25. Comparison of Survival Curves 2008-2011 vs 2012-2017

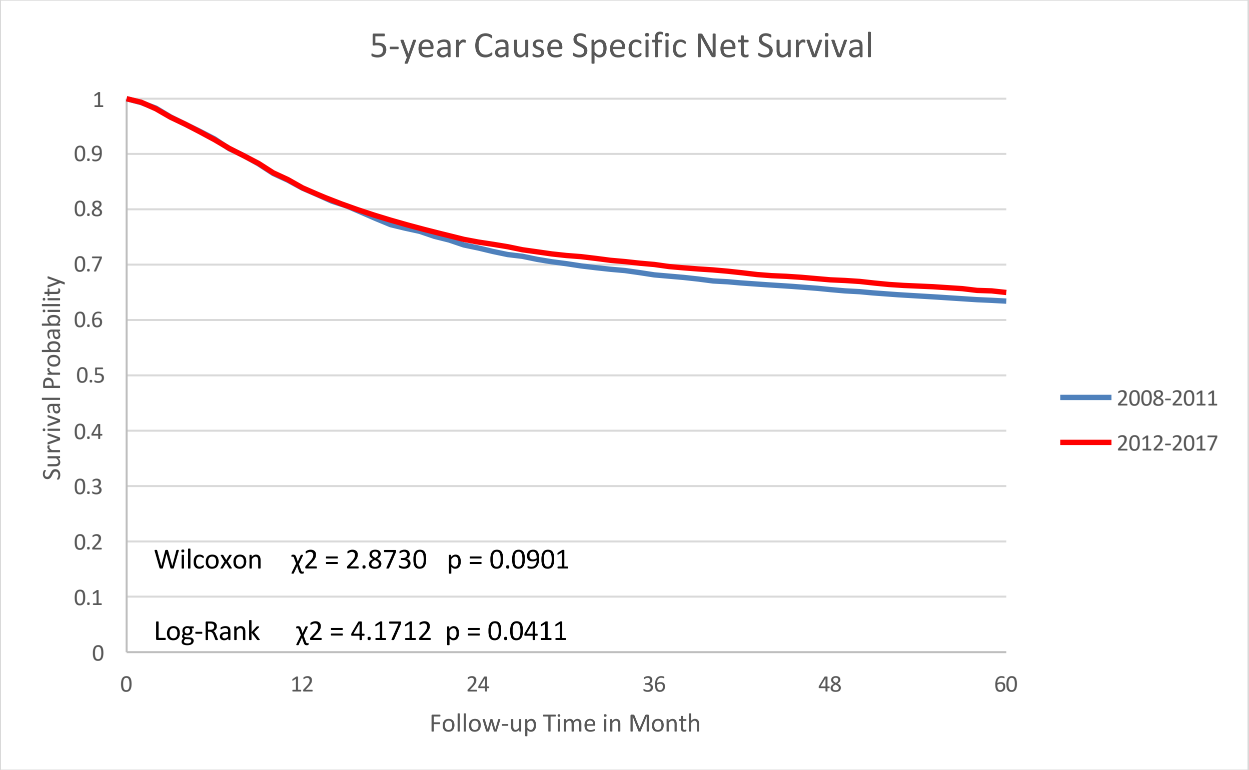

© 2023 Davies L et al. *JAMA Otolaryngology–Head & Neck Surgery.*

eFigure 26. Survival by Year of Diagnosis

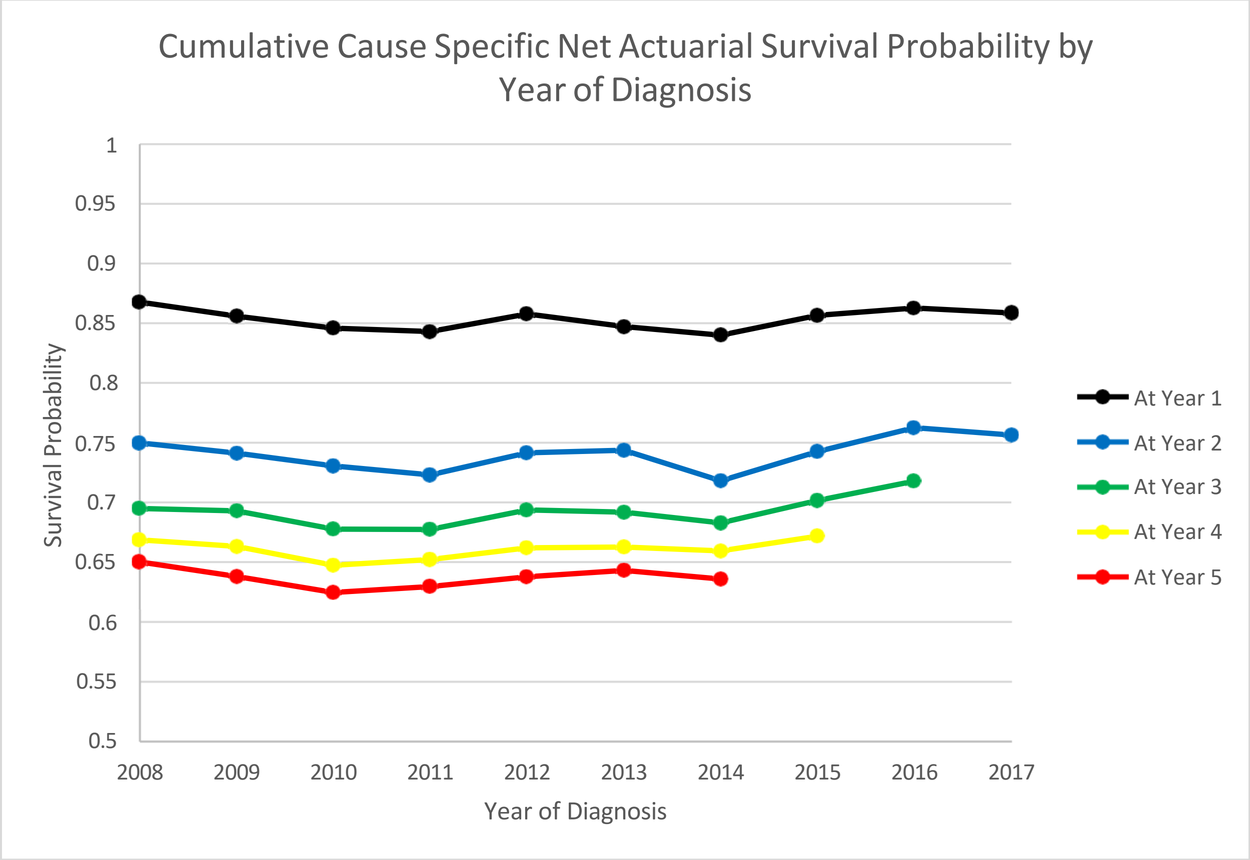

© 2023 Davies L et al. *JAMA Otolaryngology–Head & Neck Surgery.*

eReferences

1. Yu M, Tatalovich Z, Gibson JT, Cronin KA. Using a composite index of socioeconomic status to investigate health disparities while protecting the confidentiality of cancer registry data. *Cancer Causes Control.* 2014;25(1):81-92.
2. Yost K, Perkins C, Cohen R, Morris C, Wright W. Socioeconomic status and breast cancer incidence in California for different race/ethnic groups. *Cancer Causes Control.* 2001;12(8):703-711.
3. Mariotto AB, Wang Z, Klabunde CN, Cho H, Das B, Feuer EJ. Life tables adjusted for comorbidity more accurately estimate noncancer survival for recently diagnosed cancer patients. *Journal of clinical epidemiology.* 2013;66(12):1376-1385.
4. Charlson ME, Pompei P, Ales KL, MacKenzie CR. A new method of classifying prognostic comorbidity in longitudinal studies: development and validation. *J Chronic Dis* 1987; 40(5):373-383.
5. McCallum J, Shadbolt B, D. W. Self-rated health and survival: a 7-year follow-up study of Australian elderly. *American Journal of Public Health* 1994; 84: 1100-1105.
6. Idler EL, Benyamini Y. Self-rated health and mortality: a review of twenty-seven community studies. *Journal of Health and Social Behavior* 1997; 38: 21-37.
7. Idler EL, Russell LB, Davis D. Survival, functional limitations, and self-rated health in the NHANES I Epidemiologic Follow-up Study, 1992. *American Journal of Epidemiology* 2000; 152: 874-883.
8. DeSalvo KB, Bloser N, Reynolds K, He J, Muntner P. Mortality prediction with a single general self-rated health question. A meta-analysis. *Journal of general internal medicine.* 2006;21(3):267-275.
9. Hoffman RM, Koyama T, Albertsen PC, et al. Self-Reported Health Status Predicts Other-Cause Mortality in Men with Localized Prostate Cancer: Results from the Prostate Cancer Outcomes Study. *Journal of general internal medicine.* 2015;30(7):924-934.
10. Cheng SC, Fine JP, Wei LJ. Prediction of cumulative incidence function under the proportional hazards model. Biometrics 1998; 54: 219–228.
11. Lee M, Gouskova NA, Feuer EJ, Fine JP. On the choice of time scales in competing risks predictions. *Biostatistics* 2017; 18: 15-31.
12. Lee M, Feuer EJ, Fine JP. On the analysis of discrete time competing risks data. *Biometrics* 2018; 74: 1468–1481.
13. Lee M, Feuer EJ, Wang Z, Cho H, Zou Z, Hankey BF, Mariotto AB, Fine JP. Analyzing discrete competing risks data with partially overlapping or independent data sources and non-standard sampling schemes, with application to cancer registries. *Statistics in Medicine* 2019; 38: 5528-5546.
14. Howlader N, Ries LA, Mariotto AB, Reichman ME, Ruhl J, Cronin KA. Improved estimates of cancer-specific survival rates from population-based data. J Natl Cancer Inst. 2010 Oct 20;102(20):1584-98.
15. Green FL, Page DL, Fleming ID et al (Editors), AJCC Cancer Staging Manual (Eighth Edition), American College of Surgeons, Last Updated 05 June 2018.
16. Lee M, Cronin KA, Gail MH, Feuer EJ. Predicting the absolute risk of dying from colorectal cancer and from other causes using population-based cancer registry data. *Statistics in Medicine* 2012; 31: 489-500.
17. Aalen, O.O. and Johansen, S. (1978) An Empirical Transition Matrix for Non-Homogeneous Markov Chains Based on Censored Observations. *Scandinavian Journal of Statistics*, 5, 141-15.
18. Gerds TA, Andersen PK, Kattan MW. Calibration plots for risk prediction models in the presence of competing risks. *Statistics in Medicine* 2014; 33: 3191–3203.
19. Bobdey S, Balasubramaniam G, Mishra P. Nomogram prediction for survival of patients with oral cavity squamous cell carcinoma. Head Neck. 2016;38(12):1826-1831.
20. Montero PH, Yu C, Palmer FL, et al. Nomograms for preoperative prediction of prognosis in patients with oral cavity squamous cell carcinoma. Cancer. 2014;120(2):214-221.
21. Emerick KS, Leavitt ER, Michaelson JS, Diephuis B, Clark JR, Deschler DG. Initial clinical findings of a mathematical model to predict survival of head and neck cancer. Otolaryngol Head Neck Surg. 2013;149(4):572-578.
22. Datema FR, Ferrier MB, Vergouwe Y, et al. Update and external validation of a head and neck cancer prognostic model. Head Neck. 2013;35(9):1232-1237.
23. Gross ND, Patel SG, Carvalho AL, et al. Nomogram for deciding adjuvant treatment after surgery for oral cavity squamous cell carcinoma. Head Neck. 2008;30(10):1352-1360.

© 2023 Davies L et al. *JAMA Otolaryngology–Head & Neck Surgery.*

24. Wang SJ, Patel SG, Shah JP, et al. An oral cavity carcinoma nomogram to predict benefit of adjuvant radiotherapy. JAMA otolaryngology-- head & neck surgery. 2013;139(6):554-559.
25. Wang SJ, Wissel AR, Ord CB, et al. Individualized estimation of conditional survival for patients with head and neck cancer. Otolaryngol Head Neck Surg. 2011;145(1):71-73.

© 2023 Davies L et al. *JAMA Otolaryngology–Head & Neck Surgery*.
