# Supplementary material for: A New Personalized Oral Cancer Survival Calculator to Estimate Risk of Death From Both Oral Cancer and Other Causes
Source: JAMA Otolaryngol Head Neck Surg. 2023 Jul 10;149(11):993–1000. doi: 10.1001/jamaoto.2023.1975 (PMC10334297; doi:10.1001/jamaoto.2023.1975)
Supplement: Supplement 2. — Data Sharing Statement [file jamaotolaryngolheadnecksurg-e231975-s002.pdf]

Data Sharing Statement

Davies. A New Personalized Oral Cancer Survival Calculator to Estimate Risk of Death From Both Oral Cancer and Other Causes. *JAMA Otolaryngol Head Neck Surg*. Published July 10, 2023. doi:10.1001/jamaoto.2023.1975

Data
Data available: No

Additional Information
Explanation for why data not available: The data are available publicly with a data use agreement from the data owner (e.g., National Health Survey, Medicare)
